# LC-MS Profile, Gastrointestinal and Gut Microbiota Stability and Antioxidant Activity of *Rhodiola rosea* Herb Metabolites: A Comparative Study with Subterranean Organs

**DOI:** 10.3390/antiox9060526

**Published:** 2020-06-16

**Authors:** Daniil N. Olennikov, Nadezhda K. Chirikova, Aina G. Vasilieva, Innokentii A. Fedorov

**Affiliations:** 1 Laboratory of Medical and Biological Research, Institute of General and Experimental Biology, Siberian Division, Russian Academy of Science, 6 Sakh’yanovoy Street, Ulan-Ude 670047, Russia; 2 Department of Biology, Institute of Natural Sciences, North-Eastern Federal University, 58 Belinsky Street, Yakutsk 677027, Russia; hofnung@mail.ru (N.K.C.); aina_vasilieva@mail.ru (A.G.V.); 3 Institute for Biological Problems of Cryolithozone, Siberian Division, Russian Academy of Science, 41 Lenina Street, Yakutsk 677000, Russia; fedorovia1958@mail.ru

**Keywords:** *Rhodiola rosea*, phenolic compounds, antioxidant activity, gastrointestinal digestion, gut microbiota, herbal products

## Abstract

Golden root (*Rhodiola rosea* L., Crassulaceae) is a famous medical plant with a one-sided history of scientific interest in the roots and rhizomes as sources of bioactive compounds, unlike the herb, which has not been studied extensively. To address this deficiency, we used high-performance liquid chromatography with diode array and electrospray triple quadrupole mass detection for comparative qualitative and quantitative analysis of the metabolic profiles of *Rhodiola rosea* organs before and after gastrointestinal digestion in simulated conditions together with various biochemical assays to determine antioxidant properties of the extracts and selected compounds. *R. rosea* organs showed 146 compounds, including galloyl *O*-glucosides, catechins, procyanidins, simple phenolics, phenethyl alcohol derivatives, (hydroxy)cinnamates, hydroxynitrile glucosides, monoterpene *O*-glucosides, and flavonol *O*-glycosides, most of them for the first time in the species. The organ-specific distribution of compounds found for catechins, procyanidins, and cinnamyl alcohols and glucosides was typical for underground organs and flavonoids and galloylated glucoses concentrated in the herb. Extracts from rhizomes, leaves and flowers showed high phenolic content and were effective scavengers of free radicals (2,2-diphenyl-1-picrylhydrazyl (DPPH^•^), 2,2′-azino-bis(3-ethylbenzothiazoline-6-sulfonic acid) (ABTS^•+^), O_2_^•−^, ^•^OH) and protected β-carotene in a bleaching assay. Digestion in the gastric and intestine phase influenced the composition of *R. rosea* extracts negatively, affecting the content of catechins, procyanidins, and galloyl glucoses, and therefore, the antioxidativity level. After gut microbiota treatment, the antioxidant capacity of rhizome extract was lower than leaves and flowers due to the aglycone composition found in the colonic phase of digestion. Our study demonstrated that the herb of *R. rosea* is a rich source of metabolites with high antioxidant properties and could be a valuable plant for new bioactive products.

## 1. Introduction

Plants have a long history of medical use by humans, leading to the creation of various fields of biomedical knowledge. In recent decades, the importance of plant-derived drugs has risen significantly, which has caused a marked increase in inquiry into wild species. One popular plant is *Rhodiola rosea* L. (golden root, roseroot; synonym *Sedum roseum* (L.) Scop., Crassulaceae family), a medicinal species with a disjunct distribution in Eurasia and known as a medical remedy [1]. The roots and rhizome of *R. rosea* are the source of numerous metabolites, like acyclic alcohol derivatives, benzyl glucosides, phenols, hydroxycinnamates, gallotannins, flavonoids, catechins, procyanidins, and terpenes [2], with a diversity of bioactivities as antioxidant [3], anticancer [4], antidiabetic [5], antidepressant, neuroprotective [6], anti-inflammatory [7], and adaptogenic [8] agents. The main reserves of *R. rosea* are concentrated in Siberian regions such as Altai, Western Sayans, and Tuva [9]. The productivity of underground organs in these areas in the 1970s was estimated at 1600–1700 tons per year but uncivilized collecting and slow regeneration of the roots and rhizomes (15–20 years) reduced this level to 40–60 tons per year and reclassified of *R. rosea* as a vulnerable species [10]. Introduction events [11] and the development of biotechnological methods cultivating *R. rosea* tissues [12] are widely used for addressing the problems but are not enough to satisfy market needs.

In the process of industrially gathering *R. rosea* plants, both in nature and cultured, the organs of interest are the roots and rhizomes (underground part), which are collected to the detriment of aerial organs (herb) that remain unused. The leaves, flowers, and stems can also be the useful sources of bioactive metabolites and, in contrast to roots and rhizome, their gathering does not lead to the destruction of natural reserves. By accessing the known scientific information devoted to the study of chemical analysis, compound isolation and bioactivity of *R. rosea* organs, it is commonly observed that roots and rhizomes are studied much more than the herb (Table 1). Over a hundred compounds are found for the *R. rosea* plant [2] and only twenty metabolites (mostly flavonoids) belong to herb [13,14,15]. Furthermore, there are no bioactivity data about *R. rosea* herb, making it much more difficult to specify its biomedical properties. This is also the case for most of the other *Rhodiola* species, whose herbs are still poorly understood; some flavonoids were found in *R. litvinovii* [16] and *R. quadrifida* herbs [17], arbutin and 6′-*O*-galloyl arbutin were found in *R. coccinea* herb [18], tyrosol in *R. quadrifida* herb [19] and salidroside in *R. sachalinensis* herb [20]. With such a variety of compounds detected in rhodiola roots and rhizomes (about 300) [2], the chemistry of rhodiola herbs is badly in need of new data. In addition to *R. rosea* chemistry, aspects of the study of bioavailability and gastrointestinal transformation of the basic active components require greater attention because the known information mainly focused on the active compounds salidroside [4] and rosavin [21].

This paper aims to estimate the chemical and biomedical prospects of *R. rosea* herb as a possible future remedy. Thus, we realized the first comparative analysis of the metabolic profiles of *R. rosea* organs using the high-performance liquid chromatography with diode array and electrospray triple quadrupole mass detection (HPLC-DAD-ESI-QQQ-MS) technique, both in qualitative and quantitative mode. The data about the stability of the selected compounds of *R. rosea* extracts in the simulated gastrointestinal model were added as an extra row to understanding the basic differences of metabolites in underground and aerial parts after digestion. Considering that most metabolites found in *R. rosea* were phenolics, we studied the variation of the antioxidant properties of *R. rosea* extracts as a function of organ profile and digestion phase. To the best of our knowledge, this is the first comprehensive study of *R. rosea* whole plant metabolites, their digestion transformation, and antioxidant activity.

## 2. Materials and Methods

### 2.1. Plant Materials and Chemicals

Samples of *Rhodiola rosea* were collected in Sakha (Yakutia) Republic in the flowering period (herbal organs) and seedling period (subterranean organs) (Table 2). The species were authenticated by Prof. T.A. Aseeva (IGEB SB RAS, Ulan-Ude, Russia). Plant material was dried and powdered before analysis.

The reference compounds were purchased from BioBioPha (Kunming, Yunnan, PRC); ChemFaces (Wuhan, Hubei, PRC); Extrasynthese (Lyon, France); Sigma-Aldrich (St. Louis, MO, USA); Toronto Research Chemicals (North York, ON, Canada); Research Institute of Medical and Aromatic Plants (Moscow, Russia); VulcanChem (Pasadena, CA, USA) (Table S1). Selected chemical were from Sigma-Aldrich (St. Louis, MO, USA)—acetonitrile for HPLC (Cat. No 34851, ≥99.9%), lithium perchlorate (Cat. No. 431567, ≥99%), methanol (Cat. No. 322415, ≥99.8%), pancreatin from porcine pancreas (Cat. No. P7545, 8 × USP specifications), pepsin from porcine gastric mucosa (Cat. No. P6887, 3200-4500 units/mg protein), perchloric acid 70% (Cat. No. 311421, ≥99%), trolox (Cat. No. 238813, ≥97%). Gossypetin 7-*O*-(3″-*O*-glucosyl)-rhamnoside (=rhodioflavonoside), herbacetin 7-*O*-(3″-*O*-glucosyl)-rhamnoside (=rhodiosin), gossypetin 7-*O*-rhamnoside-8-*O*-glucoside (=rhodiolgidin), herbacetin-8-*O*-xyloside (=rhodalin), herbacetin 7-*O*-rhamnoside-8-*O*-glucoside (=rhodionidin), gossypetin 3-*O*-glucoside-8-*O*-glucuronide, gossypetin 3-*O*-(3″-*O*-acetyl)-glucoside-8-*O*-glucuronide (=rhodiquadrin B), herbacetin 8-*O*-(2″-*O*-glucosyl)-glucuronide (=rhodiquadrin C), herbacetin 3-*O*-glucoside-8-*O*-glucuronide, herbacetin 8-*O*-glucuronide, rhodiocyanoside A were isolated previously from *Rhodiola* species [17,22]. Equipment used for UV-Vis spectrophotometry was SF-2000 UV-Vis-spectrophotometer (OKB Specter, St. Petersburg, Russia).

### 2.2. Chemical Composition Analysis of R. rosea Organs and Antioxidant Activity Assays

UV-Vis spectrophotometrical assays were used to determine the total content of flavonoids (as mg/g quercetin equivalents) [23], catechins (as mg/g (+)-catechin equivalents) [24], procyanidins (as mg/g procyanidin B_1_ equivalents) [25], phenylpropanoids (as mg/g rosavin equivalents) [26], gallotannins (as mg/g gallic acid equivalents) [27], ellagitannins (as mg/g ellagic acid equivalents) [28], coumarins (as mg/g umbelliferon equivalents) [29], and anthocyanes (as mg/g cyanidin-3-*O*-glucoside equivalents) [30] in dry herbal samples of *R. rosea* (roots, rhizomes, leaves, flowers, stems). All the analyses were carried out in triplicate and the data were expressed as mean value ± standard deviation (SD).

Antioxidant activity of total extracts and selected compounds was determined using spectrophotometric assays. Trolox was used as a positive control (PC; 10 mg/mL), and water was used as a negative control (NC). Scavenging activity against 2,2-diphenyl-1-picrylhydrazyl radicals (DPPH^•^) was studies as the following assay: 500 μL DPPH^•^ (freshly prepared MeOH solution, 100 μg/mL) and 500 μL of *Rhodiola rosea* extract (freshly prepared 50% MeOH solution, 1–200 μg/mL) or pure compound (freshly prepared MeOH solution, 1–200 μg/mL). Absorbance (520 nm) was measured after 15 min. The DPPH^•^ scavenging capacity was calculated using equation: Scavenging capacity (%) = ((A_520_^NC^ – A_520_^PC^) – (A_520_^Sample^ – A_520_^PC^)/(A_520_^NC^ – A_520_^PC^)) × 100, where A_520_^NC^ is the absorbance of the negative control, A_520_^PC^ is the absorbance of the positive control, and A_520_^Sample^ is the absorbance of the sample solution. For studing 2,2′-azino-bis(3-ethylbenzothiazoline-6-sulfonic acid) cation radicals (ABTS^•^^+^) scavenging capacity ABTS (water solution; 7 mM) reacted with potassium persulphate (water solution; final concentration 2.45 mM) in the dark at 20 °C (12–16 h before use). The ABTS^•^^+^ solution was diluted with MeOH to an absorbance of 0.70 at 734 nm and equilibrated at 20 °C. *Rhodiola rosea* extract (500 μL; freshly prepared 50% MeOH solution, 1–200 μg/mL) was mixed with ABTS^•^^+^ solution (500 μL) and the absorbance was measured at 734 nm after 20 min. The ABTS^•^^+^ scavenging capacity was calculated using equation: Scavenging capacity (%) = ((A_734_^NC^ – A_734_^PC^) – (A_734_^Sample^ – A_734_^PC^)/(A_734_^NC^ – A_734_^PC^)) × 100, where A_734_^NC^ is the absorbance of the negative control, A_734_^PC^ is the absorbance of the positive control, and A_734_^Sample^ is the absorbance of the sample solution. Superoxide radicals (O_2_^•^^−^) scavenging capacity was determined using *Rhodiola rosea* extract (50 μL; freshly prepared solution in Tris-HCl buffer, 0.05 M, pH 8.2; 10–1000 μg/mL) mixed with pyrogallol (50 μL, 6 mM) and Tris-HCl buffer (1 mL). The absorbance was measured at 325 nm after 5 min. The O_2_^•−^ scavenging capacity was calculated using equation: Scavenging capacity (%) = ((A_325_^NC^ – A_325_^PC^) – (A_325_^Sample^ – A_325_^PC^)/(A_325_^NC^ – A_325_^PC^)) × 100, where A_325_^NC^ is the absorbance of the negative control, A_325_^PC^ is the absorbance of the positive control, and A_325_^Sample^ is the absorbance of the sample solution. To determine hydroxyl radicals (^•^OH) scavenging capacity *Rhodiola rosea* extract (100 μL; freshly prepared solution in 0.2 M phosphate buffer (pH 7.4; 1–500 μg/mL) mixed with deoxyribose solution in the same buffer (100 μL; 2.8 mM), H_2_O_2_ (10 μL; 3.6 mM), FeCl_3_ (10 μL; 5.0 mM) and EDTANa_2_ (100 μL; 100 μM). After addition of ascorbic acid (50 μL; 200 μM) the mixture was incubated at 55 °C for 20 min. Finally, 2-thiobarbituric acid (800 μL; 10 mg/mL) and trichloroacetic acid (800 μL; 50 mg/mL) were added and heated at 95 °C for 20 min. The absorbance was measured at 530 nm. The ^•^OH scavenging capacity was calculated using equation: Scavenging capacity (%) = ((A_530_^NC^ – A_530_^PC^) – (A_530_^Sample^ – A_530_^PC^)/(A_530_^NC^ – A_530_^PC^)) × 100, where A_530_^NC^ is the absorbance of the negative control, A_530_^PC^ is the absorbance of the positive control, and A_530_^Sample^ is the absorbance of the sample solution. The IC_50_ value is the effective concentration at which free radicals (DPPH^•^, ABTS^•^^+^, O_2_^•−^, ^•^OH) was scavenged by 50%. Values are expressed as mean obtained from five independent experiments. Carotene bleaching assay was performed as described previously using β-carotene as a substrate (Sigma-Aldrich, St. Louis, MO, USA, cat. No. C9750) [31].

### 2.3. Total Extract Preparation from R. rosea Organs

For the preparation of the total extract of *R. rosea* organs, the dry and powdered sample of the organ (100 g) was extracted twice with stirring in a glass flask (2 L) with 70% methanol (1 L) using an ultrasonic bath (80 min, 50 °C, ultrasound power 100 W, frequency 35 kHz). The extracts were passed through a cellulose filter, concentrated under reduced pressure until dryness, and stored at 4 °C before using for the chemical analysis of biological activity study. The yields of total extracts of *R. rosea* were 32.5 g (roots), 22.5 g (rhizome), 20.0 g (leaves), 25.0 g (flowers), 9.0 g (stems).

### 2.4. Solid-Phase Extratcion (SPE) of Total Extract from R. rosea Organs

Cascade of two SPE cartridges Sep-Pak tC_18_ (50 mg, 37–55 µm) followed to Sep-Pak C_18_ (360 mg, 55–105 µm; Waters Corp., Millford, MA, USA) both preconditioned with methanol (30 mL) and water (50 mL) used to separate catechins, procyanidins and galloyl glucoses (tannin related compounds) from other small molecules. The sample of total extract of *R. rosea* organ (50 mg) ultrasonically dissolved in tridistilled water (10 mL), centrifuged (6000× *g*, 15 min), and the final solution passed through a cascade of SPE cartridges. Elution was started with water (pH 6.8–7.2, 30 mL), then the cartridges were dried with N_2_, and the targeted compounds eluted with ethyl acetate–methanol mixture (5:1, 30 mL). The organic eluate was concentrated in vacuo until dryness, redissolved in methanol (5 mL), and stored at 4 °C before HPLC-DAD-ESI-QQQ-MS analysis.

Non-tannin related compounds were separated on the polyamide cartridges Chromabond (Polyamide 6, 6 mL, 1000 mg; Sorbent Technologies, Inc., Norcross, GA, USA) preconditioned with methanol (50 mL) and water (70 mL). The sample of total extract of *R. rosea* organ (80 mg) ultrasonically dissolved in tridistilled water (10 mL), centrifuged (6000× *g*, 15 min), and the final solution passed through polyamide cartridge eluted with water (30 mL; eluate I), 70% methanol (40 mL; eluate II) and 0.5% NH_3_ in methanol (40 mL; eluate III). The organic eluate was concentrated in vacuo until dryness, redissolved in methanol (5 mL), and stored at 4 °C before HPLC-DAD-ESI-QQQ-MS analysis. Expected elution of simple phenolics, phenethyl alcohol derivatives, (hydroxy)cinnamoyl glucosides, hydroxynitrile glucosides, and monoterpene *O*-glucosides was found in eluate I, neutral flavonol *O*-glucosides in eluate II, and acidic flavonol *O*-glucosides, acylated flavonol *O*-glucosides and hydrocycinnamates in eluate III.

### 2.5. High-Performance Liquid Chromatography with Diode Array Detection and Electrospray Ionization Triple Quadrupole Mass Spectrometric Detection (HPLC-DAD-ESI-QQQ-MS)

Reversed-phase high-performance liquid chromatography with diode array detection and electrospray ionization triple quadrupole mass spectrometric detection (HPLC-DAD-ESI-QQQ-MS) was used for phenolic profiling. Experiments were performed on an LCMS 8050 liquid chromatograph coupled with diode-array-detector and triple-quadrupole electrospray ionization detector (Shimadzu, Columbia, MD, USA) coupled with GLC Mastro C18 column (150 × 2.1 mm, Ø 3 μm; Shimadzu, Kyoto, Japan) at the column temperature 35 °C. Eluent A was 0.5% formic acid in water and eluent B was 0.5% formic acid in acetonitrile. The injection volume was 1 μL, and elution flow was 100 μL/min. Gradient program for Sep-Pak C18 eluates (mode 1): 0.0–2.5 min 3.0–12.0% B, 2.5–5.0 min 12.0–25.0% B, 5.0–11.0 min 25.0–41.0% B, 11.0–15.0 min 41.0–64.0% B, 15.0–16.0 min 64.0–3.0% B, 16.0–20.0 min 3.0% B; gradient program for polyamide eluates I (mode 2): 0.0–2.0 min 11.0–12.5% B, 2.0–6.5 min 12.5–21.0% B, 6.5–10.0 min 21.0–23.0% B, 10.0–15.0 min 23.0–28.0% B, 15.0–17.0 min 28.0–34.0% B, 17.0–18.0 min 34.0–11.0% B, 18.0–25.0 min 11.0% B; gradient program for polyamide eluates II (mode 3): 0.0–4.0 min 5.0–14.0% B, 4.0–8.0 min 14.0–24.0% B, 8.0–15.0 min 24.0–35.0% B, 15.0–16.0 min 35.0–5.0% B, 16.0–22.0 min 5.0% B; gradient program for polyamide eluates III (mode 4): 0.0–4.0 min 5.0–11.0% B, 4.0–10.0 min 11.0–18.0% B, 10.0–17.0 min 18.0–29.0% B, 17.0–19.0 min 29.0–5.0% B, 19.0–25.0 min 5.0% B. The DAD acquisitions were performed in the range of 200–600 nm. MS detection was performed in negative and positive ESI mode using the parameters as follows: temperature levels of ESI interface, desolvation line, and heat block were 300 °C, 250 °C, and 400 °C, respectively. The flow levels of nebulizing gas (N2), heating gas (air) and collision-induced dissociation gas (Ar) were 3 L/min, 10 L/min and 0.3 mL/min, respectively. The MS spectra were recorded in negative (–3––5 kV source voltage) and positive mode (+3–+4 kV source voltage) by scanning in the range of *m*/*z* 100–1900 at the collision energy of 5–40 eV. The system was operated under LabSolutions workstation software with the internal LC-MS library. The identification of compounds was done by the analysis of their retention time, ultraviolet, and mass-spectrometric data comparing the same parameters with the reference samples and/or literature data.

### 2.6. Flavonol O-Glycosides Hydrolysis and HPLC-DAD-ESI-QQQ-MS Analysis

Powdered total extract of *R. rosea* organs (100 mg) was mixed with acetone (20 mL) and hydrochloric acid (20%; 7 mL), and boiled under a reflux condenser for 20 min. The mixture was filtered through absorbent cotton into the separating funnel, mixed with water (20 mL) and extracted with diethyl ether (5 × 20 mL). The ether layer was dried by filtration over anhydrous sodium sulfate and concentrated in vaccuo until dryness. The dry residue was dissolved in methanol (5 mL), filtered through a 0.22 μm polytetrafluoroethylene (PTFE) syringe filter, and used for HPLC-DAD-ESI-QQQ-MS analysis. The general conditions were described in Section 2.5 except the gradient program used (0.0–4.0 min 15.0–22.0% B, 4.0–12.0 min 22.0–40.0% B, 12.0–17.0 min 40.0–52.0% B, 17.0–19.0 min 52.0–15.0% B, 19.0–25.0 min 15.0% B). The MS spectra were recorded in negative mode (–3 kV source voltage) by scanning in the range of *m*/*z* 80–1000 at the collision energy of 10 eV.

### 2.7. HPLC-MS Quantification

Quantification of compounds **1–146** was realized using HPLC-MS data (MS peak area) in conditions described in Section 2.5. To prepare the stock solutions of reference compounds, 44 standards (Tables S1 and S2) were accurately weighed (10 mg) and individually dissolved in methanol in a volumetric flask (10 mL). The external standard calibration curve was generated using six data points, 100, 50, 25, 10, 5, 1 µg/mL. The calibration curves were created by plotting the MS peak area vs. the concentration levels and the validation criteria (correlation coefficients, *r*^2^; standard deviation, *S*_YX_; limits of detection, LOD; limits of quantification, LOQ; linear ranges) was calculated using the previous recommendations [29] (Table S2). All the analyses were carried out in triplicate and the data were expressed as mean value ± standard deviation (SD). For the preparation of sample solution, an accurately weighted powdered plant of *R. rosea* organs (100 mg) or total extract of *R. rosea* organs (40 mg) were placed in an Eppendorf tube, 2 mL of 70% methanol was added. Then the sample was extracted twice in an ultrasonic bath for 30 min at 30 °C and centrifuged (3000× *g*, 15 min). Combined supernatants were transferred to the volumetric flask (5 mL) and the final volume was reduced to 5 mL. The resultant extract was filtered through a 0.22 μm PTFE syringe filter before injection into the HPLC system for analysis. Caffeine (final concentration 500 μg/mL in acetonitrile) and benzoic acid (final concentration 250 μg/mL in acetonitrile) were used as the internal standards for analysis of Sep-Pak C_18_ eluates as well as picein (final concentration 250 μg/mL in methanol) for polyamide eluates I, scopoletin-7-*O*-neohesperidoside (final concentration 250 μg/mL in 40% methanol) and isorhamnetin (final concentration 125 μg/mL in methanol) for polyamide eluates II, and 4-*O*-caffeoylquinic acid (final concentration 200 μg/mL in methanol) for polyamide eluates III.

### 2.8. Simulated Gastrointestinal Digestion and Gut Microbiota Incubation

The assays previously described used to simulate gastrointestinal digestion [32] and gut microbiota incubation [33]. For the simulation, the samples of *R. rosea* dry extracts (rhizomes, leaves, flowers extracts; 500 mg) were incubated with simulated gastric fluid [32] (25 mL, pH 2.0) in a shaking water bath (37 °C, 167 rpm, 60 min) followed by neutralization (1 M NaOH) up to pH 7.0 (gastric phase) and HPLC-DAD analysis (Section 2.9, conditions HPLC-DAD-1; samples were filtered through 0.22 μm syringe filters before injection into the HPLC system). To prepare simulated gastric fluid, aliquots of 61.0 mL NaCl (200.0 g/L), 11.7 mL NaH_2_PO_4_ (88.8 g/L), 35.8 mL KCl (89.6 g/L), 70.0 mL CaCl_2_·2H_2_O (22.2 g/L), 39.0 mL NH_4_Cl (30.6 g/L), and 32.5 mL HCl (37%) were mixed in a volumetric flask and the total volume was adjusted to 250 mL by distilled water. Then the solution was supplemented by HCl up to pH 2.0 (solution I). The simulated gastric fluid was prepared before use by mixing pepsin (400 mg; Sigma-Aldrich; 3200–4500 units/mg protein) with the 25 mL of solution I (stored at 4 °C).

The gastric fluid treated sample was transferred to the dialysis bag and mixed with bile solution (1 mL) and simulated intestinal fluid (4 mL) [32] and continuously stirred (4 h) in a clear simulated intestinal fluid without of pancreatin addiction (1000 mL, pH 7.0, 37 °C) (intestinal phase). Non-dilalyzed retentate after 4 h incubation used for HPLC-DAD analysis after filtration (Section 2.9, conditions HPLC-DAD-1). To prepare simulated intestinal fluid, aliquots of 75.0 mL NaCl (200.0 g/L), 75.0 mL NaHCO_3_ (84.7 g/L), 19.0 mL KH_2_PO_4_ (8 g/L), 12.0 mL KCl (89.6 g/L), and 19.0 mL MgCl_2_ (5 g/L) were mixed in a volumetric flask and the total volume was adjusted to 200 mL by distilled water (solution II). The simulated intestinal fluid was prepared before use by mixing pancreatin (40 mg; AppilChem GmbH, Darmstadt, Germany; amylase 22,500 U/g, lipase 22,500 U/g, protease 1050 U/g) with 4 mL of solution II. The simulated bile solution consisted of bile (50 mg) dissolved in 10 mL of solution contained 2.93 mL NaCl (175.3 g/L), 6.65 mL NaHCO_3_ (84.7 g/L), 0.40 mL KCl (89.6 g/L), and 0.02 mL HCl (37%).

The retentate after intestinal phase subjected to lyophilic drying and the dry residue dissolved in 5 mL of distilled water and neutralized if necessary. The resultant solution (5 mL) was mixed with fecal slurries (1 g in 5 mL of brain heart infusion) donated by healthy volunteers as described previously [33] and pure brain heart infusion (15 mL). The samples were incubated under anaerobic conditions (37 °C, 48 h) in BD GasPak^TM^ EZ anaerobe container system sachets (New Jersey, NJ, USA) then centrifuged (6000× *g*, 20 min), mixed with acetonitrile (1:1), passed through 0.22 μm syringe filters and analyzed by HPLC-DAD assay (Section 2.9, conditions HPLC-DAD-2).

### 2.9. HPLC-DAD Assays for Gastric, Intestinal, and Gut Microbiota Media

Assays of HPLC-DAD quantification were performed in microcolumn HPLC chromatograph MiLiChrom A-02 (Econova, Novosibirsk, Russia) coupled with a ProntoSIL-120-5-C18 AQ column (1 × 50 mm, ∅ 1 μm; Metrohm AG; Herisau, Switzerland) at the column temperature 30 °C. Eluent A was 0.2 M LiClO_4_ in 0.01 M HClO_4_ and eluent B was 0.01 M HClO_4_ in acetonitrile. The injection volume was 1 μL, and the elution flow was 150 μL/min. Gradient programs: conditions HPLC-DAD-1—0.0–20.0 min 5.0–100.0% B, 20.0–24.0 min 100.0% B, 24.0–27.0 min 100.0–5.0% B; HPLC-DAD-2—0.0–15.0 min 7.0–65.0% B, 15.0–22.0 min 65.0–100.0% B, 22.0–25.0 min 100.0–15.0% B. The DAD acquisition was performed at 210 nm. The system was operated under MiLiChrom workstation software. The 13 reference standards for HPLC-MS quantification (Section 2.7) were used for HPLC-DAD quantifying after calibration curve generating (Tables S1 and S3). All the analyses were carried out in triplicate and the data were expressed as mean value ± standard deviation (SD). The samples of total extracts of *R. rosea* organs treated with gastric and intestinal fluids were chromatographed without any pretreatment after filtering through a 0.22 μm PTFE syringe.

### 2.10. Incubation of R. rosea Extracts with Digestive Enzymes Mixture

The sample of *R. rosea* extract from rhizome or flowers (500 mg) was dissolved in water (25 mL), mixed with pepsin (400 mg) and pancreatin (40 mg) and incubated in a shaking water bath (37 °C, 167 rpm, 5 h). An aliquot (1 mL) of the incubated mixture was vigorously shaken with 2 mL of acetonitrile in the Eppendorf tube than filtered through a 0.22 μm PTFE syringe filter and analyzed using HPLC-DAD assay (conditions 1; Section 2.10).

### 2.11. Trolox-Equivalent Content in Simulated Gastric, Intestinal and Gut Microbiota Media

The Trolox-equivalent content in digestive media and gut microbiota medium was found using bromine radical scavenging assay based on the coulometric titration method with electrogenerated bromine radicals [31]. The measurements were carried out using Expert-006 potentiostat (Econics Expert Ltd., Moscow, Russia) with four-electrode two-compartment electrochemical cell. A bare platinum foil with 1 cm^2^ surface area was used as the working electrode, and a platinum wire separated from the anodic compartment with a semipermeable diaphragm—as the auxiliary electrode. The time of titration was used for the total antioxidant capacity calculation that was expressed in units of the quantity of electricity (Coulombs (C)) spent for titration of the full probe of digestive media. The reference compound Trolox solutions (500, 250, 100, 50, 10 μg/mL in methanol) was titrated coulometrically, and a calibration curve was plotted in coordinates “concentration (μg/mL)—the quantity of electricity (C)”. Finally, the value of Trolox-equivalent content was calculated as mg Trolox equivalents per probe. Values are expressed as mean obtained from five independent experiments. The gastric, intestinal, and gut microbiota fluids (media) showed zero or traces Trolox-equivalent content.

### 2.12. Statistical Analysis

Statistical analyses were performed using a one-way analysis of variance (ANOVA), and the significance of the mean difference was determined by Duncan’s multiple range test. Differences at *p* < 0.05 were considered statistically significant. The results are presented as mean values ± SD (standard deviations) of the three–five replicates.

## 3. Results and Discussion

### 3.1. Phenolic Composition and Antioxidant Activity of Rhodiola rosea Extracts

To better understand if there was any prospect for studies and practical application of *R. rosea* herb compared to traditional rhodiola subterranean organs, we must undertake a reassessment of our knowledge of the chemistry and bioactivity of *R. rosea* underground roots and rhizomes and aerial parts (leaves, flowers and stems). In this study, we will focus mostly on the phenolic compounds in *R. rosea* due to the greater scientific weight and a better understanding of their mode of action. Seven groups of phenolics were chosen and quantitatively analysed (Table 3) based on known *Rhodiola* plants phenolome data [2]. Non-trace levels were found for flavonoids, catechins, procyanidins, phenylpropanoids and gallotannins; ellagitannins and anthocyanins were treated as non-essential. The total phenolic content was high in all *R. rosea* organs, especially in rhizomes (140.60 mg/g), leaves (122.98 mg/g), and flowers (112.79 mg/g) followed by roots (44.87 mg/g) and stems (21.84 mg/g).

Flavonoids were at the highest level in the aerial parts, such as flowers (46.36 mg/g) and leaves (16.71 mg/g), and lower in roots and rhizomes (0.75–1.89 mg/g). The present data did not confirm the earliest records of low flavonoid content (<2 mg/g in the dry plant) in samples of *R. rosea* herb, as opposed the high amount of flavonoids in roots and rhizomes (12–19 mg/g in the dry plant) [34,35]. In the absence of systematic data about biochemical features of the Siberian populations of *R. rosea*, it is possible to talk about the regional variation of flavonoid content resulting in conflicting data, particularly as that was previously described for the European populations of *R. rosea* [36,37].

Catechins were the most significant group of phenolics in underground organs, with 10.84 and 61.30 mg/g in roots and rhizomes, respectively. Biogenetically close to catechins, procyanidins showed high contents in rhizomes (31.37 mg/g) and roots (9.21 mg/g). The concentration of catechins and procyanidins in aerial organs was not more than 2 mg/g. There is no previous comparative data of catechins and procyanidins distribution in *R. rosea* plant except the information that catechin content in the root may vary from 4.6 mg/g in Polish samples [38] to 20 mg/g in samples of Indian origin [39].

Phenylpropanoids of *R. rosea* that are mostly derivatives of cinnamyl alcohol (rosavins) and salidroside [2] were highest in rhizomes (46.45 mg/g) and roots (21.89 mg/g) and lowest in leaves (8.27 mg/g), stems (5.11 mg/g), and flowers (1.14 mg/g). In early research, rhizomes of *R. rosea* traditionally enriched by phenylpropanoids rosavins at 2.5–3.5 times more than roots [36] and aerial parts showed no detectable amounts of salidroside and rosavins [37]. The variation of phenylpropanoid content in roots may reach from nil in Russian and Chinese samples to 86 mg/g in Norwegian plants [40].

Gallotannins, quantified in *R. rosea* for the first time, were the basic phenolic group in leaves (97.53 mg/g), flowers (63.11 mg/g) and stems (12.35 mg/g). The roots and rhizomes contents of gallotannins were low (0.73–1.04 mg/g).

Already at this stage of our study, it seems clear that underground organs tend to concentrate catechins, procyanidins, and phenylpropanoids while aerial organs accumulated flavonoids and gallotannins. These differences in organ chemistry would affect the bioactivity potentials of *R. rosea* extracts.

As referred to in many papers, the antioxidant potential of *R. rosea* roots and/or rhizome extracts and remedies are already well known [2,3]. We, therefore, compared the bioactivities of five rhodiola extracts derived from roots, rhizomes, flowers, leaves and stems. The antioxidant properties of mentioned extracts were studied in five assays; the scavenging capacity against 2,2-diphenyl-1-picrylhydrazyl radical (DPPH^•^), 2,2′-azino-bis(3-ethylbenzothiazoline-6-sulfonic acid) cation radical (ABTS^•^^+^), superoxide radical (O_2_^•^^−^), hydroxyl radical (^•^OH) and carotene bleaching assay and expressed activity were found in all cases (Table 4).

The reference compound trolox showed close or less power of activity. The most active extracts were from *R. rosea* rhizomes, leaves and flowers followed by the stem and root extracts. This trend is not surprising at all, the high content of known strong antioxidants, such as catechins, procyanidins, gallotannins, and flavonoids, in *R. rosea* extracts should have led to the same results, but in fairness, the high antioxidant potency of *R. rosea* herb is shown for the first time. However, the lack of precise data about metabolites of *R. rosea* herb, as well as the quantitative content and comparative assessment of metabolic profiles of underground organs and aerial parts of the plant makes it hard to bring to a close the issue of *R. rosea* antioxidants.

### 3.2. HPLC-DAD-QQQ-ESI-MS Profiles of R. rosea Organs: Quantitative and Quantitative Study

**Figure 1:**
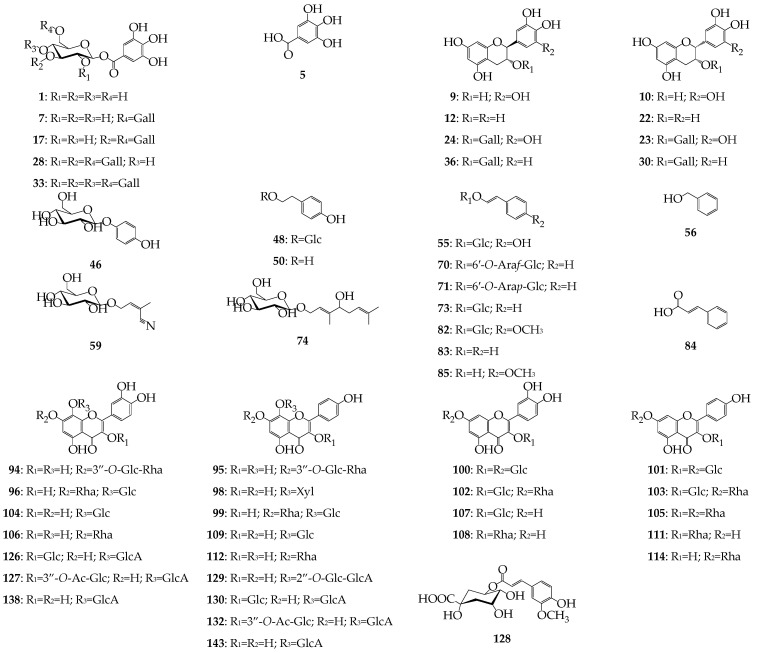
Structures of known compounds found in *Rhodiola rosea*. Abbreviation used: Ac—acetyl; Ara*f*—arabinofuranose; Ara*p*—arabinopyranose; Gall—galloyl; Glc—glucose; GlcA—glucuronic acid; Rha—rhamnose; Xyl—xylose.

The high-performance liquid chromatography with diode array and electrospray triple quadrupole mass detection (HPLC-DAD-ESI-QQQ-MS) was using in both positive and negative mode to separate *R. rosea* metabolites. The diverse compounds found in *R. rosea* organs gave unsuccessful separation using only one chromatographic run. We used preliminary solid-phase extraction of total methanolic extracts on the C18 Sep-Pak cartridges to isolate the catechin/procyanidin/gallotannins fraction [41] and polyamide cartridges to isolate simple phenolics/cinnamic glycosides, neutral/acidic flavonoids and phenylpropanoids [42,43,44,45]. This approach coupled with chromatographic, UV- and mass-spectrometric identification (Tables S4–S6) made it possible to find 146 compounds in extracts of roots, rhizomes, leaves, flowers and stems of *R. rosea* (Table 5, Figures S1–S4) and 54 compounds were detected using the reference standards (Figure 1).

#### 3.2.1. Galloyl *O*-Glycosides

Gallic acid (**5**) and twenty-one of its *O*-glucosides (**1–4, 6, 7, 13, 17, 18, 27–29, 21–35, 37–40**) were found in *R. rosea* roots/rhizomes (5 compounds), leaves (13 compounds), flowers (16 compounds) and stems (16 compounds). Galloyl *O*-glycosides have different numbers of galloyl substituents (1–8) and five compounds were identified using the reference standards, 1-*O*-galloyl glucose (**1**), 1,6-di-*O*-galloyl glucose (**7**), 1,3,6-tri-*O*-galloyl glucose (**17**), 1,2,3,6-tetra-*O*-galloyl glucose (**28**), and 1,2,3,4,6-penta-*O*-galloyl glucose (**33**).

The remaining compounds were mono-*O*-galloyl glucoses (**2**–**4**), di-*O*-galloyl glucose (**6**), tri-*O*-galloyl glucoses (**13**, **18**), tetra-*O*-galloyl glucoses (**27**, **29**, **31**), penta-*O*-galloyl glucose (**32**), hexa-*O*-galloyl glucoses (**34**, **35**), hepta-*O*-galloyl glucoses (**37**, **38**), and octa-*O*-galloyl glucoses (**39**, **40**) with similar UV profiles and specific mass-spectrometric patterns typical for gallic acid derivatives [57]. Only **5**, **28** and 1,2,6-tri-*O*-galloyl glucose were previously detected in *R. rosea* roots [46], pointing to the first discovery of twenty galloyl *O*-glycosides in *R. rosea*. Other known sources of galloyl *O*-glycosides are *R. crenulata* roots [58] and *R. sachalinensis* roots [20].

#### 3.2.2. Catechins

Eight known monomeric flavan-3-ols (catechins) was successively identified in roots and rhizomes of *R. rosea,* as well as one catechin in stems. Gallocatechin (**9**), epigallocatechin (**10**), catechin (**12**), epicatechin (**22**), epigallocatechin gallate (**23**), gallocatechin gallate (**24**), epicatechin gallate (**30**), and catechin gallate (**36**) were the components detected in underground organs and **9** was only found in stems. To date, however, five catechins (**10**, **12**, **22**, **23**, **30**) were known in *R. rosea* roots [38] but catechins were found for the first time in rhizomes and stems.

#### 3.2.3. Procyanidins

The common satellite compounds to catechins are their oligomers, procyanidins, and they were found in roots, rhizomes, flowers, and stems of *R. rosea*; only leaves did not contain procyanidins. The mass spectrometric data were typical for *n*-flavan-3-ols [47] and suggested that the fourteen procyanidins found were oligomers of epigallocatechin and/or epigallocatechin gallate including:

dimers—epigallocatechin dimer (**8**; *m*/*z* 609 [M − H]^−^→305), epigallocatechin-epigallocatechin gallate (**11**; *m*/*z* 761 [M − H]^−^→457, 305), epigallocatechin gallate dimer (**14**; *m*/*z* 913 [M − H]^−^→457);

trimers—epigallocatechin trimer (**15**; *m*/*z* 913 [M − H]^−^→609, 305), di(epigallocatechin)-epigallocatechin gallate (**16**; *m*/*z* 1065 [M − H]^−^→761, 609, 457, 305), epigallocatechin-di(epigallocatechin gallate) (**19**; *m*/*z* 1217 [M − H]^−^→913, 761, 457, 305), epigallocatechin gallate trimer (**20**; *m*/*z* 1369 [M − H]^−^→913, 457);

tetramers—tri(epigallocatechin)-epigallocatechin gallate (**21**; *m*/*z* 1369 [M − H]^−^→1065, 913, 761, 609, 457, 305), di(epigallocatechin)-di(epigallocatechin gallate) (**25**; *m*/*z* 1521 [M − H]^−^→1217, 913, 761, 457, 305), epigallocatechin-tri(epigallocatechin gallate) (**26**; *m*/*z* 1673 [M − H]^−^→1369, 1217, 913, 761, 457, 305), epigallocatechin gallate tetramer (**41**; *m*/*z* 1825 [M − H]^−^→1369, 913, 457);

pentamers—tetra(epigallocatechin)-epigallocatechin gallate (**42**; *m*/*z* 1673 [M − H]^−^→1369, 1217, 1065, 913, 761, 609, 457, 305), tri(epigallocatechin)-di(epigallocatechin gallate) (**43**; *m*/*z* 1825 [M–H]^−^→1521, 1369, 1217, 1065, 913, 761, 457, 305), di(epigallocatechin)-tri(epigallocatechin gallate) (**44**; *m*/*z* 1977 [M − H]^−^→1673, 1521, 1369, 1065, 913, 609, 457, 305).

Previous data for the procyanidin profile of *R. rosea* rhizomes harvested in Norway gave close results [59], but roots, flowers and stems of *R. rosea* were studied for the first time.

#### 3.2.4. Simple Phenolics

Hydroquinone *O*-glucoside or arbutin (**46**) and arbutin *O*-pentoside (**45**) were newly found as components of all *R. rosea* organs. The presence of **46** was demonstrated in *R. sacra* roots [60] and *R. kirilowii* roots [61]. Benzyl alcohol (**56**) and three of its *O*-glucosides, benzyl alcohol *O*-hexoside **53** and two benzyl alcohol *O*-hexoside-*O*-pentosides **51** and **52,** were also detected as simple phenolics in the whole plant. The known benzyl alcohol *O*-glucoside was isolated from the roots of *R. rosea* [62] along with benzyl alcohol *O*-(6″-*O*-arabinopyranosyl)-glucoside [63] and benzyl alcohol *O*-(6″-*O*-arabinofuranosyl)-glucoside [64].

#### 3.2.5. Phenethyl Alcohol Derivatives

Tyrosol (*p*-hydroxyphenethyl alcohol) (**50**) and its *O*-glucoside salidroside (**48**) are the most frequently reported components of rhodiola plants. Tyrosol, first prepared in 1911 [65] and later found in *R. rosea* roots [48], is still the subject of many studies from different directions [66]. In our research, both compounds **48** and **50** were identified not only in roots and rhizomes but also in the aerial part, a previously unknown source. Compound **47,** described as tyrosol *O*-hexoside-*O*-pentoside (*m*/*z* 431 [M − H]^−^→299, 137), was most likely an *O*-arabinosyl analogue of salidroside that was not yet characterized, but its *O*-methyl ester mongrhoside was found in *R. rosea* [67]. An unknown tyrosol *O*-desoxyhexoside (**49**) and three acylated derivatives of salidroside with one (**65**, **66**) and two (**67**) acetyl groups were the components in the aerial part.

#### 3.2.6. (Hydroxy)Cinnamates

Seventeen (hydroxyl)cinnamates of various structures were found in the whole *R. rosea* plant. Cinnamyl alcohol (**83**) and its typical for *R. rosea O*-glucosides, rosarin (**70**), rosavin (**71**) and rosin (**73**) were successfully identified using reference standards only in roots and rhizomes. Glucoside **72** was isomeric to **70** and **71** (*m*/*z* 427 [M − H]^−^) and its most probable structure was cinnamyl alcohol *O*-(6′-*O*-xylosyl)-glucoside, the known *R. rosea* glucoside [52]. Two triglucosides of cinnamyl alcohol **68** and **69** from the underground part have close to **70**–**72** mass spectral behaviours but were 132 a.m.u. larger, pointing to additional pentosyl residue in their structures. The *O*-pentosyl derivatives of **70**–**72** are still unknown. Triandrin (sachaliside I) or *p*-hydroxycinnamyl alcohol *O*-glucoside (**55**) was described early as a component of roots [48] but its presence in the aerial part was shown for the first time. The *O*-pentoside of *p*-hydroxycinnamyl alcohol *O*-glucoside **54** in roots and rhizomes and two monoacetates **75** and **76** from the aerial part of *R. rosea* have no close structures in known plant metabolites. The known *R. rosea* hydroxycinnamates *p*-methoxycinnamyl alcohol (**85**) and its *O*-glucoside vimalin (**82**) [52], together with two *p*-methoxycinnamyl alcohol *O*-hexoside-*O*-pentosides **80** and **81,** were discovered in roots and rhizomes. Two isomeric glucosides of *p*-methoxycinnamyl alcohol with (6″-*O*-arabinopyranosyl)-glucoside and (6″-*O*-arabinofuranosyl)-glucoside fragments were characterized in *R. rosea* previously [2,52]. Two acids, cinnamic acid (**84**) and 3-*O*-feruloylquinic acid (**128**), were first found in *R. rosea* organs. Hence, although the chemodiversity of *R. rosea* (hydroxyl)cinnamates is well studied, it is still possible to find new compounds in both roots and the herb.

#### 3.2.7. Hydroxynitrile Glucosides

Three hydroxynitrile glucosides **57**–**59** were the components of *R. rosea* organs, including the known rhodiocyanoside A (**59**) [59] identified using the reference standard. The isomeric compound **58** with close chromatographic mobility was tentatively determined as rhodiocyanoside D (**59**), also found in *R. rosea* roots [68]. Both compounds were detected primarily in *R. rosea* herb. The roots/rhizome component **57** gave a deprotonated ion with *m*/*z* 390, demonstrating the presence of extra pentosyl units in **57** or **59** skeletons. The closest known analogue is rhodiocyanoside F from *R. sacra* roots [69].

#### 3.2.8. Monoterpene *O*-Glucosides

Nine monoterpene *O*-glucosides were found in various parts of *R. rosea* and rosiridin (rosiridol 1-*O*-glucoside; **74**) identified with reference standard was discovered previously [39]. The remaining components were rosiridol *O*-glucosides, such as di-*O*-hexoside-*O*-pentoside (**60**), *O*-hexoside-di-*O*-pentosides (**61**, **62**), *O*-hexoside-*O*-pentosides (**63**, **64**), *O*-acetyl-*O*-hexoside (**77**), and di-*O*-acetyl-*O*-hexosides (**78**, **79**). Rhodioloside F {rosiridol 1-*O*-(6′-*O*-arabinopyranosyl)-glucoside} [64] can be seen as a possible candidate for **63** or **64**. Other compounds have no known analogues.

#### 3.2.9. Flavonol *O*-Glycosides

To separate the diversity of *R. rosea* flavonoids, we successfully used the polyamide solid-phase extraction technique to isolate two fractions of flavonol *O*-glycosides as neutral (with no uronide- and/or acyl-substituents) and acidic (uronide- and/or acyl-containing compounds). Finally, sixty flavonol *O*-glycosides (**86**–**146**) were found both in neutral (29 compounds; **86**–**114**) and acidic (31 compounds; **115**–**146**) flavonoid fractions. The HPLC-MS profiles also allowed concluding that the neutral flavonol *O*-glycosides were the trace components of root/rhizome and leaf extracts and dominated in the flowers extract opposite the acidic flavonol *O*-glycosides, which were the major flavonoids of root/rhizome and leaf extracts and minor for the flowers extract. It is also important to note that the previous data on *R. rosea* flavonoids refers to only the neutral flavonol *O*-glycosides [2,13,14,15,53,54,55,56,70] (Table S7), so this is the first report describing the acidic flavonol *O*-glycosides as components of *R. rosea*.

The preliminary analysis of hydrolysis products of flavonoid *O*-glycosides indicated the presence of five flavonol aglycones in detectable levels (Figure S5). Four compounds were identified using the reference standards as kaempferol, quercetin, herbacetin, and gossypetin and were the usual aglycones of *R. rosea* [13,14,15,53,54,55,56]. An additional minor flavonol with lower HPLC mobility than gossypetin was found in hydrolysates of leaf and flower extracts. The UV pattern of the minor flavonol aglycone was typical for 8-hydroxylated flavonols [13,15,57] and the compound also gave a deprotonated ion with *m*/*z* 333, showing its similarity to gossypetin with an extra-hydroxyl group, such as a hibiscetin (5′-hydroxy-gossypetin). Hibiscetin derivatives were not detected in *Rhodiola* genus previously but the systematically close genus *Sedum* contains hibiscetin in free [71] and glucosylated forms [72,73].

The features of UV spectra or spectral pattern of flavonoids were used to facilitate the identification and determine the aglycone substitution type (Tables S5 and S6). This was particularly true in the case of herbacetin and gossypetin di-, tri-, and tetra-glucosides that have many more numbers of substitutions than the kaempferol or quercetin derivatives.

Herbacetin *O*-glucosides. The largest group of flavonol *O*-glycosides found in *R. rosea* were herbacetin *O*-glucosides with twenty-four members (**87**, **92**, **95**, **98**, **99**, **109**, **110**, **112**, **113**, **119**–**122**, **129**–**132**, **135**–**137**, **143**–**146**) including nine known compounds identified by comparing with reference standards. Rhodiosin {herbacetin-7-*O*-(3″-*O*-glucosyl)-rhamnoside; **95**}, detected in leaves and flowers, was found for the first time in the roots of *R. rosea* [55], as were herbacin (herbacetin-8-*O*-glucoside; **109**) [54] and rhodionin (herbacetin-7-*O*-rhamnoside; **112**) [55]. Rhodalin (herbacetin-8-*O*-xyloside; **98**) and rhodionidin (herbacetin-7-*O*-rhamnoside-8-*O*-glucoside; **99**), the flower and leaf components [14], were also detected in roots and rhizomes. Four known acidic herbacetin *O*-glucosides, like rhodiquadrin C {herbacetin-8-*O*-(2″-*O*-acetyl)-glucuronide; **129**}, herbacetin-3-*O*-glucoside-8-*O*-glucuronide (**130**), herbacetin-3-*O*-(3″-*O*-acetyl)-glucoside-8-*O*-glucuronide (**132**) and melocorin (herbacetin-8-*O*-glucuronide; **143**), were typical for the herb of *Rhodiola quadrifida* [17] but were found in the whole *R. rosea* plant for the first time.

Four neutral *O*-glycosides (**87**, **92**, **110**, **113**) were identified as herbacetin derivatives based on their UV- and mass-spectrometric data and UV-patterns allowed to know their substitution type. Compound **87** was 7,8-di-*O*-substituted tetraglucoside (herbacetin tri-*O*-hexoside-*O*-desoxyhexoside) and **92** was 7,8-di-*O*-substituted triglucoside (herbacetin di-*O*-hexoside-*O*-desoxyhexoside); their natural analogues are unknown. However, the specificity of their mass spectra showed the non-terminal position of desoxyhexose (**87**: *m*/*z* 935→773→611→449→303; **92**: *m*/*z* 773→611→449→303) related to herbacetin-7-*O*-(3″-*O*-glucosyl)-rhamnoside (**95**: *m*/*z* 611→449→303). This suggests that **92** and **87** were relative to **92** with an additional one and two fragments of hexose (glucose) at the 8-*O*-position, or herbacetin-7-*O*-(3″-*O*-glucosyl)-rhamnoside-8-*O*-glucoside and herbacetin-7-*O*-(3″-*O*-glucosyl)-rhamnoside-8-*O*-(X’’-*O*-glucosyl)-glucoside, respectively. Two monoglucosides, **110** and **113,** were *O*-desoxyhexosides with 8-*O*- and 3-*O*-substitution, respectively. The closest analogue of **110** is herbacetin-8-*O*-rhamnoside of litvinolin from *R. litvinovii* [16] and *R. krylovii* [74], and the possible structure of **113** is herbacetin-3-*O*-rhamnoside still unknown, which is surprising since the more complex herbacetin-3-*O*-rhamnoside-8-*O*-glucoside was already isolated from the plant source [75].

Two acidic triglucosides, **119** and **120,** or herbacetin di-*O*-hexoside-*O*-hexuronides with close mass spectra have various UV patterns, identifying their substitutions as 3,8-di-*O*- and 3,7,8-tri-*O*-type, respectively. Assuming the presence of herbacetin-3-*O*-glucoside-8-*O*-glucuronide (**130**) in *R. rosea,* the **119** would be herbacetin-3-*O*-(X’’-*O*-glucosyl)-glucoside-8-*O*-glucuronide or herbacetin-3-*O*-glucoside-8-*O*-(X’’-*O*-glucosyl)-glucuronide, both unknown. Compound **120** was spectrally close to herbacetin-3,7-di-*O*-glucoside-8-*O*-glucuronide isolated from *Sedum dasyphyllum* [72,73]. Two mono-acylated derivatives of 3,7,8-tri-*O*-substituted herbacetin di-*O*-hexoside-*O*-hexuronide were malonate (**121**) and acetate esters (**122**). Only one acetyl ester with the structure of herbacetin-3-*O*-(3″-*O*-acetyl)-glucoside-7-*O*-glucoside-8-*O*-glucuronide is known, and it was found previously in *Sedum dasyphyllum* [72,73]. Three monoacetates of 3,8-di-*O*-substituted herbacetin *O*-hexoside-*O*-hexuronide (**131**, **135**, **136**) were found in addition to known herbacetin-3-*O*-(3″-*O*-acetyl)-glucoside-8-*O*-glucuronide (**132**). It was therefore likely that compounds **131**, **135** and **136** were the 2″-*O*-, 4″-*O*- and 6″-*O*-monoacetates of herbacetin-3-*O*-glucoside-8-*O*-glucuronide (**130**) still undiscovered. The same prediction used to characterize the structure of unknown glucoside **137** as di-*O*-acetate of 3,8-di-*O*-substituted herbacetin *O*-hexoside-*O*-hexuronide or herbacetin-3-*O*-(X’’,Y’’-di-*O*-acetyl)-glucoside-8-*O*-glucuronide. Di-acetylated glycosides of herbacetin were also found in *Rhodiola algida* [76,77]. One malonyl ester **144** and two acetyl esters, **145** and **146,** of 8-*O*-substituted herbacetin *O*-hexuronide were most likely herbacetin-8-*O*-(X’’-*O*-malonyl)-glucuronide and herbacetin-8-*O*-(X’’-*O*-acetyl)-glucuronides, respectively.

As a result, we see the presence of nine known herbacetin *O*-glucosides in *R. rosea* and three compounds with possible structures. Twenty herbacetin glycosides were new compounds with structures that remain to be defined. The literature indicates there are ten herbacetin *O*-glucosides previously detected in *R. rosea,* of which five {**95** [55], **98** [14], **99** [13,14], **109** [54], **112** [55]} were identified in this work. The unfound components were pentose-containing flavonols as acetylrhodalin {herbacetin-8-*O*-(2″-*O*-acetyl)-xyloside} [56], herbacetin-3-*O*-glucoside-7-*O*-arabinoside, herbacetin-3-*O*-glucoside-7-*O*-xyloside [15] and rhodalin (herbacetin-3-*O*-glucoside-8-*O*-xyloside) [13,14] and one desoxyhexose-containing flavonol, sinocrassoside C1 (herbacetin-3-*O*-glucoside-7-*O*-rhamnoside) [54] that is probably a trace compound or untypical for Siberian populations of *R. rosea*.

Gossypetin *O*-glucosides. Nineteen compounds (**88**, **91**, **93**, **94**, **96**, **104**, **106**, **116**–**118**, **126**, **127**, **133**, **134**, **138**–**142**) were described as gossypetin *O*-glucosides and seven were identified by comparison with standards as the known rhodioflavonoside {gossypetin-7-*O*-(3″-*O*-glucosyl)-rhamnoside; **94**}, rhodiolgidin (gossypetin-3-*O*-rhamnoside-8-*O*-glucoside; **96**), gossypin (gossypetin-8-*O*-glucoside; **104**), rhodiolgin (gossypetin-7-*O*-rhamnoside; **106**), gossypetin-3-*O*-glucoside-8-*O*-glucuronide (**126**), rhodiquadrin B {gossypetin-3-*O*-(3″-*O*-acetyl)-glucoside-8-*O*-glucuronide; **127**} and hibifolin (gossypetin-8-*O*-glucuronide; **138**). Compounds **96** and **106** were previously detected in flowers and leaves of *R. rosea* [13,14] and **94** was shown in roots [53].

The remaining gossypetin derivatives were the neutral *O*-glucosides (**88**, **91**, **93**) and acidic *O*-glucosides (**116**–**118**, **133**, **134**, **139**–**142**). Neutral triglucoside **88** was 7,8-di-*O*-substituted gossypetin di-*O*-hexoside-*O*-desoxyhexoside and its mass spectrum showed the non-terminal position of desoxyhexose (*m*/*z* 789→627→465→319) close to gossypetin-7-*O*-(3″-*O*-glucosyl)-rhamnoside (**94**: *m*/*z* 627→465→319). The possible structure of **88** was concluded as unknown gossypetin-7-*O*-(3″-*O*-glucosyl)-rhamnoside-8-*O*-glucoside, but this requires additional study. Compound **91** was 8-*O*-substituted gossypetin di-*O*-hexoside and preliminarily characterized as gossypetin-8-*O*-(X’’-*O*-glucosyl)-glucoside with no analogues in known flavonoids. Diglucoside **93** has the same molecular weight as **91** but alternative substitution, 3,8-di-*O*-glucoside, concluding that its structure is like an unknown gossypetin-3,8-di-*O*-glucoside.

Acidic glucoside **116** was a 7-*O*-hexosylated analogue of gossypetin-3-*O*-glucoside-8-*O*-glucuronide (**126**), very similar to gossypetin-3,7-di-*O*-glucoside-8-*O*-glucuronide found in *Sedum dasyphyllum* [72,73]. Its two monoacetylated derivatives, **117** and **118,** gave protonated ions with *m*/*z* 861 [M + H]^+^ that were 42 a.m.u. more than **116**. The most likely position of acetyl fragments is at the 3-*O*-glucose moiety due to the presence of gossypetin-3-*O*-(3″-*O*-acetyl)-glucoside-8-*O*-glucuronide (**127**) but this claim needs experimental proof. Two acetylated 3,8-di-*O*-substituted gossypetin *O*-hexoside-*O*-hexuronides, **133** and **134,** have similar spectra, as in the case of isomers of gossypetin-3-*O*-(X’’,Y’’-di-*O*-acetyl)-glucoside-8-*O*-glucuronide. Four mono-acylated derivatives of gossypetin-8-*O*-glucuronide (**138**) were malonyl esters (**139**, **140**) and acetyl esters (**141**, **142**). The literature search gave no results for possible structures close to **139**–**142**.

The results thus obtained for gossypetin *O*-glucosides demonstrated only seven identified compounds, one compound with a possible structure and twenty new flavonoids that need further study. Only five gossypetin *O*-glucosides were known in *R. rosea,* including some found in this work, **94** [53], **96** and **106** [13,14], and two *O*-pentosides, gossypetin-3-*O*-glucoside-7-*O*-arabinoside/xyloside [15].

Kaempferol *O*-glucosides. Five known kaempferol derivatives were identified by comparing with standards of kaempferol-3,7-di-*O*-glucoside (**101**), kaempferol-3-*O*-glucoside-7-*O*-rhamnoside (**103**), kaempferitrin (kaempferol-3,7-di-*O*-rhamnoside; **105**), afzelin (kaempferol-3-*O*-rhamnoside; **111**), and kaempferol-7-*O*-rhamnoside (**114**). Compounds **103**, **111**, and **114** were detected previously in *R. rosea* roots [2,54,56] and **101** was found in herb [15]. The unknown kaempferol glucosides were described as 3,7-di-*O*-substituted kaempferol tri-*O*-hexoside-*O*-desoxyhexoside (**86**), 3,7-di-*O*-substituted kaempferol di-*O*-hexoside-*O*-desoxyhexoside (**90**) and 7-*O*-substituted kaempferol *O*-hexoside-*O*-desoxyhexoside (**97**).

Quercetin *O*-glucosides. Only five quercetin *O*-glucosides were detected in *R. rosea,* among whom were four known compounds, like quercetin-3,7-di-*O*-glucoside (**100**), quercetin-3-*O*-glucoside-7-*O*-rhamnoside (**102**), isoquercitrin (quercetin-3-*O*-glucoside; **107**) and quercitrin (quercetin-3-*O*-rhamnoside; **108**). Triglucoside **89** was 3,7-di-*O*-substituted quercetin di-*O*-hexoside-*O*-desoxyhexoside. The previously noted quercetin *O*-glucosides of *R. rosea* are **108** [2] and quercetin-3-*O*-rhamnoside-7-*O*-glucoside [15].

Hibiscetin *O*-glucosides. The smallest group of *R. rosea* flavonol *O*-glucosides was derivatives of hibiscetin. Four members were characterized as 3,8-*O*-di-substituted hibiscetin di-*O*-hexoside-*O*-hexuronide (**115**), hibiscetin *O*-hexoside-*O*-hexuronide (**123**), hibiscetin *O*-malonyl-*O*-hexoside-*O*-hexuronide (**124**) and hibiscetin *O*-acetyl-*O*-hexoside-*O*-hexuronide (**125**).

Finally, sixty compounds were found in *R. rosea,* including 10 known flavonoids. Fifty compounds were described for *R. rosea* for the first time, of which about thirty members may be the new compounds that need additional study.

#### 3.2.10. Comparative Chemodiversity of Various Organs of *R. rosea*

One hundred and forty-six compounds found in *R. rosea* demonstrated various distributions in organs. The total numbers of components detected were 69 in stems, 87 in leaves, 90 in roots and rhizomes, and 100 in flowers of *R. rosea*. The maximal numbers of known discovered compounds were from nil in stems to 25 in rhizomes; therefore, the amounts of newly found components were 65 in rhizomes, 69 in roots and stems, 84 in leaves, and 95 in flowers. This was the first time that HPLC-DAD-tQ-ESI-MS profiling of *R. rosea* metabolites had taken place. In this regard, it is also relevant to bear in mind a large number of compounds with potentially new structures were identified in present work. Is it possible to talk about the organ-specific distribution of compounds in *R. rosea* organs? It is possible in part because some groups of compounds were found only in separate plant parts. Good examples are the poly-galloyl glucoses (tetra- to octa-substituted) found only in the aerial part of *R. rosea* in contrast to cinnamyl alcohol *O*-glucosides and catechins, which were the typical markers of underground organs. In other cases, the organ-specificity of plant metabolites was not evident.

The application of quantitative analysis of chromatographic data demonstrated more clear deviations in the chemical profiles of *R. rosea* organs in which about 90 compounds were quantified (Table 6, Table S8).

In the analysis process, among the metabolites in underground organs, the catechins and cinnamyl alcohols glucosides were the basic compounds of roots (10.58 and 19.89 mg/g) and rhizomes (57.40 and 55.76 mg/g) in contrast to aerial organs with zero to the low content of the mentioned groups. Marked levels of procyanidins (10.58–57.40 mg/g) and *p*-hydroxyphenethyl alcohol glucosides (1.97–20.12 mg/g) are also worthy of note for the underground organs. Typically, the levels of all compounds were higher in rhizomes, except flavonoids and galloylated glucoses with relatively more content in roots. The galloylated glucoses were found as phenolics with the highest content in *R. rosea* leaves (100.63 mg/g) and flowers (68.19 mg/g) and in both organs, poly-galloyl glucoses (penta, hexa, hepta) were predominant (12.21.02–37.79 mg/g). Flavonol glucosides were highest in flowers (55.55 mg/g) followed by leaves (26.85 mg/g) and stems (6.30 mg/g). The distribution of neutral and acidic types of flavonol glucosides in *R. rosea* herb was different with the domination of neutral compounds in flowers (42.88 mg/g vs. 12.67 mg/g) and acidic in leaves (26.85 mg/g vs. traces) and stems (4.56 mg/g vs. 1.74 mg/g). The basic flavonol derivatives were herbacetin glucosides (5.51–33.74 mg/g) and a smaller content was found for gossypetin glucosides (0.72–20.60 mg/g). Hydroquinone glucosides found in all plants showed maximal concentrations in flowers (24.04 mg/g) and leaves (16.62 mg/g) and lowest in the underground organs (4.64–8.87 mg/g). Hydroxynitrile glucosides and rosiridol glucosides were found in all organs as low to medium amount compounds.

*R. rosea* underground (roots/rhizomes) and aboveground parts (herb) chemically are two different plant sources accumulating various groups of compounds. The diversity of metabolites in both *R. rosea* parts requires a wide spectrum of bioactivities and this has worked in the case of antioxidant activity of all *R. rosea* organs. However, due to the lack of scientific information about digestive and gut microbiota transformation of the majority of compounds found in rhodiolas, we studied the influence of the digestive processes on the chemical composition of *R. rosea* extracts that allow us to find possible bioavailable compounds with potential high bioactivity.

### 3.3. Stability of Metabolites and Antioxidant Activity of Rhodiola rosea Extracts in Simulated Gastrointestinal Digestion Model

Digestion is a complex physical and chemical process can result in changes in the metabolic profiles of plant remedies. The key impacts are linked to the enzyme-dependent depolymerization of oligo- and polymers, destruction of acid- or base-instable compounds, and formation of protein complexes with various molecules. Ultimately, these benefits would lead to drastically changed concentrations of the selected components and changes in the bioactivity power. The *R. rosea* extracts were no exceptions and demonstrated variation in the content of some compounds after simulated gastrointestinal digestion (Tables S9–S12). Changes were made mainly to the content of procyanidins and catechins; rhizome extract lost *cc*. 95% and 97% of these two groups, respectively (Table 7).

A similar phenomenon was also found in leaf and flower extracts, which showed a 90.20–94.17% decrease of galloyl glucoses content. The changes observed for the hydroquinone glucoside arbutin and *p*-hydroxyphenethyl alcohol glucoside salidroside was less pronounced and cinnamyl alcohol glucosides and flavonol glucosides were highly stable.

**Figure 2:**
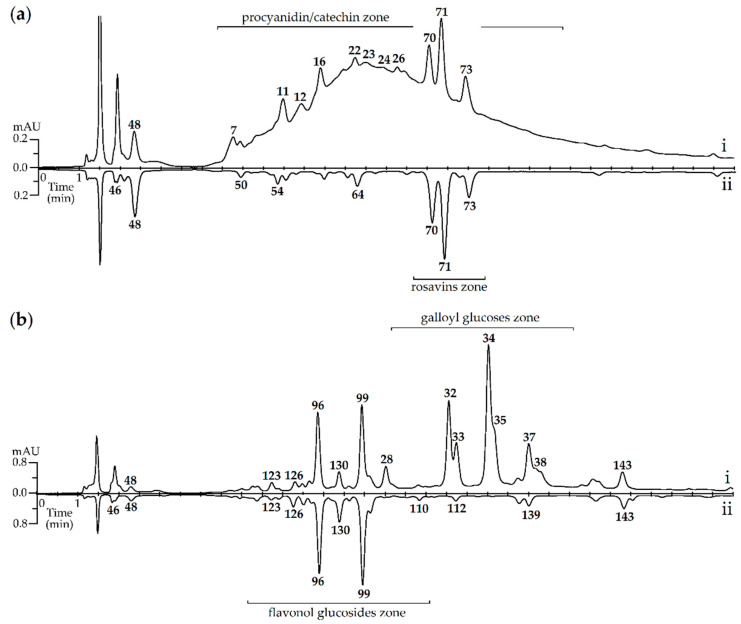
HPLC-DAD chromatograms (detector λ 210 nm) of *R. rosea* rhizome extract (**a**) and flowers extract (**b**) before (i) and after (ii) incubation with mixture of digestive enzymes (pepsin–pancreatin 10:1). Compounds: **7**—1,6-di-*O*-galloyl glucose; **11**—procyanidin dimer; **12**—catechin; **16**—procyanidin trimer; **22**—epicatechin; **23**—epigallocatechin gallate; **24**—gallocatechin gallate; **26**—procyanidin tetramer; **28**—1,2,3,6-tetra-*O*-galloyl glucose; **32**—penta-*O*-galloyl glucose; **33**—1,2,3,4,6-penta-*O*-galloyl glucose; **34/35**—hexa-*O*-galloyl glucose; **37/38**—hepta-*O*-galloyl glucose; **46**—arbutin; **48**—salidroside; **50**—tyrosol; **54**—*p*-hydroxycinnamyl alcohol *O*-hexoside-*O*-pentoside; **64**—rosiridol *O*-hexoside-*O*-pentoside; **70**—rosarin; **71**—rosavin; **73**—rosin; **96**—rhodiolgidin; **99**—rhodionidin; **110**—herbacetin *O*-desoxyhexoside; **112**—rhodionin; **123**—hibiscetin *O*-hexuronide; **126**—gossypetin-3-*O*-glucoside-8-*O*-glucuronide; **130**—herbacetin-3-*O*-glucoside-8-*O*-glucuronide; **139**—gossypetin *O*-malonyl-*O*-hexuronide; **143**—melocorin.

Consequently, digestion affected mostly the tannin-like compounds, such as procyanidins, catechins and galloyl glucoses, that are relatively resistant in digestive pH variation but can form complexes with protein molecules, such as enzymes [78]. Chromatographic experiments of model mixtures of *R. rosea* extracts with enzymes confirmed the enzyme-caused “de-tannisation” of plant matrices, demonstrating the direct impact of digestive fluids on the selected *R. rosea* compounds (Figure 2).

**Figure 3:**
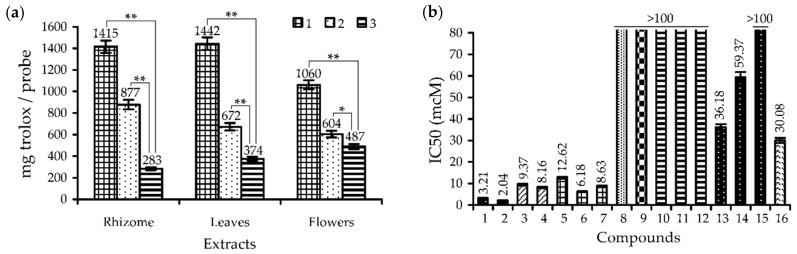
(**a**) Trolox-equivalent content in *R. rosea* extracts after in vitro treatment by the simulated gastric and intestinal media. * *p* < 0.05 vs. intestinal phase group; ** *p* < 0.001 vs. intestinal phase group. Groups: 1—initial group; 2—group after gastric phase; 3—group after intestinal phase. (**b**) Radical scavenging activity of selected compounds (as IC_50_, μM). 1—1,6-di-*O*-galloyl glucose; 2—1,2,3,4,6-penta-*O*-galloyl glucose; 3—procyanidin B1 (as an example of procyanidin dimer); 4—procyanidin C1 (as an example of procyanidin trimer); 5—epicatechin; 6—epigallocatechin gallate; 7—gallocatechin gallate; 8—arbutin; 9—salidroside; 10—rosarin; 11—rosavin; 12—rosin; 13—rhodiolgidin; 14—rhodionidin; 15—herbacetin-3-*O*-glucoside-8-*O*-glucuronide; 16—trolox.

In the face of a strong decline of phenolics in rhodiola extracts after gastrointestinal reactions, it would be logical to expect a reduction of bioactivity power. This emerges from a study on the antioxidant capacity of the extract after the gastric and intestinal stage of digestion, demonstrating the gradual decrease in trolox-equivalent content in reaction mixtures (Figure 3a). The rhizome extract showed antioxidant capacity level *cc.* 38% less after gastric phase than in the treated sample and the final greatest reduction at 80%. A close tendency was found for the leaf extract, with 53 and 74% activity decreases. The highest residual activity, at *cc.* 46% of the original level, was from the flower extract, indicating it had the best antioxidant potency. Obvious reasons for a dramatic fall of rhizome extract activity include the low residual content of antioxidants and the low activity of the residual compounds after two phases of gastrointestinal digestion. Most catechins and procyanidins, the basic radical scavengers in *R. rosea* rhizomes, were protein-bonded; in contrast, the hydroquinone glucosides (arbutin), *p*-hydroxyphenethyl alcohol glucosides (salidroside) and cinnamyl alcohol glucosides (rosavins) showed good concentrations but low activity (Figure 3b). In the case of flower extract, the residual level of the galloyl glucoses, as the strongest antioxidants of *R. rosea* herb, was extra low; however, there was the high content of herbacetin and gossypetin glucosides (rhodiolgidin, rhodionidin) that possessed good antioxidant activity (Figure 3b). The results thus obtained argued that the dynamic character of the mechanism of antioxidant protection of the *R. rosea* extracts changes in time and depending on the digestion phase.

### 3.4. Gut Microbiota Metabolites of R. rosea Extracts and Their Antioxidant Activity

**Figure 4:**
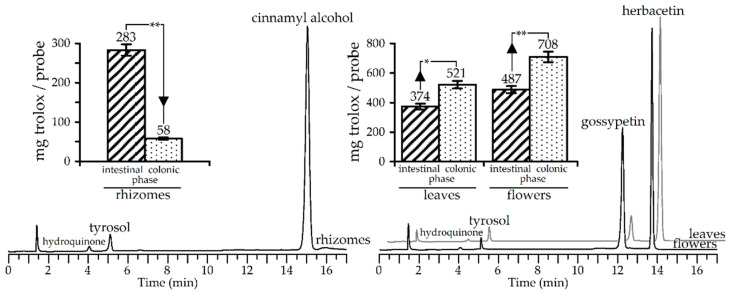
HPLC-DAD profiles of *R. rosea* extracts (rhizomes, leaves, flowers) after 24 h incubation with gut microbiota (chromatograms; detector λ 210 nm) and trolox-equivalent content in the incubation media (bars; intestinal and colonic phase data showed as comparision). * *p* < 0.05 vs. colonic phase group; ** *p* < 0.001 vs. colonic phase group.

The colonic phase of digestion, as a final step of gastrointestinal transformation, involves gut microbiota, leading to the formation of metabolites absent in the original metabolome. The extracts of *R. rosea* were not exceptions and 24 h incubation of intestinal phase samples with gut bacteria from healthy volunteers induced strong changes in HPLC patterns. In all extracts studied, the simplification of metabolic profiles or aglyconation was observed, notably cinnamyl alcohol, tyrosol, and hydroquinone were detected in gut-treated rhizome extract but leaf and flower extracts gave herbacetin, gossypetin, tyrosol, and hydroquinone (Figure 4).

These results were attributable to intensive hydrolytic reactions in the gut microbiome resulting in the formation of aglycones from glycosidic compounds. The major compound in rhizome extract, cinnamyl alcohol, was a breakdown product of rosarin, rosavin, and rosin, as well as tyrosol and hydroquinone, which originated from salidroside and arbutin, respectively. Herbacetin and gossypetin were detected in leaf and flower extracts for the same reason. The aglyconation of glucosides during fermentation with human faecal flora was previously postulated as a primary mechanism of flavonoid, salidroside, and other compound utilization [79,80,81].

Distinct changes in the chemical composition of *R. rosea* extracts have had an impact on the antioxidant activity, as demonstrated in trolox-equivalents content variation in gut microbiota medium. The *R. rosea* rhizome extract lost a great amount of its antioxidant potential, showing only 20% of its intestine phase level, which accounted for 4% of the original value.

The most obvious reason for the disastrous decline in the bioactivity of rhizome extract is a low index of antioxidant protection for the cinnamyl alcohol (IC_50_ > 200 μM) and tyrosol (IC_50_ > 200 μM). A radically different situation was found for leaf and flower extracts, which already have good antioxidant potential parameters that rise after incubation with the gut microbiome. The difference values of activity between intestinal and colonic phase levels were 40% (leaf extract) and 45% (flower extract), in favour of the latter. The gut-induced deglucosylation of *R. rosea* flavonol glucosides resulted in the removal of substituents from positions C-3, C-7, and C-8 and the formation of the more active aglycones herbacetin (IC_50_ = 28.63 ± 0.56 μM) and gossypetin (IC_50_ = 18.45 ± 0.35 μM) with free hydroxy-function. Thus, the colonic phase of digestion can influence the bioactivity of *R. rosea* extracts in different ways, depending on the original chemical profiles.

The tendency of plant phenolics to reduce content after incubation with simulated gastrointestinal fluids was demonstrated for gallotannins, catechins, procyanidins, and flavonol glycosides in several works. In early study, the members of gallotannins (pentagalloyl glucose) and catechins (epigallocatechin gallate) showed pH instability, self-association, and complex formation with digestive enzymes resulted to the lowering effect and poor recovery after digestion end [82]. Mono-, di-, and trigalloyl glucoses found in Terminalia and Emblica gave 3–16% degradation after the intestine phase of digestion [32]. The same instability was showed for the tetra- and pentagalloyl glucoses of *Myrciaria trunciflora* during in vitro gastrointestinal digestion [83]. Some catechins (epigallocatechin gallate, epigallocatechin, epicatechin gallate) significantly degraded in in vitro digestion fluids and able to form homo- and heterocatechin dimers due to autoxidation [84]. Non-galloylated catechins as catechin and epicatechin were comparatively stable. Wine procyanidins react with salivary proteins that influenced negatively on the concentration of phenolics [85], and, also, the procyanidins may significantly degrade after pancreatic digestion to give low-molecular-weight compounds [86]. In the vast majority of cases, all parameters of antioxidant protection decline rapidly after the intestinal phase of simulation since a reduction of bioavailable antioxidants content in digestive fluids. Unlike previous compounds, the flavonol glycosides of green tea [87] or Ginkgo biloba [88] demonstrated good stability up to the colonic phase of digestion and can keep the good level of antioxidativity. The final stage of digestion realized in colonic conditions leading to the destruction of most gastrointestinally stable compounds. The flavonoid glycosides are not exceptional and usually gave corresponding aglycones as in the case of cosmosiin [89], baicalin [90], rutin, hesperidin, and naringin [79]. Considering the fact that flavonoid aglycones are the better antioxidants than the glycosides [91], the antioxidant potential of colonic fluid is expected to increase. Rhodiola rosea extracts demonstrating the close mechanism of chemical transformation and antioxidant activity variation during gastrointestinal and colonic digestion. This highlights the general path of digestive utilization of plant phenolic mixtures.

## 4. Conclusions

In this paper, the whole *Rhodiola rosea* plant was shown as a natural accumulator of diverse metabolites of phenolic and non-phenolic nature, not only in roots or rhizomes, but also in leaves, flowers and stems with an unexpectedly high content of galloyl glucoses, flavonol glycosides, and other compounds in the herbal parts of the plant. These rich phenolomes of roots/rhizomes and herb made it possible to express values of antioxidant activity and identify the aerial organs of *R. rosea* as a new source of antioxidants. In an attempt to understand the evolution of *R. rosea* metabolites and bioactivity during the digestion process, in simulated conditions, we found the decreasing tannin content in rhizome, leaves, and flower extracts caused by digestive enzyme precipitation and consequently, the decrease of antioxidant capacity. The further gut microbiota assisted in transforming of *R. rosea* glucosides generated aglycones that eventually lowered the antioxidant capacity of rhizome extract but increased the bioactivity of leaf and flower extracts. Thus, the chemical features of *R. rosea* extracts have various impacts on the bioactivity due to the different methods of digestive utilization. If asked about preferences between *R. rosea* roots or herb as good antioxidants, it should be noted that the herb extracts keep antioxidant properties longer during digestion, but additional data is needed to close the issue. This study demonstrated the good biomedical prospects of *R. rosea* herb as a future plant remedy or a source of new functional products.

## Figures and Tables

**Table 1:** Number of articles (per year) focused on the chemical analysis, compound isolation, and bioactivity of *Rhodiola rosea* roots/rhizomes (R) and herb (H) ^a^.

Year	Papers		Year	Papers		Year	Papers		Year	Papers	
	R	H		R	H		R	H		R	H
1966	1	0	1987	2	0	1999	2	0	2011	26	0
1967	1	0	1988	1	0	2000	7	0	2012	31	0
1968	1	0	1989	3	0	2001	2	0	2013	53	0
1973	1	0	1990	1	0	2002	7	0	2014	40	0
1977	1	0	1991	7	0	2003	13	0	2015	42	0
1980	1	0	1992	1	0	2004	11	1	2016	50	0
1981	3	0	1993	3	0	2005	11	0	2017	35	0
1982	1	0	1994	1	0	2006	18	0	2018	47	0
1983	2	0	1995	1	0	2007	23	0	2019	69	0
1984	1	1	1996	2	0	2008	25	0	Total	626	3
1985	1	1	1997	7	0	2009	40	0			
1986	2	0	1998	3	0	2010	26	0			

^a^ The data was found in Scopus^®^ and Web of Science^®^ databases.

**Table 2:** Detailed information of *Rhodiola rosea* samples.

Organ	Collection Place	Collection Date	Coordinates	Height (m a.s.l.)	Voucher Specimens No
Herbal: leaves, flowers, stems	Chulman, Aldanskii Ulus, Sakha (Yakutia) Republic	25.VII.2019	57°00′37″ N, 124°49′02″ E	960	YA/CRA-0719/22-106
Subterranean: roots, rhizomes	Chulman, Aldanskii Ulus, Sakha (Yakutia) Republic	02.IX.2019	57°00′37″ N, 124°49′02″ E	960	YA/CRA-0919/38-471

**Table 3:** Content of phenolic compounds in *Rhodiola rosea* organs, mg/g ^a^ ± S.D.

Organ	Flavonoids	Catechins	Procyanidins	Phenylpropanoids	Gallotannins	Total Phenolics
Roots	1.89 ± 0.03	10.84 ± 0.80	9.21 ± 0.39	21.89 ± 0.43	1.04 ± 0.02	44.87
Rhizomes	0.75 ± 0.02	61.30 ± 1.22	31.37 ± 0.61	46.45 ± 0.92	0.73 ± 0.02	140.60
Flowers	46.36 ± 0.95	0.66 ± 0.02	1.52 ± 0.11	1.14 ± 0.02	63.11 ± 1.26	112.79
Leaves	16.71 ± 0.31	0.30 ± 0.01	0.17 ± 0.00	8.27 ± 0.16	97.53 ± 2.02	122.98
Stems	2.96 ± 0.05	0.32 ± 0.01	1.10 ± 0.02	5.11 ± 0.10	12.35 ± 0.25	21.84

^a^ dry plant weight.

**Table 4:** Antioxidant activity of *R. rosea* extracts.

Extracts	DPPH^• a,b^	ABTS^•^^+ b,c^	O_2_^•−^^b,d^	^•^OH ^b,e^	CBA ^b,f^
Roots	11.71 ± 0.23 **	5.37 ± 0.11 **	45.11 ± 0.90 **	37.58 ± 0.75 **	87.50 ± 2.62 **
Rhizomes	2.96 ± 0.06 **	0.62 ± 0.01 **	8.63 ± 0.17 **	6.02 ± 0.11 **	44.79 ± 1.41 **
Flowers	3.95 ± 0.08 **	0.98 ± 0.02 **	23.19 ± 0.45 **	12.83 ± 0.25 *	25.06 ± 0.63 *
Leaves	2.91 ± 0.06 **	0.53 ± 0.01 **	10.38 ± 0.21 **	7.39 ± 0.14 **	11.86 ± 0.23 *
Stems	8.20 ± 0.16	2.98 ± 0.05 *	37.69 ± 0.75 **	21.18 ± 0.42 **	37.15 ± 1.11 **
Trolox ^g^	8.38 ± 0.17	3.18 ± 0.06	125.11 ± 2.50	14.06 ± 0.28	21.05 ± 0.62

^a^ DPPH^•^—2,2-diphenyl-1-picrylhydrazyl radical scavenging capacity. ^b^ IC_50_, μg/mL ± S.D. ^c^ ABTS^•^^+^—2,2′-azino-bis(3-ethylbenzothiazoline-6-sulfonic acid) cation radical scavenging capacity. ^d^ O_2_^•−^—superoxide radical scavenging capacity. ^e •^OH—hydroxyl radical scavenging capacity. ^f^ CBA—carotene bleaching assay. ^g^ Reference compound. * *p* < 0.05 vs. Trolox group; ** *p* < 0.001 vs. Trolox group.

**Table 5:** Chromatographic (*t*_R_), mass-spectrometric data (ESI-MS) and presence in organs of compounds **1**–**146** found in *R. rosea*.

No.	*t*_R_, min ^a^	ESI-MS, *m*/*z* ^b^	Group ^c^	Compound ^d^	Presence (+) in Organs, mg/g ± S.D. ^e^				
					Roots	Rhizome	Leaves	Flowers	Stems
1	1.91 ^i^	331 ^N^	GT	1-*O*-Galloyl glucose ^S^	+	+	+	+	+
2	2.18 ^i^	331 ^N^	GT	*O*-Galloyl glucose ^L^	+	+	+	+	+
3	2.33 ^i^	331 ^N^	GT	*O*-Galloyl glucose ^L^			+		+
4	2.57 ^i^	331 ^N^	GT	*O*-Galloyl glucose ^L^				+	+
5	2.94 ^i^	169 ^N^	GT	Gallic acid ^S^	[46]	[46]	+	+	+
6	4.61 ^i^	483 ^N^	GT	Di-*O*-galloyl glucose ^L^	+				
7	4.78 ^i^	483 ^N^	GT	1,6-Di-*O*-galloyl glucose ^S^	+	+	+	+	+
8	5.11 ^i^	609 ^N^	PC	Procyanidin dimer (EGC-EGC) ^L^	+	+		+	+
9	5.27 ^i^	305 ^N^	CT	Gallocatechin ^S^	+	+			+
10	5.52 ^i^	305 ^N^	CT	Epigallocatechin ^S^	[38]	+			
11	5.76 ^i^	761 ^N^	PC	Procyanidin dimer (EGC-EGCG) ^L^	+	[47]		+	
12	5.85 ^i^	289 ^N^	CT	Catechin ^S^	[38]	+			
13	6.27 ^i^	635 ^N^	GT	Tri-*O*-galloyl glucose ^L^			+		+
14	6.31 ^i^	913 ^N^	PC	Procyanidin dimer (EGCG-EGCG) ^L^	+	[47]			
15	6.35 ^i^	913 ^N^	PC	Procyanidin trimer (EGC-EGC-EGC) ^L^	+	+			
16	6.48 ^i^	1065 ^N^	PC	Procyanidin trimer (EGC-EGC-EGCG) ^L^	+	[47]			
17	6.54 ^i^	635 ^N^	GT	1,3,6-Tri-*O*-galloyl glucose ^S^			+		+
18	6.78 ^i^	635 ^N^	GT	Tri-*O*-galloyl glucose ^L^	+	+			+
19	6.82 ^i^	1217 ^N^	PC	Procyanidin trimer (EGC-EGCG-EGCG) ^L^	+	[47]			
20	6.89 ^i^	1369 ^N^	PC	Procyanidin trimer (EGCG-EGCG-EGCG) ^L^	+	[47]			
21	6.92 ^i^	1369 ^N^	PC	Procyanidin tetramer (EGC-EGC-EGC-EGCG) ^L^	+	+			
22	7.02 ^i^	289 ^N^	CT	Epicatechin ^S^	[38]	+			
23	7.12 ^i^	457 ^N^	CT	Epigallocatechin gallate ^S^	[38]	+			
24	7.22 ^i^	457 ^N^	CT	Gallocatechin gallate ^S^	+	+			
25	7.41 ^i^	1521 ^N^	PC	Procyanidin tetramer (EGC-EGC-EGCG-EGCG) ^L^	+	[47]			
26	7.53 ^i^	1673 ^N^	PC	Procyanidin tetramer (EGC-EGCG-EGCG-EGCG) ^L^	+	[47]			
27	7.55 ^i^	787 ^N^	GT	Tetra-*O*-galloyl glucose ^L^				+	
28	7.63 ^i^	787 ^N^	GT	1,2,3,6-Tetra-*O*-galloyl glucose ^S^			+	+	+
29	7.83 ^i^	787 ^N^	GT	Tetra-*O*-galloyl glucose ^L^				+	
30	8.07 ^i^	441 ^N^	CT	Epicatechin gallate ^S^	[38]	+			
31	8.14 ^i^	787 ^N^	GT	Tetra-*O*-galloyl glucose ^L^				+	
32	8.26 ^i^	939 ^N^	GT	Penta-*O*-galloyl glucose ^L^			+	+	+
33	8.42 ^i^	939 ^N^	GT	1,2,3,4,6-Penta-*O*-galloyl glucose ^S^			+	+	+
34	8.63 ^i^	1091 ^N^	GT	Hexa-*O*-galloyl glucose ^L^			+	+	+
35	8.72 ^i^	1091 ^N^	GT	Hexa-*O*-galloyl glucose ^L^			+	+	+
36	8.92 ^i^	441 ^N^	CT	Catechin gallate ^S^	+	+			
37	8.98 ^i^	1243 ^N^	GT	Hepta-*O*-galloyl glucose ^L^			+	+	+
38	9.14 ^i^	1243 ^N^	GT	Hepta-*O*-galloyl glucose ^L^			+	+	+
39	9.63 ^i^	1395 ^N^	GT	Octa-*O*-galloyl glucose ^L^				+	
40	9.87 ^i^	1395 ^N^	GT	Octa-*O*-galloyl glucose ^L^				+	+
41	10.04 ^i^	1825 ^N^	PC	Procyanidin tetramer (EGCG-EGCG-EGCG-EGCG) ^L^	+	[47]		+	+
42	10.41 ^i^	1673 ^N^	PC	Procyanidin pentamer (EGC-EGC-EGC-EGC-EGCG) ^L^	+	+		+	+
43	11.18 ^i^	1825 ^N^	PC	Procyanidin pentamer (EGC-EGC-EGC-EGCG-EGCG) ^L^	+	+		+	
44	11.43 ^i^	1977 ^N^	PC	Procyanidin pentamer (EGC-EGC-EGCG-EGCG-EGCG) ^L^	+	[47]		+	
45	1.90 ^ii^	403 ^N^	PG	Hydroquinone *O*-Hex-*O*-Pent ^L^	+	+	+	+	+
46	1.98 ^ii^	271 ^N^	PG	Hydroquinone *O*-Glc (=arbutin) ^S^	+	+	+	+	+
47	2.09 ^ii^	431 ^N^	PE	Tyrosol *O*-Hex-*O*-Pent ^L^	+	+	+	+	+
48	2.37 ^ii^	299 ^N^	PE	Tyrosol *O*-Glc (=salidroside) ^S^	[48]	[48]	+	+	+
49	4.59 ^ii^	283 ^N^	PE	Tyrosol *O*-dHex ^L^			+	+	+
50	4.95 ^ii^	137 ^N^	PE	Tyrosol (=*p*-hydroxyphenethyl alcohol) ^S^	[48]	[48]	+	+	+
51	5.11 ^ii^	401 ^N^	PG	Benzyl alcohol *O*-Hex-*O*-Pent ^L^	+	+	+	+	+
52	5.23 ^ii^	401 ^N^	PG	Benzyl alcohol *O*-Hex-*O*-Pent ^L^	+	+	+	+	+
53	5.56 ^ii^	269 ^N^	PG	Benzyl alcohol *O*-Hex ^L^	+	+	+	+	+
54	5.82 ^ii^	443 ^N^	HC	*p*-Hydroxycinnamyl alcohol *O*-Hex-*O*-Pent ^L^	+	+			
55	6.02 ^ii^	311 ^N^	HC	*p*-Hydroxycinnamyl alcohol *O*-Glc (=triandrin) ^S^	[49]	[49]	+	+	+
56	6.27 ^ii^	107 ^N^	PG	Benzyl alcohol ^S^	+	+	+	+	+
57	6.55 ^ii^	390 ^N^	HNG	Rhodiocyanoside A/D *O*-Pent ^L^	+	+			
58	6.76 ^ii^	258 ^N^	HNG	Rhodiocyanoside D ^L^	+	+	+	+	+
59	6.97 ^ii^	258 ^N^	HNG	Rhodiocyanoside A ^S^	+	+	+	+	+
60	7.18 ^ii^	625 ^N^	TG	Rosiridol di-*O*-Hex-*O*-Pent ^L^			+	+	+
61	7.27 ^ii^	595 ^N^	TG	Rosiridol *O*-Hex-di-*O*-Pent ^L^			+	+	+
62	7.47 ^ii^	595 ^N^	TG	Rosiridol *O*-Hex-di-*O*-Pent ^L^					
63	7.52 ^ii^	463 ^N^	TG	Rosiridol *O*-Hex-*O*-Pent ^L^	+	+	+	+	+
64	7.81 ^ii^	463 ^N^	TG	Rosiridol *O*-Hex-*O*-Pent ^L^	+	+	+	+	+
65	8.18 ^ii^	341 ^N^	PE	Tyrosol *O*-Hex-*O*-Ac ^L^	+	+	+	+	+
66	8.72 ^ii^	341 ^N^	PE	Tyrosol *O*-Hex-*O*-Ac ^L^			+	+	+
67	8.98 ^ii^	383 ^N^	PE	Tyrosol *O*-Hex-di-*O*-Ac ^L^			+	+	+
68	9.02 ^ii^	559 ^N^	HC	Cinnamyl alcohol *O*-Hex-di-*O*-Pent ^L^	+	+			
69	9.26 ^ii^	559 ^N^	HC	Cinnamyl alcohol *O*-Hex-di-*O*-Pent ^L^	+	+			
70	9.57 ^ii^	427 ^N^	HC	Cinnamyl alcohol *O*-(6′-*O*-Ara*f*)-Glc (=rosarin) ^S^	[50]	[50]			
71	9.95 ^ii^	427 ^N^	HC	Cinnamyl alcohol *O*-(6′-*O*-Ara*p*)-Glc (=rosavin) ^S^	[50]	[50]			
72	10.26 ^ii^	427 ^N^	HC	Cinnamyl alcohol *O*-Hex-*O*-Pent ^L^	+	+			
73	10.51 ^ii^	295 ^N^	HC	Cinnamyl alcohol *O*-Glc (=rosin) ^S^	[50]	[50]			
74	10.98 ^ii^	331 ^N^	TG	Rosiridol 1-*O*-Glc (=rosiridin) ^S^	[51]	[51]	+	+	+
75	11.27 ^ii^	353 ^N^	HC	*p*-Hydroxycinnamyl alcohol *O*-Hex-*O*-Ac ^L^			+	+	+
76	11.67 ^ii^	353 ^N^	HC	*p*-Hydroxycinnamyl alcohol *O*-Hex *O*-Ac ^L^			+	+	+
77	12.14 ^ii^	373 ^N^	TG	Rosiridol *O*-Hex-*O*-Ac ^L^			+	+	+
78	12.48 ^ii^	415 ^N^	TG	Rosiridol *O*-Hex-di-*O*-Ac ^L^			+	+	+
79	12.81 ^ii^	415 ^N^	TG	Rosiridol *O*-Hex-di-*O*-Ac ^L^			+	+	+
80	13.65 ^ii^	457 ^N^	HC	*p*-Methoxycinnamyl alcohol *O*-Hex-*O*-Pent ^L^	+	+			
81	13.94 ^ii^	457 ^N^	HC	*p*-Methoxycinnamyl alcohol *O*-Hex-*O*-Pent ^L^	+	+			
82	14.57 ^ii^	325 ^N^	HC	*p*-Methoxycinnamyl alcohol *O*-Glc (=vimalin) ^S^	[52]	[52]			
83	15.06 ^ii^	133 ^N^	HC	Cinnamyl alcohol ^S^	[50]	[50]			
84	15.47 ^ii^	147 ^N^	HC	Cinnamic acid ^S^	+	+			
85	16.58 ^ii^	163 ^N^	HC	*p*-Methoxycinnamyl alcohol ^S^	+	+			
86	5.02 ^iii^	919 ^P^	NFG	Kaempferol tri-*O*-Hex-*O*-dHex (S37) ^L^				+	
87	5.41 ^iii^	935 ^P^	NFG	Herbacetin tri-*O*-Hex-*O*-dHex (S78) ^L^			+	+	
88	5.56 ^iii^	789 ^P^	NFG	Gossypetin di-*O*-Hex-*O*-dHex (S78) ^L^			+	+	
89	5.63 ^iii^	773 ^P^	NFG	Quercetin di-*O*-Hex-*O*-dHex (S37) ^L^			+	+	
90	5.97 ^iii^	757 ^P^	NFG	Kaempferol di-*O*-Hex-*O*-dHex (S37) ^L^	+	+	+	+	
91	6.18 ^iii^	643 ^P^	NFG	Gossypetin di-*O*-Hex (S8) ^L^				+	
92	6.21 ^iii^	773 ^P^	NFG	Herbacetin di-*O*-Hex-*O*-dHex (S78) ^L^			+	+	
93	6.32 ^iii^	643 ^P^	NFG	Gossypetin di-*O*-Hex (S38) ^L^				+	
94	6.43 ^iii^	627 ^P^	NFG	Gossypetin 7-*O*-(3″-*O*-Glc)-Rha (=rhodioflavonoside) ^S^	[53]	[53]	+	+	
95	6.63 ^iii^	611 ^P^	NFG	Herbacetin 7-*O*-(3″-*O*-Glc)-Rha (=rhodiosin) ^S^			+	+	
96	6.82 ^iii^	627 ^P^	NFG	Gossypetin 7-*O*-Rha-8-*O*-Glc (=rhodiolgidin) ^S^			[13]	[13]	+
97	7.02 ^iii^	595 ^P^	NFG	Kaempferol *O*-Hex-*O*-dHex (S7) ^L^			+	+	
98	7.21 ^iii^	435 ^P^	NFG	Herbacetin-8-*O*-Xyl (=rhodalin) ^S^	+	+	[14]	[14]	
99	7.35 ^iii^	611 ^P^	NFG	Herbacetin 7-*O*-Rha-8-*O*-Glc (=rhodionidin) ^S^	+	+	[14]	[14]	+
100	7.43 ^iii^	627 ^P^	NFG	Quercetin 3,7-di-*O*-Glc ^S^				+	
101	7.54 ^iii^	611 ^P^	NFG	Kaempferol 3,7-di-*O*-Glc ^S^	+	+		[15]	
102	7.75 ^iii^	611 ^P^	NFG	Quercetin 3-*O*-Glc-7-*O*-Rha ^S^			+	+	
103	7.97 ^iii^	595 ^P^	NFG	Kaempferol 3-*O*-Glc-7-*O*-Rha ^S^	[54]	[54]	+	+	
104	8.01 ^iii^	481 ^P^	NFG	Gossypetin 8-*O*-Glc (=gossypin) ^S^				+	
105	8.11 ^iii^	579 ^P^	NFG	Kaempferol 3,7-di-*O*-Rha (=kaempferitrin) ^S^				+	
106	8.41 ^iii^	465 ^P^	NFG	Gossypetin 7-*O*-Rha (=rhodiolgin) ^S^				[13]	
107	8.57 ^iii^	465 ^P^	NFG	Quercetin 3-*O*-Glc (=isoquercitrin) ^S^	+	+	+	+	
108	8.98 ^iii^	449 ^P^	NFG	Quercetin 3-*O*-Rha (=quercitrin) ^S^	[2]	[2]		+	
109	9.22 ^iii^	465 ^P^	NFG	Herbacetin 8-*O*-Glc (=herbacin) ^S^	[54]	[54]	+	+	
110	9.43 ^iii^	449 ^P^	NFG	Herbacetin *O*-dHex (S8) ^L^	+	+		+	
111	10.12 ^iii^	433 ^P^	NFG	Kaempferol 3-*O*-Rha (=afzelin) ^S^	+	+		+	
112	10.46 ^iii^	449 ^P^	NFG	Herbacetin 7-*O*-Rha (=rhodionin) ^S^	[55]	[55]		+	
113	11.27 ^iii^	449 ^P^	NFG	Herbacetin *O*-dHex (S3) ^L^				+	
114	11.45 ^iii^	433 ^P^	NFG	Kaempferol 7-*O*-Rha ^S^	[56]	[56]		+	
115	1.97 ^iv^	835 ^P^	AFG	Hibiscetin di-*O*-Hex-*O*-HexA (S38) ^L^			+	+	
116	2.58 ^iv^	819 ^P^	AFG	Gossypetin di-*O*-Hex-*O*-HexA (S378) ^L^			+	+	
117	2.82 ^iv^	861 ^P^	AFG	Gossypetin *O*-Ac-di-*O*-Hex-*O*-HexA (S378) ^L^			+		+
118	3.15 ^iv^	861 ^P^	AFG	Gossypetin *O*-Ac-di-*O*-Hex-*O*-HexA (S378) ^L^			+	+	
119	4.42 ^iv^	803 ^P^	AFG	Herbacetin di-*O*-Hex-*O*-HexA (S38) ^L^			+	+	
120	4.63 ^iv^	803 ^P^	AFG	Herbacetin di-*O*-Hex-*O*-HexA (S378) ^L^	+	+	+		+
121	5.02 ^iv^	889 ^P^	AFG	Herbacetin *O*-Mal-di-*O*-Hex-*O*-HexA (S378) ^L^	+	+	+	+	+
122	5.27 ^iv^	845 ^P^	AFG	Herbacetin *O*-Ac-di-*O*-Hex-*O*-HexA (S378) ^L^			+		+
123	5.52 ^iv^	673 ^P^	AFG	Hibiscetin *O*-Hex-*O*-HexA (S38) ^L^	+	+	+	+	+
124	6.03 ^iv^	759 ^P^	AFG	Hibiscetin *O*-Mal-*O*-Hex-*O*-HexA (S38) ^L^			+	+	+
125	6.18 ^iv^	715 ^P^	AFG	Hibiscetin *O*-Ac-*O*-Hex-*O*-HexA (S38) ^L^			+		+
126	6.53 ^iv^	657 ^P^	AFG	Gossypetin 3-*O*-Glc-8-*O*-GlcA ^S^	+	+	+	+	+
127	6.97 ^iv^	699 ^P^	AFG	Gossypetin 3-*O*-(3″-*O*-Ac)-Glc-8-*O*-GlcA (=rhodiquadrin B) ^S^	+	+	+	+	+
128	7.27 ^iv^	367 ^N^	HC	3-*O*-Feruloylquinic acid ^S^	+	+	+	+	+
129	7.51 ^iv^	641 ^P^	AFG	Herbacetin 8-*O*-(2″-*O*-Glc)-GlcA (=rhodiquadrin C) ^S^	+	+	+	+	+
130	7.62 ^iv^	641 ^P^	AFG	Herbacetin 3-*O*-Glc-8-*O*-GlcA ^S^	+	+	+	+	+
131	7.90 ^iv^	683 ^P^	AFG	Herbacetin *O*-Ac-*O*-Hex-*O*-HexA (S3,8) ^L^			+		
132	8.23 ^iv^	683 ^P^	AFG	Herbacetin 3-*O*-(3″-*O*-Ac)-Glc-8-*O*-GlcA ^S^	+	+	+	+	+
133	8.48 ^iv^	741 ^P^	AFG	Gossypetin di-*O*-Ac-*O*-Hex-*O*-HexA (S3,8) ^L^	+	+	+		+
134	8.73 ^iv^	741 ^P^	AFG	Gossypetin di-*O*-Ac-*O*-Hex-*O*-HexA (S3,8) ^L^	+	+	+	+	+
135	8.98 ^iv^	683 ^P^	AFG	Herbacetin *O*-Ac-*O*-Hex-*O*-HexA (S3,8) ^L^	+	+	+	+	+
136	9.49 ^iv^	683 ^P^	AFG	Herbacetin *O*-Ac-*O*-Hex-*O*-HexA (S3,8) ^L^	+	+	+	+	+
137	10.47 ^iv^	725 ^P^	AFG	Herbacetin di-*O*-Ac-*O*-Hex-*O*-HexA (S3,8) ^L^	+	+	+	+	+
138	11.25 ^iv^	495 ^P^	AFG	Gossypetin 8-*O*-GlcA (=hibifolin) ^S^			+		+
139	11.59 ^iv^	581 ^P^	AFG	Gossypetin *O*-Mal-*O*-HexA (S8) ^L^			+	+	
140	11.98 ^iv^	581 ^P^	AFG	Gossypetin *O*-Mal-*O*-HexA (S8) ^L^	+	+	+		
141	12.26 ^iv^	537 ^P^	AFG	Gossypetin *O*-Ac-*O*-HexA (S8) ^L^	+	+	+	+	
142	12.61 ^iv^	537 ^P^	AFG	Gossypetin *O*-Ac-*O*-HexA (S8) ^L^			+	+	
143	14.01 ^iv^	479 ^P^	AFG	Herbacetin 8-*O*-GlcA (=melocorin) ^S^	+	+	+	+	+
144	14.50 ^iv^	565 ^P^	AFG	Herbacetin *O*-Mal-*O*-HexA (S8) ^L^	+	+	+	+	
145	15.37 ^iv^	521 ^P^	AFG	Herbacetin *O*-Ac-*O*-HexA (S8) ^L^	+	+	+		
146	16.11 ^iv^	521 ^P^	AFG	Herbacetin *O*-Ac-*O*-HexA (S8) ^L^			+		
Total compounds found					90	90	87	100	69
Total known compounds found					21	25	3	5	0
Total compounds found for the first time					69	65	84	95	69

^a^ Chromatographic conditions: ^i^—mode 1; ^ii^—mode 2; ^iii^—mode 3; ^iv^—mode 4. ^b^ Mass spectrometric data: ^N^—deprotonated ion [M − H]^−^, negative ionization; ^P^—protonated ion [M + H]^+^, positive ionization. ^c^ Chemical group of compound: AFG—acidic flavonol glycosides; CT—catechins; HC—hydroxycinnamates; HNG—hydroxynitrile glycosides; NFG—neutral flavonol glycosides; PE—phenylethanoids; PG—phenolic glycosides; PC—procyanidins; TG—terpene glycosides. ^d^ Compound identification was based on comparison of retention time, UV and MS spectral data with reference standard (^S^) or interpretation of UV and MS spectral data and comparison with literature data (^L^). ^e^ In square brackets—reference for known data of compound presence in *R. rosea* organs. Abbreviation used: Ac—acetyl; Ara*f*—arabinofuranose; Ara*p*—arabinopyranose; Glc—glucose; GlcA—glucuronic acid; EGC—epigallocatechin unit; EGCG—epigallocatechin gallate unit; Hex—hexose; HexA—hexuronic acid; Mal—malonyl; Pent—pentose; Rha—rhamnose; Xyl—xylose. Substitution type of flavonol glycoside: S3—3-*O*-substituted; S8—8-*O*-substituted; S37—3,7-di-*O*-substituted; S78—7,8-di-*O*-substituted; S38—3,8-di-*O*-substituted; S378—3,7,8-tri-*O*-substituted.

**Table 6:** Quantitative content of compounds found in *R. rosea* organs, mg/g dry plant weight.

Compound	Roots	Rhizome	Leaves	Flowers	Stems
Total galloyl glucoses:	5.45	3.08	100.63	68.19	10.98
*incl*. mono-galloyl glucoses	0.61	0.30	1.05	0.48	0.60
di-galloyl glucoses	2.29	1.17	10.35	1.56	3.89
tri-galloyl glucoses	0.83	0.42	1.95	0.00	1.04
tetra-galloyl glucoses	0.00	0.00	4.77	7.99	1.42
penta-galloyl glucoses	0.00	0.00	25.60	12.21	0.94
hexa-galloyl glucoses	0.00	0.00	37.79	24.02	2.22
hepta-galloyl glucoses	0.00	0.00	17.45	19.10	0.51
octa-galloyl glucoses	0.00	0.00	0.00	1.31	0.09
Total catechins	10.58	57.40	0.00	0.00	0.12
Total procyanidins:	8.77	34.81	0.00	3.02	0.23
*incl*. dimers	3.50	15.48	0.00	0.19	0.15
trimers	3.47	12.29	0.00	0.00	0.00
tetramers	0.24	1.14	0.00	1.10	0.00
pentamers	1.56	5.90	0.00	1.73	0.08
Hydroquinone glucosides	4.64	8.87	16.62	24.04	2.67
*p*-Hydroxyphenethyl alcohol glucosides	1.97	20.12	0.62	1.87	traces
Hydroxynitrile glucosides	0.97	2.64	1.42	2.66	0.58
Rosiridol glucosides	traces	5.18	0.53	6.45	traces
Cinnamyl alcohols glucosides:	19.89	55.76	0.58	1.26	0.39
*incl*. *p*-hydroxycinnamyl alcohol glucosides	traces	6.30	0.58	1.26	0.39
cinnamyl alcohol glucosides	19.89	47.42	0.00	0.00	0.00
*p*-methoxycinnamyl alcohol glucosides	traces	2.04	0.00	0.00	0.00
Total flavonol glucosides:	1.69	0.50	26.85	55.55	6.30
*incl*. neutral glucoside	0.56	0.08	traces	42.88	1.74
acidic glucoside	1.13	0.42	26.85	12.67	4.56
or *incl*. herbacetin glucosides	0.95	0.33	23.99	33.74	5.51
gossypetin glucosides	0.28	0.11	1.97	20.60	0.72
hibiscetin glucosides	0.00	0.00	0.89	0.92	0.07
kaempferol glucosides	0.46	0.06	traces	0.21	traces
quercetin glucosides	traces	traces	traces	0.08	traces

traces—<0.01 mg/g.

**Table 7:** Percentage reduction of selected marker compounds in *R. rosea* extracts after in vitro treatment by the simulated gastric and intestinal media.

Compounds	Rhizomes Extract		Leaves Extract		Flowers Extract	
	After Gastric Phase	After Intestinal Phase	After Gastric Phase	After Intestinal Phase	After Gastric Phase	After Intestinal Phase
Galloyl glucoses	n.d.	n.d.	65.15	90.20	74.07	94.17
Procyanidins	29.11	94.49	n.d.	n.d.	n.d.	n.d.
Catechins	39.63	96.74	n.d.	n.d.	n.d.	n.d.
Hydroquinone glucosides	7.30	23.71	9.03	28.02	6.03	24.02
*p*-Hydroxyphenethyl alcohol glucosides	3.07	12.52	n.d.	n.d.	n.d.	n.d.
Cinnamyl alcohol glucosides	0.07	1.15	n.d.	n.d.	n.d.	n.d.
Flavonol glucosides	n.d.	n.d.	0.44	0.92	1.39	2.04

n.d.—not detected.

## Supplementary Materials

The following are available online at https://www.mdpi.com/2076-3921/9/6/526/s1.
