# Supplementary material for: LC-MS Profile, Gastrointestinal and Gut Microbiota Stability and Antioxidant Activity of Rhodiola rosea Herb Metabolites: A Comparative Study with Subterranean Organs
Source: Antioxidants (Basel). 2020 Jun 16;9(6):526. doi: 10.3390/antiox9060526 (PMC7346138; doi:10.3390/antiox9060526)
Supplement: Supplementary file 1 [file antioxidants-09-00526-s001.pdf]

antioxidants

Supplementary materials

LC-MS Profile, Gastrointestinal and Gut Microbiota Stability and Antioxidant Activity of *Rhodiola rosea* Herb Metabolites: A Comparative Study with Subterranean Organs

Daniil N. Olennikov [1,*], Nadezhda K. Chirikova [2], Aina G. Vasil'eva[2], and Innokentii A. Fedorov [3]

[1] Laboratory of Medical and Biological Research, Institute of General and Experimental Biology, Siberian Division, Russian Academy of Science, 6 Sakh'yanovoy Street, Ulan-Ude 670047, Russia
[2] Department of Biology, Institute of Natural Sciences, North-Eastern Federal University, 58 Belinsky Street, Yakutsk 677027, Russia; hofnung@mail.ru (N.K.C.); kagamisatou@gmail.com (A.G.V.)
[3] Institute for Biological Problems of Cryolithozone, Siberian Division, Russian Academy of Science, 41 Lenina Street, Yakutsk, 677000, Russia; fedorovia1958@mail.ru
* Correspondence: olennikovdn@mail.ru; Tel.: +7-9021-600-627 (D.N.O.)

Content

Figure S1. High-Performance Liquid Chromatography with Diode Array Detection (HPLC-DAD) chromatograms of C_{18} Sep-Pak ethyl acetate-methanol eluates of *Rhodiola rosea* organ extracts.

Figure S2. HPLC-DAD chromatograms of polyamide eluates I of *R. rosea* organ extracts.

Figure S3. High-Performance Liquid Chromatography with Electrospray Ionization Triple Quadrupole Mass Spectrometric Detection (HPLC-ESI-QQQ-MS) chromatograms of polyamide eluates II of *R. rosea* organ extracts.

Figure S4. HPLC-ESI-QQQ-MS chromatograms of polyamide eluates III of *R. rosea* organ extracts.

Figure S5. HPLC-DAD chromatograms of hydrolysates of total extracts of *R. rosea* organ extracts.

Table S1. Reference standards used for the qualitative and quantitative analysis by HPLC-DAD-ESI-QQQ-MS and HPLC-DAD assays.

Table S2. Regression equations, correlation coefficients, standard deviation, limits of detection, limits of quantification and linear ranges for 44 reference standards used in HPLC-MS quantification.

Table S3. Regression equations, correlation coefficients, standard deviation, limits of detection, limits of quantification and linear ranges for 13 reference standards used in HPLC-DAD quantification.

Table S4. Retention times, UV- and ESI-MS spectral data of compounds **1–146** found in *Rhodiola rosea*.

Table S5. UV-spectral patterns of compounds found in *R. rosea*.

Table S6. Selected spectra of compounds found in *R. rosea*.

Table S7. Known flavonol glycosides of *R. rosea*.

Table S8. Quantitative content of compounds **1–146** in *R. rosea* organs.

Table S9. Content of selected compounds in three *R. rosea* extracts.

Table S10. Content of selected compounds of *R. rosea* rhizome extract (whole probe) before and after *in vitro* treatment by the simulated gastric and intestinal media.

Table S11. Content of selected compounds of *R. rosea* leaves extract (whole probe) before and after *in vitro* treatment by the simulated gastric and intestinal media.

Table S12. Content of selected compounds of *R. rosea* flowers extract (whole probe) before and after *in vitro* treatment by the simulated gastric and intestinal media.

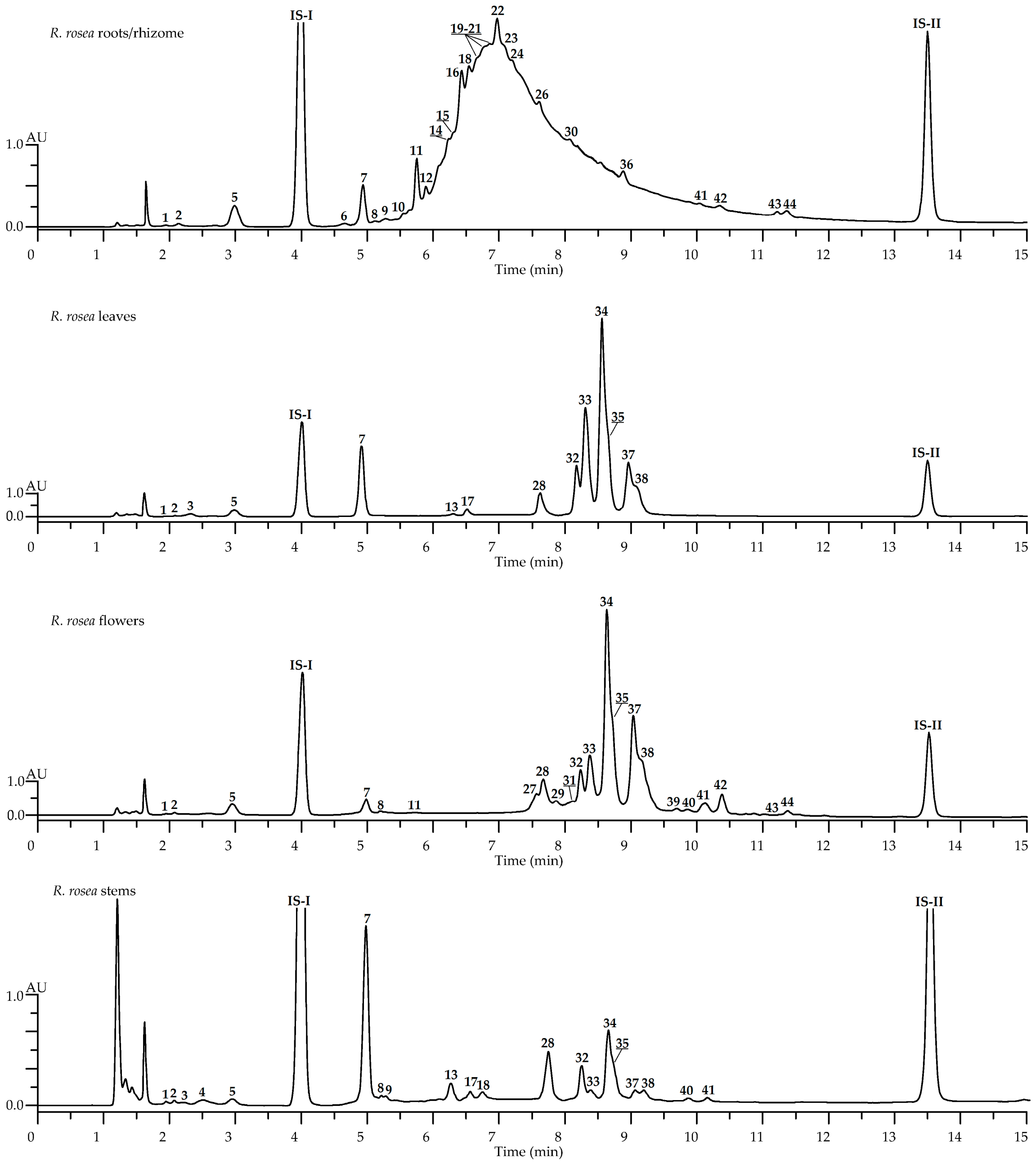

Figure S1. High-Performance Liquid Chromatography with Diode Array Detection (HPLC-DAD) chromatograms (UV detector λ 210 nm) of C_{18} Sep-Pak ethyl acetate-methanol eluates of *Rhodiola rosea* organ extracts. Compounds are numbered as listed in Table 4. IS-I—internal standard I (caffeine, 500 μg/mL); IS-II—internal standard II (benzoic acid, 250 μg/mL).

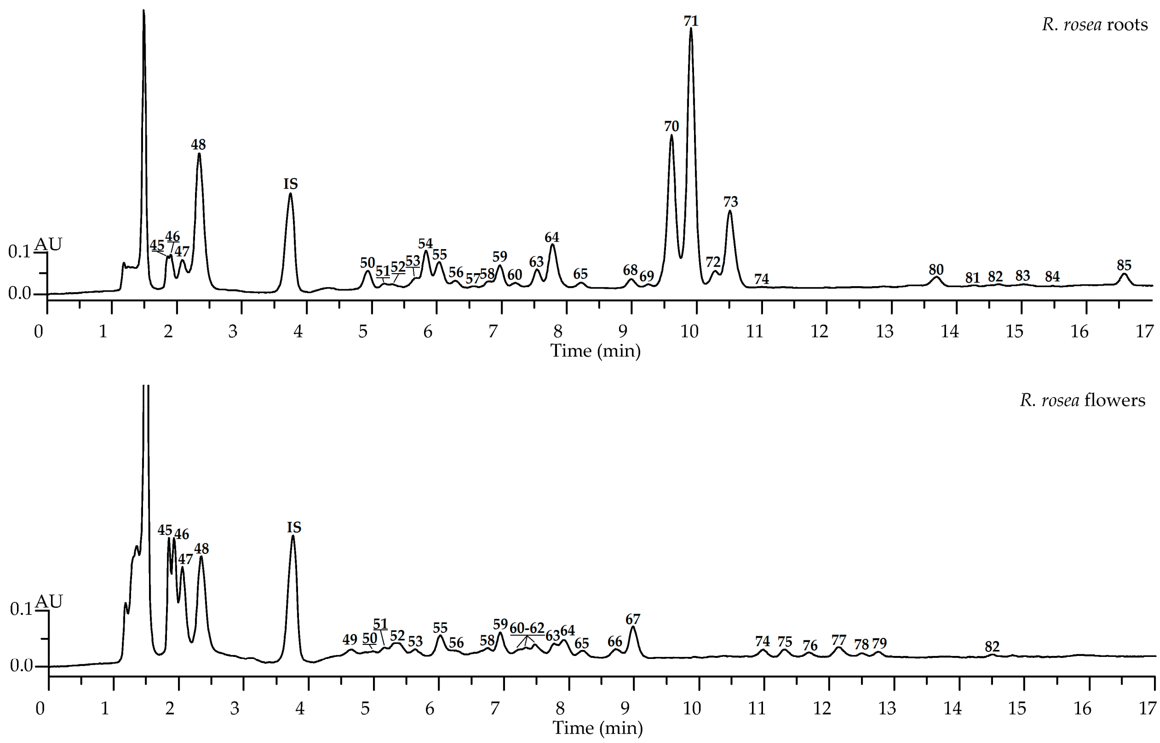

Figure S2. HPLC-DAD chromatograms (UV detector λ 210 nm) of polyamide eluates I of *R. rosea* organ extracts. Compounds are numbered as listed in Table 4. IS—internal standard (picein, 250 μg/mL).

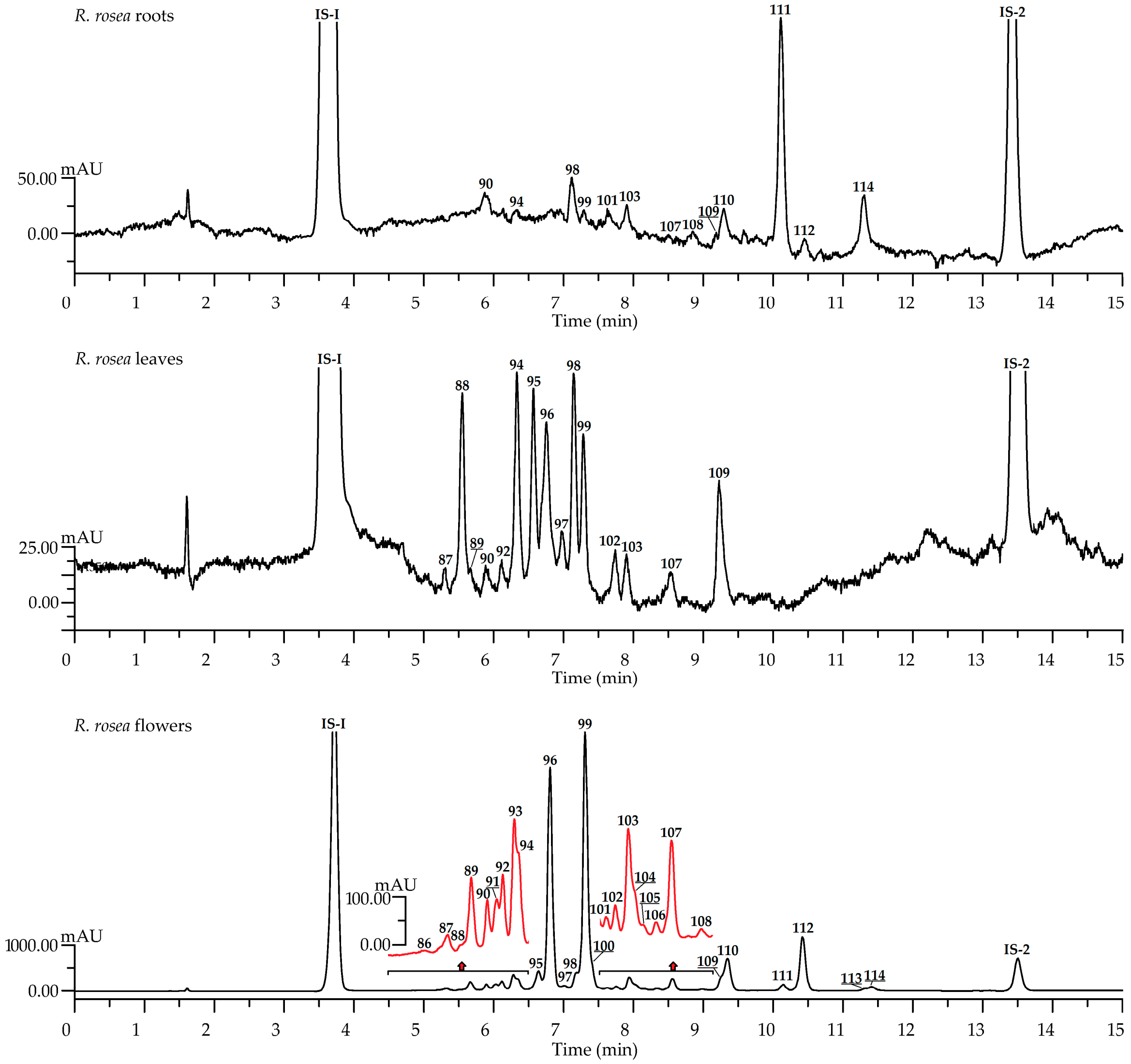

Figure S3. High-Performance Liquid Chromatography with Electrospray Ionization Triple Quadrupole Mass Spectrometric Detection (HPLC-ESI-QQQ-MS) chromatograms (base peak chromatogram or BPC mode, negative ionization) of polyamide eluates II of *R. rosea* organ extracts. Compounds are numbered as listed in Table 4. IS-I—internal standard I (scopoletin-7-*O*-neohesperidoside, 250 µg/mL); IS-II—internal standard II (isorhamnetin, 125 µg/mL). Red inserts—enlarged fragments of chromatogram.

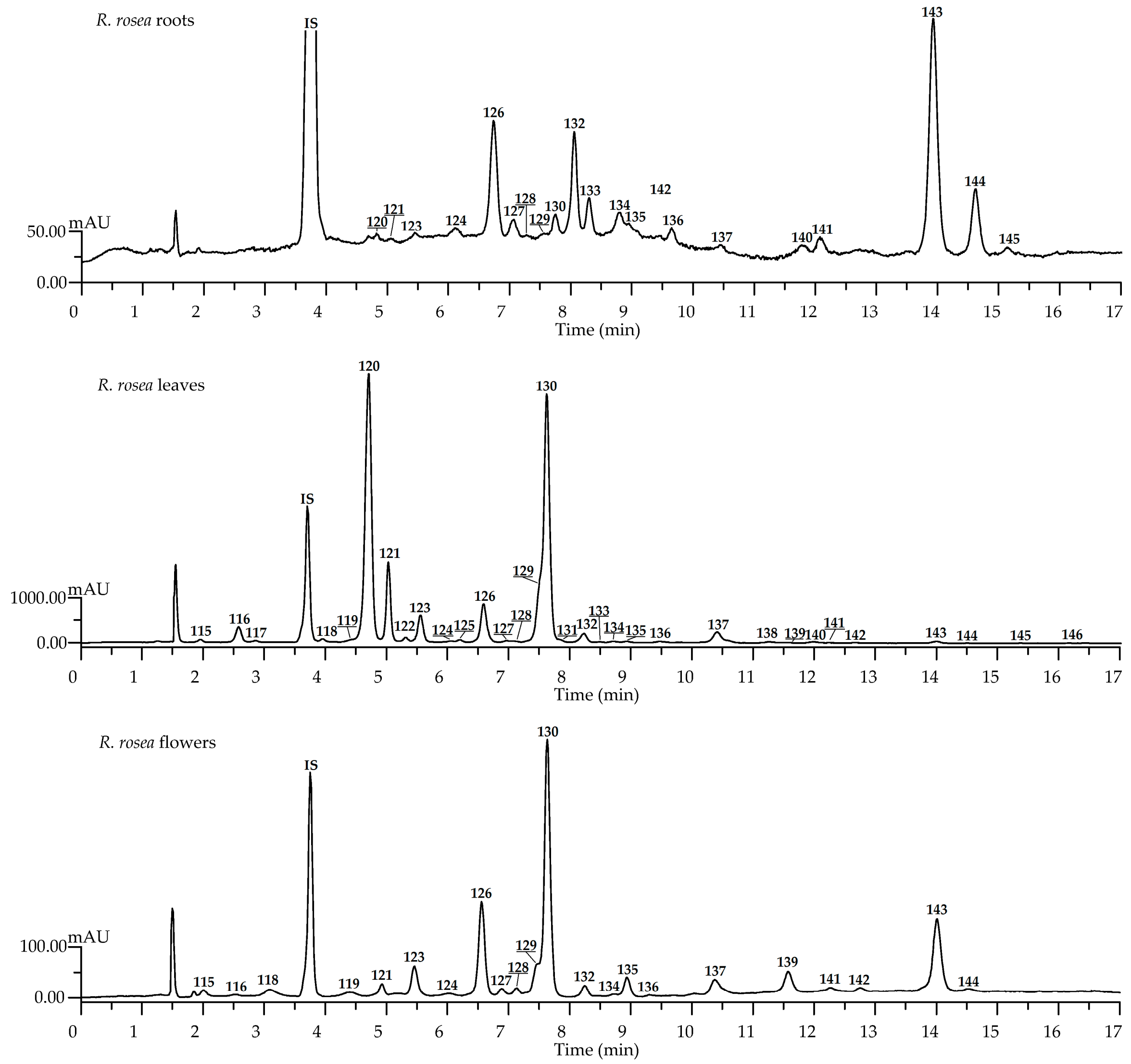

Figure S4. HPLC-ESI-QQQ-MS chromatograms (base peak chromatogram or BPC mode, negative ionization) of polyamide eluates III of *R. rosea* organ extracts. Compounds are numbered as listed in Table 4. IS-I—internal standard (4-*O*-caffeoylquinic acid, 200 µg/mL).

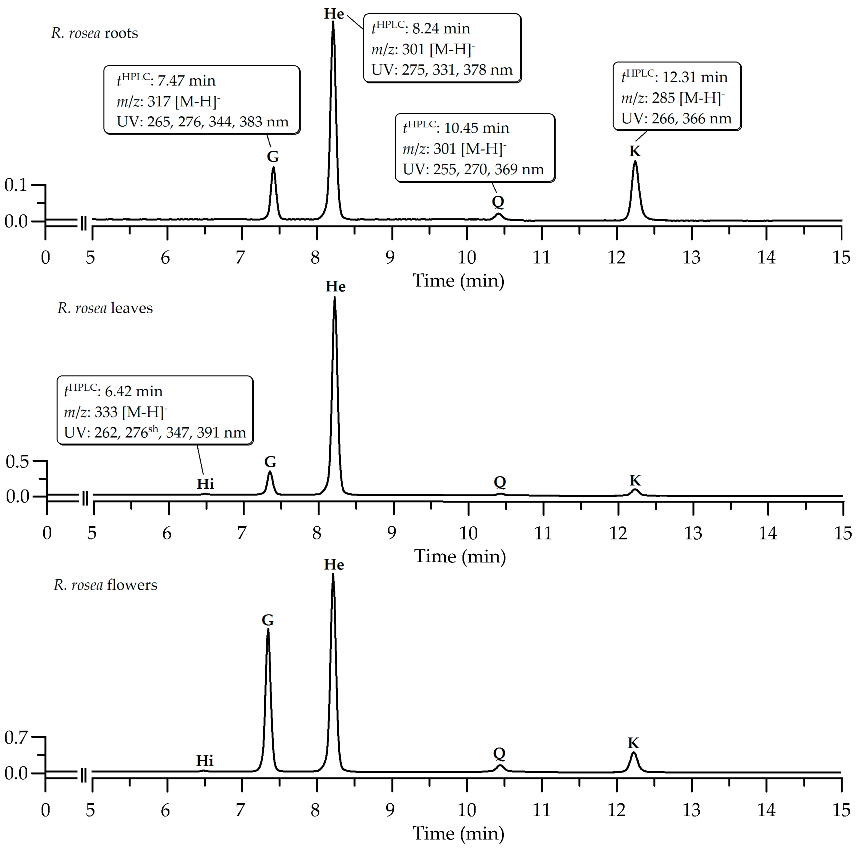

Figure S5. HPLC-DAD chromatograms (detector λ 350 nm) of hydrolysates of total extracts of *R. rosea* organ extracts. G—gossypetin; He—herbacetin; Hi—hibiscetin; K—kaempferol; Q—quercetin. In windows: HPLC retention times (t^{HPLC}), ESI-MS data (*m*/*z*) and UV spectral maxima (UV).

Table S1. Reference standards used for the qualitative and quantitative analysis by HPLC-DAD-ESI-QQQ-MS and HPLC-DAD assays.

No [a]	Compound [b]	Standard	Purity, %	Manufacturer (No) [c]
**1** MS	1-*O*-Galloyl glucose	1-*O*-Galloyl glucose	≥ 90	Sigma (69288)
**2** MS	*O*-Galloyl glucose	1-*O*-Galloyl glucose	≥ 90	Sigma (69288)
**3** MS	*O*-Galloyl glucose	1-*O*-Galloyl glucose	≥ 90	Sigma (69288)
**4** MS	*O*-Galloyl glucose	1-*O*-Galloyl glucose	≥ 90	Sigma (69288)
**5** MS	Gallic acid	Gallic acid	≥ 97	Sigma (G7384)
**6** MS	Di-*O*-galloyl glucose	1,6-Di-*O*-galloyl glucose	≥ 92	Toronto (D293195)
**7** MS,DAD	1,6-Di-*O*-galloyl glucose	1,6-Di-*O*-galloyl glucose	≥ 92	Toronto (D293195)
**8** MS	Procyanidin dimer (EGC-EGC)	Procyanidin B_1	≥ 90	Extrasynthese (0983)
**9** MS	Gallocatechin	(+)Gallocatechin	≥ 98	Extrasynthese (0973S)
**10** MS	Epigallocatechin	(-)Epigallocatechin	≥ 98	Extrasynthese (0979S)
**11** MS,DAD	Procyanidin dimer (EGC-EGCG)	Procyanidin B_1	≥ 90	Extrasynthese (0983)
**12** MS	Catechin	(+)Catechin	≥ 99	Extrasynthese (0976S)
**13** MS	Tri-*O*-galloyl glucose	1,3,6-Tri-*O*-galloyl glucose	≥ 90	Sigma (78864)
**14** MS	Procyanidin dimer (EGCG-EGCG)	Procyanidin B_1	≥ 90	Extrasynthese (0983)
**15** MS	Procyanidin trimer (EGC-EGC-EGC)	Procyanidin B_1	≥ 90	Extrasynthese (0983)
**16** MS,DAD	Procyanidin trimer (EGC-EGC-EGCG)	Procyanidin B_1	≥ 90	Extrasynthese (0983)
**17** MS	1,3,6-Tri-*O*-galloyl glucose	1,3,6-Tri-*O*-galloyl glucose	≥ 90	Sigma (78864)
**18** MS	Tri-*O*-galloyl glucose	1,3,6-Tri-*O*-galloyl glucose	≥ 90	Sigma (78864)
**19** MS	Procyanidin trimer (EGC-EGCG-EGCG)	Procyanidin B_1	≥ 90	Extrasynthese (0983)
**20** MS	Procyanidin trimer (EGCG-EGCG-EGCG)	Procyanidin B_1	≥ 90	Extrasynthese (0983)
**21** MS	Procyanidin tetramer (EGC-EGC-EGC-EGCG)	Procyanidin B_1	≥ 90	Extrasynthese (0983)
**22** MS,DAD	Epicatechin	(-)Epicatechin	≥ 99	Extrasynthese (0977S)
**23** MS,DAD	Epigallocatechin gallate	(-)Epigallocatechin gallate	≥ 98	Extrasynthese (0981S)
**24** MS,DAD	Gallocatechin gallate	(+)Gallocatechin gallate	≥ 98	Extrasynthese (0974S)
**25** MS	Procyanidin tetramer (EGC-EGC-EGCG-EGCG)	Procyanidin B_1	≥ 90	Extrasynthese (0983)
**26** MS	Procyanidin tetramer (EGC-EGCG-EGCG-EGCG)	Procyanidin B_1	≥ 90	Extrasynthese (0983)
**27** MS	Tetra-*O*-galloyl glucose	1,2,3,6-Tetra-*O*-galloyl glucose	≥ 98	BioBioPha (BBPP05354)
**28** MS	1,2,3,6-Tetra-*O*-galloyl glucose	1,2,3,6-Tetra-*O*-galloyl glucose	≥ 98	BioBioPha (BBPP05354)
**29** MS	Tetra-*O*-galloyl glucose	1,2,3,6-Tetra-*O*-galloyl glucose	≥ 98	BioBioPha (BBPP05354)
**30** MS,DAD	Epicatechin gallate	(-)Epicatechin gallate	≥ 97.5	Extrasynthese (0978S)
**31** MS	Tetra-*O*-galloyl glucose	1,2,3,6-Tetra-*O*-galloyl glucose	≥ 98	BioBioPha (BBPP05354)
**32** MS,DAD	Penta-*O*-galloyl glucose	1,2,3,4,6-Penta-*O*-galloyl glucose	≥ 96	Sigma (67548)
**33** MS,DAD	1,2,3,4,6-Penta-*O*-galloyl glucose	1,2,3,4,6-Penta-*O*-galloyl glucose	≥ 96	Sigma (67548)
**34** MS,DAD	Hexa-*O*-galloyl glucose	1,2,3,4,6-Penta-*O*-galloyl glucose	≥ 96	Sigma (67548)
**35** MS,DAD	Hexa-*O*-galloyl glucose	1,2,3,4,6-Penta-*O*-galloyl glucose	≥ 96	Sigma (67548)
**36** MS	Catechin gallate	(+)Catechin gallate	≥ 98	Extrasynthese (0972S)
**37** MS,DAD	Hepta-*O*-galloyl glucose	1,2,3,4,6-Penta-*O*-galloyl glucose	≥ 96	Sigma (67548)
**38** MS,DAD	Hepta-*O*-galloyl glucose	1,2,3,4,6-Penta-*O*-galloyl glucose	≥ 96	Sigma (67548)
**39** MS	Octa-*O*-galloyl glucose	1,2,3,4,6-Penta-*O*-galloyl glucose	≥ 96	Sigma (67548)
**40** MS	Octa-*O*-galloyl glucose	1,2,3,4,6-Penta-*O*-galloyl glucose	≥ 96	Sigma (67548)
**41** MS	Procyanidin tetramer (EGCG-EGCG-EGCG-EGCG)	Procyanidin B_1	≥ 90	Extrasynthese (0983)
**42** MS	Procyanidin pentamer (EGC-EGC-EGC-EGC-EGCG)	Procyanidin B_1	≥ 90	Extrasynthese (0983)
**43** MS	Procyanidin pentamer (EGC-EGC-EGC-EGCG-EGCG)	Procyanidin B_1	≥ 90	Extrasynthese (0983)
**44** MS	Procyanidin pentamer (EGC-EGC-EGCG-EGCG-EGCG)	Procyanidin B_1	≥ 90	Extrasynthese (0983)
**45** MS	Hydroquinone *O*-Hex-*O*-Pent	Arbutin	≥ 98	Sigma (A4256)
**46** MS,DAD	Hydroquinone *O*-Glc (=arbutin)	Arbutin	≥ 98	Sigma (A4256)
**47** MS	Tyrosol *O*-Hex-*O*-Pent	Salidroside	≥ 98	Sigma (43866)
**48** MS,DAD	Tyrosol *O*-Glc (=salidroside)	Salidroside	≥ 98	Sigma (43866)
**49** MS	Tyrosol *O*-dHex	Salidroside	≥ 98	Sigma (43866)
**50** MS	Tyrosol (=*p*-hydroxyphenethyl alcohol)	Tyrosol	≥ 98	Sigma (188255)
**51** MS	Benzyl alcohol *O*-Hex-*O*-Pent	Benzyl alcohol	≥ 99.8	Sigma (305197)
**52** MS	Benzyl alcohol *O*-Hex-*O*-Pent	Benzyl alcohol	≥ 99.8	Sigma (305197)
**53** MS	Benzyl alcohol *O*-Hex	Benzyl alcohol	≥ 99.8	Sigma (305197)
**54** MS	*p*-Hydroxycinnamyl alcohol *O*-Hex-*O*-Pent	Triandrin	≥ 98.5	BioBioPha (BBP05330)
**55** MS	*p*-Hydroxycinnamyl alcohol *O*-Glc (=triandrin)	Triandrin	≥ 98.5	BioBioPha (BBP05330)
**56** MS	Benzyl alcohol	Benzyl alcohol	≥ 99.8	Sigma (305197)
**57** MS	Rhodiocyanoside A/D *O*-Pent	Rhodiocyanoside A	≥ 98	ChemFaces (CFN90973)
**58** MS	Rhodiocyanoside D	Rhodiocyanoside A	≥ 98	ChemFaces (CFN90973)
**59** MS	Rhodiocyanoside A	Rhodiocyanoside A	≥ 98	ChemFaces (CFN90973)
**60** MS	Rosiridol di-*O*-Hex-*O*-Pent	Rosiridin	≥ 98	ChemFaces (CFN99181)
**61** MS	Rosiridol *O*-Hex-di-*O*-Pent	Rosiridin	≥ 98	ChemFaces (CFN99181)
**62** MS	Rosiridol *O*-Hex-di-*O*-Pent	Rosiridin	≥ 98	ChemFaces (CFN99181)
**63** MS	Rosiridol *O*-Hex-*O*-Pent	Rosiridin	≥ 98	ChemFaces (CFN99181)
**64** MS	Rosiridol *O*-Hex-*O*-Pent	Rosiridin	≥ 98	ChemFaces (CFN99181)
**65** MS	Tyrosol *O*-Hex-*O*-Ac	Salidroside	≥ 98	Sigma (43866)
**66** MS	Tyrosol *O*-Hex-*O*-Ac	Salidroside	≥ 98	Sigma (43866)
**67** MS	Tyrosol *O*-Hex-di-*O*-Ac	Salidroside	≥ 98	Sigma (43866)
**68** MS	Cinnamyl alcohol *O*-Hex-di-*O*-Pent	Rosavin	≥ 98	Sigma (SML0336)
**69** MS	Cinnamyl alcohol *O*-Hex-di-*O*-Pent	Rosavin	≥ 98	Sigma (SML0336)
**70** MS,DAD	Cinnamyl alcohol *O*-(6′-*O*-Ara*f*)-Glc (=rosarin)	Rosarin	≥ 98	Sigma (SMB00315)

Table S1. *Cont.*

No	Compound [a]	Standard	Purity, %	Manufacturer (No) [b]
**71** [MS,DAD]	Cinnamyl alcohol *O*-(6′-*O*-Ara*p*)-Glc (=rosavin)	Rosavin	≥ 98	Sigma (SML0336)
**72** [MS]	Cinnamyl alcohol *O*-Hex-*O*-Pent	Rosavin	≥ 98	Sigma (SML0336)
**73** [MS,DAD]	Cinnamyl alcohol *O*-Glc (=rosin)	Rosin	≥ 98	Sigma (PHL89902)
**74** [MS]	Rosiridol 1-*O*-Glc (=rosiridin)	Rosiridin	≥ 98	ChemFaces (CFN99181)
**75** [MS]	*p*-Hydroxycinnamyl alcohol *O*-Hex-*O*-Ac	Rosin	≥ 98	Sigma (PHL89902)
**76** [MS]	*p*-Hydroxycinnamyl alcohol *O*-Hex *O*-Ac	Rosin	≥ 98	Sigma (PHL89902)
**77** [MS]	Rosiridol *O*-Hex-*O*-Ac	Rosiridin	≥ 98	ChemFaces (CFN99181)
**78** [MS]	Rosiridol *O*-Hex-di-*O*-Ac	Rosiridin	≥ 98	ChemFaces (CFN99181)
**79** [MS]	Rosiridol *O*-Hex-di-*O*-Ac	Rosiridin	≥ 98	ChemFaces (CFN99181)
**80** [MS]	*p*-Methoxycinnamyl alcohol *O*-Hex-*O*-Pent	Vimalin	≥ 92	VILAR (0316)
**81** [MS]	*p*-Methoxycinnamyl alcohol *O*-Hex-*O*-Pent	Vimalin	≥ 92	VILAR (0316)
**82** [MS]	*p*-Methoxycinnamyl alcohol *O*-Glc (=vimalin)	Vimalin	≥ 92	VILAR (0316)
**83** [MS]	Cinnamyl alcohol	Cinnamyl alcohol	≥ 98	Sigma (108197)
**84** [MS]	Cinnamic acid	Cinnamic acid	≥ 99	Sigma (C80857)
**85** [MS]	*p*-Methoxycinnamyl alcohol	*p*-Methoxycinnamyl alcohol	≥ 98	ChemFaces (CFN89230)
**86** [MS]	Kaempferol tri-*O*-Hex-*O*-dHex (S37)	Astragalin	≥ 97	Sigma (79851)
**87** [MS]	Herbacetin tri-*O*-Hex-*O*-dHex (S78)	Herbacin	≥ 90	VulcanChem (VC382803)
**88** [MS]	Gossypetin di-*O*-Hex-*O*-dHex (S78)	Hibifolin	≥ 90	Sigma (PHL83580)
**89** [MS]	Quercetin di-*O*-Hex-*O*-dHex (S37)	Isoquercitrin	≥ 98	Sigma (16654)
**90** [MS]	Kaempferol di-*O*-Hex-*O*-dHex (S37)	Kaempferol 7-*O*-Rha	≥ 98	ChemFaces (CFN016224)
**91** [MS]	Gossypetin di-*O*-Hex (S8)	Hibifolin	≥ 90	Sigma (PHL83580)
**92** [MS]	Herbacetin di-*O*-Hex-*O*-dHex (S78)	Herbacin	≥ 90	VulcanChem (VC382803)
**93** [MS]	Gossypetin di-*O*-Hex (S38)	Hibifolin	≥ 90	Sigma (PHL83580)
**94** [MS]	Gossypetin 7-*O*-(3′′-*O*-Glc)-Rha (=rhodioflavonoside)	Herbacin	≥ 90	VulcanChem (VC382803)
**95** [MS]	Herbacetin 7-*O*-(3′′-*O*-Glc)-Rha (=rhodiosin)	Rhodionin	≥ 98	ChemFaces (CFN99758)
**96** [MS,DAD]	Gossypetin 7-*O*-Rha-8-*O*-Glc (=rhodiolgidin)	Herbacin	≥ 90	VulcanChem (VC382803)
**97** [MS]	Kaempferol *O*-Hex-*O*-dHex (S7)	Kaempferol 7-*O*-Rha	≥ 98	ChemFaces (CFN016224)
**98** [MS]	Herbacetin-8-*O*-Xyl (=rhodalin)	Herbacin	≥ 90	VulcanChem (VC382803)
**99** [MS,DAD]	Herbacetin 7-*O*-Rha-8-*O*-Glc (=rhodionidin)	Herbacin	≥ 90	VulcanChem (VC382803)
**100** [MS]	Quercetin 3,7-di-*O*-Glc	Quercetin 3,7-di-*O*-Glc	≥ 92	VILAR (5284-97)
**101** [MS]	Kaempferol 3,7-di-*O*-Glc	Kaempferol 3,7-di-*O*-Glc	≥ 98	ChemFaces (CFN91159)
**102** [MS]	Quercetin 3-*O*-Glc-7-*O*-Rha	Quercetin 3-*O*-Glc-7-*O*-Rha	≥ 98	ChemFaces (CFN90738)
**103** [MS]	Kaempferol 3-*O*-Glc-7-*O*-Rha	Kaempferol 3-*O*-Glc-7-*O*-Rha	≥ 90	VulcanChem (VC383277)
**104** [MS]	Gossypetin 8-*O*-Glc (=gossypin)	Gossypin	≥ 90	Sigma (SML0761)
**105** [MS]	Kaempferol 3,7-di-*O*-Rha (=kaempferitrin)	Kaempferitrin	≥ 98	ChemFaces (CFN98756)
**106** [MS]	Gossypetin 7-*O*-Rha (=rhodiolgin)	Rhodiolgin	≥ 92	VILAR (3710-87)
**107** [MS]	Quercetin 3-*O*-Glc (=isoquercitrin)	Isoquercitrin	≥ 98	Sigma (16654)
**108** [MS]	Quercetin 3-*O*-Rha (=quercitrin)	Quercitrin	≥ 98	Sigma (740580)
**109** [MS]	Herbacetin 8-*O*-Glc (=herbacin)	Herbacin	≥ 90	VulcanChem (VC382803)
**110** [MS]	Herbacetin *O*-dHex (S8)	Herbacin	≥ 90	VulcanChem (VC382803)
**111** [MS]	Kaempferol 3-*O*-Rha (=afzelin)	Afzelin	≥ 98	Sigma (PHL83864)
**112** [MS]	Herbacetin 7-*O*-Rha (=rhodionin)	Rhodionin	≥ 98	ChemFaces (CFN99758)
**113** [MS]	Herbacetin *O*-dHex (S3)	Herbacin	≥ 90	VulcanChem (VC382803)
**114** [MS]	Kaempferol 7-*O*-Rha	Kaempferol 7-*O*-Rha	≥ 98	ChemFaces (CFN016224)
**115** [MS]	Hibiscetin di-*O*-Hex-*O*-HexA (S38)	Hibifolin	≥ 90	Sigma (PHL83580)
**116** [MS]	Gossypetin di-*O*-Hex-*O*-HexA (S378)	Hibifolin	≥ 90	Sigma (PHL83580)
**117** [MS]	Gossypetin *O*-Ac-di-*O*-Hex-*O*-HexA (S378)	Hibifolin	≥ 90	Sigma (PHL83580)
**118** [MS]	Gossypetin *O*-Ac-di-*O*-Hex-*O*-HexA (S378)	Hibifolin	≥ 90	Sigma (PHL83580)
**119** [MS]	Herbacetin di-*O*-Hex-*O*-HexA (S38)	Herbacin	≥ 90	VulcanChem (VC382803)
**120** [MS,DAD]	Herbacetin di-*O*-Hex-*O*-HexA (S378)	Herbacin	≥ 90	VulcanChem (VC382803)
**121** [MS]	Herbacetin *O*-Mal-di-*O*-Hex-*O*-HexA (S378)	Herbacin	≥ 90	VulcanChem (VC382803)
**122** [MS]	Herbacetin *O*-Ac-di-*O*-Hex-*O*-HexA (S378)	Herbacin	≥ 90	VulcanChem (VC382803)
**123** [MS]	Hibiscetin *O*-Hex-*O*-HexA (S38)	Hibifolin	≥ 90	Sigma (PHL83580)
**124** [MS]	Hibiscetin *O*-Mal-*O*-Hex-*O*-HexA (S38)	Hibifolin	≥ 90	Sigma (PHL83580)
**125** [MS]	Hibiscetin *O*-Ac-*O*-Hex-*O*-HexA (S38)	Hibifolin	≥ 90	Sigma (PHL83580)
**126** [MS]	Gossypetin 3-*O*-Glc-8-*O*-GlcA	Hibifolin	≥ 90	Sigma (PHL83580)
**127** [MS]	Gossypetin 3-*O*-(3′′-*O*-Ac)-Glc-8-*O*-GlcA (=rhodiquadrin B)	Hibifolin	≥ 90	Sigma (PHL83580)
**128** [MS]	5-*O*-Feruloylquinic acid	5-*O*-Feruloylquinic acid	≥ 98	ChemFaces (CFN92889)
**129** [MS]	Herbacetin 8-*O*-(2′′-*O*-Glc)-GlcA (=rhodiquadrin C)	Herbacin	≥ 90	VulcanChem (VC382803)
**130** [MS,DAD]	Herbacetin 3-*O*-Glc-8-*O*-GlcA	Herbacin	≥ 90	VulcanChem (VC382803)
**131** [MS]	Herbacetin *O*-Ac-*O*-Hex-*O*-HexA (S3,8)	Herbacin	≥ 90	VulcanChem (VC382803)
**132** [MS]	Herbacetin 3-*O*-(3′′-*O*-Ac)-Glc-8-*O*-GlcA	Herbacin	≥ 90	VulcanChem (VC382803)
**133** [MS]	Gossypetin di-*O*-Ac-*O*-Hex-*O*-HexA (S3,8)	Hibifolin	≥ 90	Sigma (PHL83580)
**134** [MS]	Gossypetin di-*O*-Ac-*O*-Hex-*O*-HexA (S3,8)	Hibifolin	≥ 90	Sigma (PHL83580)
**135** [MS]	Herbacetin *O*-Ac-*O*-Hex-*O*-HexA (S3,8)	Herbacin	≥ 90	VulcanChem (VC382803)
**136** [MS]	Herbacetin *O*-Ac-*O*-Hex-*O*-HexA (S3,8)	Herbacin	≥ 90	VulcanChem (VC382803)
**137** [MS]	Herbacetin di-*O*-Ac-*O*-Hex-*O*-HexA (S3,8)	Herbacin	≥ 90	VulcanChem (VC382803)
**138** [MS]	Gossypetin 8-*O*-GlcA (=hibifolin)	Hibifolin	≥ 90	Sigma (PHL83580)
**139** [MS]	Gossypetin *O*-Mal-*O*-HexA (S8)	Hibifolin	≥ 90	Sigma (PHL83580)
**140** [MS]	Gossypetin *O*-Mal-*O*-HexA (S8)	Hibifolin	≥ 90	Sigma (PHL83580)
**141** [MS]	Gossypetin *O*-Ac-*O*-HexA (S8)	Hibifolin	≥ 90	Sigma (PHL83580)

Table S1. *Cont*.

No	Compound [a]	Standard	Purity, %	Manufacturer (No) [b]
**142** [MS]	Gossypetin *O*-Ac-*O*-HexA (S8)	Hibifolin	≥ 90	Sigma (PHL83580)
**143** [MS]	Herbacetin 8-*O*-GlcA (=melocorin)	Herbacin	≥ 90	VulcanChem (VC382803)
**144** [MS]	Herbacetin *O*-Mal-*O*-HexA (S8)	Herbacin	≥ 90	VulcanChem (VC382803)
**145** [MS]	Herbacetin *O*-Ac-*O*-HexA (S8)	Herbacin	≥ 90	VulcanChem (VC382803)
**146** [MS]	Herbacetin *O*-Ac-*O*-HexA (S8)	Herbacin	≥ 90	VulcanChem (VC382803)

[a] Standards used in chromatographic assays: [MS]—HPLC-DAD-ESI-QQQ-MS (Sections 2.5–2.7); [DAD]—HPLC-DAD (Section 2.9). [b] Abbreviation used: Ac—acetyl; Ara*f*—arabinofuranose; Ara*p*—arabinopyranose; Glc—glucose; GlcA—glucuronic acid; EGC—epigallocatechin unit; EGCG—epigallocatechin gallate unit; Hex—hexose; HexA—hexuronic acid; Mal—malonyl; Pent—pentose; Rha—rhamnose; Xyl—xylose. Substitution type of flavonol glycoside: S3—3-*O*-substituted; S8—8-*O*-substituted; S37—3,7-di-*O*-substituted; S78—7,8-di-*O*-substituted; S38—3,8-di-*O*-substituted; S378—3,7,8-tri-*O*-substituted; S?—unknown type of *O*-substitution. [c] Manufacturers list: BioBioPha—BioBioPha (Kunming, Yunnan, PRC); ChemFaces—ChemFaces (Wuhan, Hubei, PRC); Extrasynthese—Extrasynthese (Lyon, France); Sigma—Sigma-Aldrich (St. Louis, MO, USA); Toronto—Toronto Research Chemicals (North York, ON, Canada); VILAR—Research Institute of Medical and Aromatic Plants (Moscow, Russia); VulcanChem—VulcanChem (Pasadena, CA, USA).

Table S2. Regression equations, correlation coefficients (r^2), standard deviation (S_{YX}), limits of detection (LOD), limits of quantification (LOQ) and linear ranges for 44 reference standards used in HPLC-MS quantification.

Compound	Ionization [a]	CE [b] (eV)	Regression equation [c]		r^2	S_{YX}	LOD/LOQ (μg/mL)	Linear range (μg/mL)
			a	$b \cdot 10^6$				
Gallic acid	N	-20	2.6538	-0.1376	0.9990	$1.17 \cdot 10^{-2}$	0.01/0.04	0.1–100.0
1-*O*-Galloyl glucose	N	-20	1.3586	-0.0663	0.9987	$9.69 \cdot 10^{-2}$	0.24/0.71	0.8–100.0
1,6-Di-*O*-galloyl glucose	N	-20	1.7552	-0.0569	0.9982	$8.89 \cdot 10^{-2}$	0.18/0.51	0.6–100.0
1,3,6-Tri-*O*-galloyl glucose	N	-25	2.1064	-0.0499	0.9985	$8.74 \cdot 10^{-2}$	0.14/0.42	0.5–100.0
1,2,3,6-Tetra-*O*-galloyl glucose	N	-25	2.0168	-0.0629	0.9973	$9.00 \cdot 10^{-2}$	0.15/0.45	0.5–100.0
1,2,3,4,6-Penta-*O*-galloyl glucose	N	-25	2.4561	-0.0171	0.9979	$12.33 \cdot 10^{-2}$	0.17/0.50	0.6–100.0
(+)Catechin	N	-35	0.9562	-0.0521	0.9971	$7.79 \cdot 10^{-2}$	0.27/0.82	0.9–100.0
(-)Epicatechin	N	-35	1.0828	-0.0456	0.9973	$6.85 \cdot 10^{-2}$	0.21/0.63	0.7–100.0
(+)Gallocatechin	N	-35	1.1495	-0.2110	0.9982	$17.02 \cdot 10^{-2}$	0.48/1.48	1.5–100.0
(-)Epigallocatechin	N	-35	1.2824	-0.1440	0.9987	$14.44 \cdot 10^{-2}$	0.37/1.12	1.2–100.0
(+)Catechin gallate	N	-35	1.3387	-0.0284	0.9981	$9.50 \cdot 10^{-2}$	0.23/0.71	0.8–100.0
(-)Epicatechin gallate	N	-35	1.5152	-0.0523	0.9979	$12.67 \cdot 10^{-2}$	0.28/0.84	0.9–100.0
(+)Gallocatechin gallate	N	-35	1.5279	-0.1846	0.9972	$14.95 \cdot 10^{-2}$	0.32/1.98	1.0–100.0
(-)Epigallocatechin gallate	N	-35	1.5824	-0.1078	0.9965	$16.25 \cdot 10^{-2}$	0.34/1.03	1.1–100.0
Procyanidin B_1	N	-25	1.3722	-0.0829	0.9973	$9.93 \cdot 10^{-2}$	0.24/0.72	0.8–100.0
Arbutin	N	-35	0.1756	-0.0144	0.9967	$3.01 \cdot 10^{-2}$	0.56/1.71	2.0–100.0
Salidroside	N	-15	2.0674	-0.1255	0.9972	$9.34 \cdot 10^{-2}$	0.14/0.45	0.5–100.0
Tyrosol	N	-15	2.3277	-0.0933	0.9985	$7.59 \cdot 10^{-2}$	0.11/0.33	0.4–100.0
Benzyl alcohol	N	-18	0.1408	-0.0193	0.9972	$3.91 \cdot 10^{-2}$	0.91/2.78	3.0–100.0
Triandrin	N	-28	0.1916	-0.0217	0.9973	$3.99 \cdot 10^{-2}$	0.68/2.08	2.1–100.0
Rhodiocyanoside A	N	-25	0.6284	-0.0517	0.9975	$5.45 \cdot 10^{-2}$	0.29/0.87	0.9–100.0
Rosiridin	N	-22	0.8036	-0.0062	0.9979	$7.49 \cdot 10^{-2}$	0.31/0.93	1.0–100.0
Rosavin	N	-20	0.8115	-0.1006	0.9980	$2.25 \cdot 10^{-2}$	0.10/0.28	0.3–100.0
Rosarin	N	-20	0.8523	-0.1004	0.9982	$2.08 \cdot 10^{-2}$	0.08/0.24	0.3–100.0
Rosin	N	-20	1.2374	-0.0924	0.9977	$1.88 \cdot 10^{-2}$	0.05/0.15	0.2–100.0
Vimalin	N	-35	1.1242	-0.0315	0.9962	$9.45 \cdot 10^{-2}$	0.28/0.84	0.9–100.0
Cinnamyl alcohol	N	-20	2.7287	-0.0752	0.9994	$7.52 \cdot 10^{-2}$	0.09/0.28	0.3–100.0
Cinnamic acid	N	-20	2.4493	-0.0938	0.9989	$1.85 \cdot 10^{-2}$	0.03/0.08	0.1–100.0
p-Methoxycinnamyl alcohol	N	-35	2.2145	-0.1038	0.9985	$1.48 \cdot 10^{-2}$	0.02/0.07	0.1–100.0
Astragalin	P	+20	2.0859	-0.9171	0.9980	$6.18 \cdot 10^{-2}$	0.03/0.09	0.1–100.0
Afzelin	P	+30	2.2126	-0.5160	0.9987	$8.11 \cdot 10^{-2}$	0.12/0.37	0.4–100.0
Kaempferol 7-*O*-Rha	P	+35	2.1821	-0.5407	0.9982	$4.21 \cdot 10^{-2}$	0.06/0.20	0.2–100.0
Kaempferitrin	P	+35	1.6726	-0.6389	0.9975	$8.29 \cdot 10^{-2}$	0.16/0.50	0.5–100.0
Kaempferol 3-*O*-Glc-7-*O*-Rha	P	+20	1.5787	-0.3641	0.9981	$6.48 \cdot 10^{-2}$	0.14/0.41	0.5–100.0
Kaempferol 3,7-di-*O*-Glc	P	+20	1.4196	-0.4514	0.9989	$5.76 \cdot 10^{-2}$	0.14/0.41	0.5–100.0
Quercitrin	P	+20	1.8267	-0.4160	0.9990	$11.73 \cdot 10^{-2}$	0.21/0.64	0.7–100.0
Isoquercitrin	P	+20	1.6705	-0.4374	0.9988	$12.79 \cdot 10^{-2}$	0.25/0.77	0.8–100.0
Quercetin 3-*O*-Glc-7-*O*-Rha	P	+20	1.4041	-0.3270	0.9992	$14.02 \cdot 10^{-2}$	0.33/1.00	1.0–100.0
Quercetin 3,7-di-*O*-Glc	P	+20	1.3436	-0.4406	0.9981	$17.58 \cdot 10^{-2}$	0.43/1.31	1.4–100.0
Rhodionin	P	+35	1.5742	-0.7787	0.9972	$14.95 \cdot 10^{-2}$	0.31/0.95	1.0–100.0
Herbacin	P	+20	1.4663	-0.6790	0.9975	$15.01 \cdot 10^{-2}$	0.34/1.02	1.1–100.0
Rhodiolgin	P	+30	1.2750	-0.7542	0.9978	$15.82 \cdot 10^{-2}$	0.41/1.24	1.3–100.0
Gossypin	P	+20	1.1880	-0.8182	0.9967	$15.23 \cdot 10^{-2}$	0.43/1.29	1.3–100.0
Hibifolin	P	+40	1.1037	-0.4687	0.9962	$15.78 \cdot 10^{-2}$	0.47/1.43	1.5–100.0
5-*O*-Feruloylquinic acid	N	-20	1.8535	0.0761	0.9989	$4.55 \cdot 10^{-2}$	0.08/0.25	0.3–100.0

[a] Ionization mode : [N]—negative; [P]—positive. [b] CE—collision energy. [c] Regression equation: $y = a \cdot x + b$.

Table S3. Regression equations, correlation coefficients (r^2), standard deviation (S_{YX}), limits of detection (LOD), limits of quantification (LOQ) and linear ranges for 13 reference standards used in HPLC-DAD quantification.

**Compound**	**Regression equation** [a]		r^2	S_{YX}	**LOD/ LOQ (μg/mL)**	**Linear range (μg/mL)**
	a	***b***				
1,6-Di-*O*-galloyl glucose	0.0783	-0.0217	0.9991	$2.40 \cdot 10^{-2}$	1.01/3.06	3.5–100.0
1,2,3,4,6-Penta-*O*-galloyl glucose	0.1064	-0.0121	0.9993	$1.88 \cdot 10^{-2}$	0.58/1.77	2.0–100.0
(-)Epicatechin	0.1138	-0.0255	0.9981	$2.96 \cdot 10^{-2}$	0.86/2.60	3.0–100.0
(-)Epicatechin gallate	0.1465	-0.0336	0.9985	$2.67 \cdot 10^{-2}$	0.60/1.82	2.0–100.0
(+)Gallocatechin gallate	0.1217	-0.0155	0.9987	$3.48 \cdot 10^{-2}$	0.94/2.86	3.0–100.0
(-)Epigallocatechin gallate	0.1740	-0.0177	0.9982	$3.28 \cdot 10^{-2}$	0.62/1.89	2.0–100.0
Procyanidin B_1	0.1716	-0.0109	0.9990	$2.50 \cdot 10^{-2}$	0.48/1.46	1.5–100.0
Arbutin	0.0040	-0.0017	0.9972	$0.41 \cdot 10^{-2}$	2.31/7.00	7.0–100.0
Salidroside	0.0148	-0.0146	0.9974	$0.62 \cdot 10^{-2}$	1.38/4.19	4.5–100.0
Rosavin	0.0483	-0.0105	0.9978	$0.82 \cdot 10^{-2}$	0.56/1.70	2.0–100.0
Rosarin	0.0493	-0.0090	0.9974	$0.75 \cdot 10^{-2}$	0.50/1.52	2.0–100.0
Rosin	0.0642	-0.0080	0.9976	$0.91 \cdot 10^{-2}$	0.47/1.42	2.0–100.0
Herbacin	0.0636	-0.0114	0.9985	$1.21 \cdot 10^{-2}$	0.63/1.90	2.0–100.0

[b] Regression equation: $y = a \cdot x + b$.

Table S4. Retention times (t_R), UV- and ESI-MS spectral data of compounds **1–146** found in *R. rosea*.

No	t_R, min [a]	Compound [b]	UV pattern [c]	CE (eV) [d]	ESI-MS, *m/z*	
					MS [e]	MS/MS (*I*, %) [f]
		**Negative ionization**				
**1**	1.91 [i]	1-*O*-Galloyl glucose [S]	GA	-20	331	[331]: 169 (100), 125 (19)
**2**	2.18 [i]	*O*-Galloyl glucose [L]	GA	-20	331	[331]: 169 (100), 125 (26)
**3**	2.33 [i]	*O*-Galloyl glucose [L]	GA	-20	331	[331]: 169 (100), 125 (9)
**4**	2.57 [i]	*O*-Galloyl glucose [L]	GA	-20	331	[331]: 169 (100), 125 (24)
**5**	2.94 [i]	Gallic acid [S]	GA	-20	169	[169]: 125 (100)
**6**	4.61 [i]	Di-*O*-galloyl glucose [L]	GA	-20	483	[483]: 331 (42), 169 (100), 125 (6)
**7**	4.78 [i]	1,6-Di-*O*-galloyl glucose [S]	GA	-20	483	[483]: 331 (53), 169 (100), 125 (10)
**8**	5.11 [i]	Procyanidin dimer (EGC-EGC) [L]	PC	-20	609	[609]: 305 (100)
**9**	5.27 [i]	Gallocatechin [S]	CT	-35	305	[305]: 168 (37), 125 (100)
**10**	5.52 [i]	Epigallocatechin [S]	CT	-35	305	[305]: 168 (62), 125 (100)
**11**	5.76 [i]	Procyanidin dimer (EGC-EGCG) [L]	PC	-20	761	[761]: 457 (82), 305 (100)
**12**	5.85 [i]	Catechin [S]	CT	-35	289	[289]: 247 (100), 191 (32), 123 (11)
**13**	6.27 [i]	Tri-*O*-galloyl glucose [L]	GA	-25	635	[635]: 483 (63), 331 (100)<br>[483]: 331 (37), 169 (100), 125 (29)
**14**	6.31 [i]	Procyanidin dimer (EGCG-EGCG) [L]	PC	-20	913	[913]: 457 (100)
**15**	6.35 [i]	Procyanidin trimer (EGC-EGC-EGC) [L]	PC	-22	913	[913]: 609 (45), 305 (100)
**16**	6.48 [i]	Procyanidin trimer (EGC-EGC-EGCG) [L]	PC	-22	1065	[1065]: 761 (31), 609 (100), 457 (18)<br>[609]: 457 (32), 305 (100)
**17**	6.54 [i]	1,3,6-Tri-*O*-galloyl glucose [S]	GA	-25	635	[635]: 483 (54), 331 (100)<br>[483]: 331 (42), 169 (100), 125 (11)
**18**	6.78 [i]	Tri-*O*-galloyl glucose [L]	GA	-25	635	[635]: 483 (45), 331 (100)<br>[483]: 331 (31), 169 (100), 125 (16)
**19**	6.82 [i]	Procyanidin trimer (EGC-EGCG-EGCG) [L]	PC	-22	1217	[1217]: 913 (27), 761 (100), 457 (9)<br>[761]: 457 (100), 305 (63)
**20**	6.89 [i]	Procyanidin trimer (EGCG-EGCG-EGCG) [L]	PC	-22	1369	[1369]: 913 (23), 457 (100)
**21**	6.92 [i]	Procyanidin tetramer (EGC-EGC-EGC-EGCG) [L]	PC	-22	1369	[1369]: 1065 (14), 913 (22), 761 (100), 609 (5)<br>[761]: 609 (38), 457 (22), 305 (100)
**22**	7.02 [i]	Epicatechin [S]	CT	-35	289	[289]: 247 (25), 191 (64), 123 (100)
**23**	7.12 [i]	Epigallocatechin gallate [S]	CT	-35	457	[457]: 305 (32), 168 (67), 125 (100)
**24**	7.22 [i]	Gallocatechin gallate [S]	CT	-35	457	[457]: 305 (11), 168 (51), 125 (100)
**25**	7.41 [i]	Procyanidin tetramer (EGC-EGC-EGCG-EGCG) [L]	PC	-22	1521	[1521]: 1217 (2), 913 (11), 761 (100)<br>[761]: 457 (42), 305 (100)
**26**	7.53 [i]	Procyanidin tetramer (EGC-EGCG-EGCG-EGCG) [L]	PC	-22	1673	[1673]: 1369 (4), 1217 (2), 913 (100), 761 (5)<br>[913]: 761 (25), 457 (100), 305 (28)
**27**	7.55 [i]	Tetra-*O*-galloyl glucose [L]	GA	-25	787	[787]: 635 (82), 483 (100), 331 (41)<br>[635]: 483 (67), 331 (100), 169 (59)
**28**	7.63 [i]	1,2,3,6-Tetra-*O*-galloyl glucose [S]	GA	-25	787	[787]: 635 (53), 483 (100), 331 (45)<br>[635]: 483 (54), 331 (100), 169 (39), 125 (8)
**29**	7.83 [i]	Tetra-*O*-galloyl glucose [L]	GA	-25	787	[787]: 635 (71), 483 (100), 331 (36)<br>[635]: 483 (52), 331 (100), 169 (27)
**30**	8.07 [i]	Epicatechin gallate [S]	CT	-35	441	[441]: 289 (12), 125 (43), 109 (100)
**31**	8.14 [i]	Tetra-*O*-galloyl glucose [L]	GA	-25	787	[787]: 635 (62), 483 (100), 331 (42)<br>[635]: 483 (42), 331 (100), 169 (49)
**32**	8.26 [i]	Penta-*O*-galloyl glucose [L]	GA	-25	939	[939]: 787 (52), 635 (100), 483 (19)<br>[635]: 483 (12), 331 (56), 169 (100)
**33**	8.42 [i]	1,2,3,4,6-Penta-*O*-galloyl glucose [S]	GA	-25	939	[939]: 787 (46), 635 (100), 483 (28)<br>[635]: 483 (10), 331 (43), 169 (100)
**34**	8.63 [i]	Hexa-*O*-galloyl glucose [L]	GA	-30	1091	[1091]: 939 (78), 787 (66), 635 (54), 483 (100)<br>[483]: 331 (57), 169 (100)
**35**	8.72 [i]	Hexa-*O*-galloyl glucose [L]	GA	-30	1091	[1091]: 939 (72), 787 (59), 635 (48), 483 (100)<br>[483]: 331 (41), 169 (100)
**36**	8.92 [i]	Catechin gallate [S]	CT	-35	441	[441]: 289 (32), 125 (53), 109 (100)
**37**	8.98 [i]	Hepta-*O*-galloyl glucose [L]	GA	-30	1243	[1243]: 1091 (6), 939 (38), 787 (100), 635 (11)<br>[787]: 635 (10), 483 (41), 331 (100), 169 (25)
**38**	9.14 [i]	Hepta-*O*-galloyl glucose [L]	GA	-30	1243	[1243]: 1091 (9), 939 (45), 787 (100), 635 (18)<br>[787]: 635 (12), 483 (46), 331 (100), 169 (31)
**39**	9.63 [i]	Octa-*O*-galloyl glucose [L]	GA	-30	1395	[1395]: 1243 (4), 1091 (9), 939 (100), 787 (34)<br>[939]: 787 (4), 635 (25), 483 (100), 331 (4)<br>[483]: 331 (25), 169 (100), 125 (11)
**40**	9.87 [i]	Octa-*O*-galloyl glucose [L]	GA	-30	1395	[1395]: 1243 (8), 1091 (9), 939 (100), 787 (21)<br>[939]: 787 (3), 635 (18), 483 (100), 331 (6)<br>[483]: 331 (11), 169 (100), 125 (15)
**41**	10.04 [i]	Procyanidin tetramer (EGCG-EGCG-EGCG-EGCG) [L]	PC	-25	1825	[1825]: 1369 (7), 913 (22), 457 (100)
**42**	10.41 [i]	Procyanidin pentamer (EGC-EGC-EGC-EGC-EGCG) [L]	PC	-25	1673	[1673]: 1369 (2), 1217 (2), 1065 (27), 913 (100), 761 (5); [913]: 761 (18), 609 (100)<br>[609]: 457 (26), 305 (100)

Table S4. *Cont.*

No	t_R, min [a]	Compound [b]	UV pattern [c]	CE (eV) [d]	ESI-MS, *m/z*	
					MS [e]	MS[(n)] (*I*, %) [f]
**43**	11.18 [i]	Procyanidin pentamer (EGC-EGC-EGC-EGCG-EGCG) [L]	PC	-25	1825	[1825]: 1521 (1), 1369 (3), 1217 (26), 1065 (100), 913 (11)<br>[1065]: 913 (36), 761 (100), 457 (30), 305 (14)
**44**	11.43 [i]	Procyanidin pentamer (EGC-EGC-EGCG-EGCG-EGCG) [L]	PC	-25	1977	[1977]: 1673 (1), 1521 (1), 1369 (10), 1065 (15), 913 (100)<br>[913]: 609 (37), 457 (100), 305 (26)
**45**	1.90 [ii]	Hydroquinone *O*-Hex-*O*-Pent [L]	HQ	-35	403	[403]: 271 (33), 109 (100)
**46**	1.98 [ii]	Hydroquinone *O*-Glc (=arbutin) [S]	HQ	-35	271	[271]: 109 (100)
**47**	2.09 [ii]	Tyrosol *O*-Hex-*O*-Pent [L]	TY	-17	431	[431]: 299 (27), 137 (100), 119 (9)
**48**	2.37 [ii]	Tyrosol *O*-Glc (=salidroside) [S]	TY	-15	299	[299]: 137 (100), 119 (31), 89 (34)
**49**	4.59 [ii]	Tyrosol *O*-dHex [L]	TY	-15	283	[283]: 137 (100), 119 (27), 89 (21)
**50**	4.95 [ii]	Tyrosol (=*p*-hydroxyphenethyl alcohol) [S]	TY	-15	137	[137]: 119 (82), 89 (100)
**51**	5.11 [ii]	Benzyl alcohol *O*-Hex-*O*-Pent [L]	BZ	-18	401	[401]: 269 (25), 107 (100), 77 (38)
**52**	5.23 [ii]	Benzyl alcohol *O*-Hex-*O*-Pent [L]	BZ	-18	401	[401]: 269 (34), 107 (100), 77 (21)
**53**	5.56 [ii]	Benzyl alcohol *O*-Hex [L]	BZ	-18	269	[269]: 107 (100), 77 (52)
**54**	5.82 [ii]	*p*-Hydroxycinnamyl alcohol *O*-Hex-*O*-Pent [L]	pHCA	-28	443	[443]: 311 (14), 149 (100), 131 (9)
**55**	6.02 [ii]	*p*-Hydroxycinnamyl alcohol *O*-Glc (=triandrin) [S]	pHCA	-28	311	[311]: 149 (100), 131 (54)
**56**	6.27 [ii]	Benzyl alcohol [S]	BZ	-18	107	[107]: 77 (100)
**57**	6.55 [ii]	Rhodiocyanoside A/D *O*-Pent [L]	HNG	-25	390	[390]: 258 (41), 96 (100)
**58**	6.76 [ii]	Rhodiocyanoside D [L]	HNG	-25	258	[258]: 96 (100)
**59**	6.97 [ii]	Rhodiocyanoside A [S]	HNG	-25	258	[258]: 96 (100)
**60**	7.18 [ii]	Rosiridol di-*O*-Hex-*O*-Pent [L]	NA	-22	625	[625]: 493 (11), 331 (22), 169 (100)
**61**	7.27 [ii]	Rosiridol *O*-Hex-di-*O*-Pent [L]	NA	-22	595	[595]: 463 (9), 331 (15), 169 (100)
**62**	7.47 [ii]	Rosiridol *O*-Hex-di-*O*-Pent [L]	NA	-22	595	[595]: 463 (11), 331 (18), 169 (100)
**63**	7.52 [ii]	Rosiridol *O*-Hex-*O*-Pent [L]	NA	-22	463	[463]: 331 (21), 169 (100)
**64**	7.81 [ii]	Rosiridol *O*-Hex-*O*-Pent [L]	NA	-22	463	[463]: 331 (24), 169 (100)
**65**	8.18 [ii]	Tyrosol *O*-Hex-*O*-Ac [L]	TY	-20	341	[341]: 299 (8), 137 (100), 119 (24)
**66**	8.72 [ii]	Tyrosol *O*-Hex-*O*-Ac [L]	TY	-20	341	[341]: 299 (5), 137 (100), 119 (11)
**67**	8.98 [ii]	Tyrosol *O*-Hex-di-*O*-Ac [L]	TY	-20	383	[383]: 299 (34), 137 (100), 119 (5)
**68**	9.02 [ii]	Cinnamyl alcohol *O*-Hex-di-*O*-Pent [L]	CIN	-20	559	[559]: 427 (8), 295 (14), 133 (100)
**69**	9.26 [ii]	Cinnamyl alcohol *O*-Hex-di-*O*-Pent [L]	CIN	-20	559	[559]: 427 (11), 295 (27), 133 (100)
**70**	9.57 [ii]	Cinnamyl alcohol *O*-(6′-*O*-Ara*f*)-Glc (=rosarin) [S]	CIN	-20	427	[427]: 295 (18), 133 (100)
**71**	9.95 [ii]	Cinnamyl alcohol *O*-(6′-*O*-Ara*p*)-Glc (=rosavin) [S]	CIN	-20	427	[427]: 295 (37), 133 (100)
**72**	10.26 [ii]	Cinnamyl alcohol *O*-Hex-*O*-Pent [L]	CIN	-20	427	[427]: 295 (22), 133 (100)
**73**	10.51 [ii]	Cinnamyl alcohol *O*-Glc (=rosin) [S]	CIN	-20	295	[295]: 133 (100)
**74**	10.98 [ii]	Rosiridol 1-*O*-Glc (=rosiridin) [S]	NA	-22	331	[331]: 169 (100)
**75**	11.27 [ii]	*p*-Hydroxycinnamyl alcohol *O*-Hex-*O*-Ac [L]	pHCA	-30	353	[353]: 311 (32), 149 (100), 131 (5)
**76**	11.67 [ii]	*p*-Hydroxycinnamyl alcohol *O*-Hex *O*-Ac [L]	pHCA	-30	353	[353]: 311 (28), 149 (100), 131 (7)
**77**	12.14 [ii]	Rosiridol *O*-Hex-*O*-Ac [L]	NA	-25	373	[373]: 331 (7), 169 (100)
**78**	12.48 [ii]	Rosiridol *O*-Hex-di-*O*-Ac [L]	NA	-25	415	[415]: 331 (26), 169 (100)
**79**	12.81 [ii]	Rosiridol *O*-Hex-di-*O*-Ac [L]	NA	-25	415	[415]: 331 (34), 169 (100)
**80**	13.65 [ii]	*p*-Methoxycinnamyl alcohol *O*-Hex-*O*-Pent [L]	pMCA	-35	457	[457]: 325 (21), 163 (100), 149 (9)
**81**	13.94 [ii]	*p*-Methoxycinnamyl alcohol *O*-Hex-*O*-Pent [L]	pMCA	-35	457	[457]: 325 (27), 163 (100), 149 (6)
**82**	14.57 [ii]	*p*-Methoxycinnamyl alcohol *O*-Glc (=vimalin) [S]	pMCA	-35	325	[325]: 163 (100), 149 (10)
**83**	15.06 [ii]	Cinnamyl alcohol [S]	CIN	-20	133	
**84**	15.47 [ii]	Cinnamic acid [S]	CAc	-20	147	
**85**	16.58 [ii]	*p*-Methoxycinnamyl alcohol [S]	pMCA	-35	163	[163]: 149 (100)
**128**	7.27 [iv]	3-*O*-Feruloylquinic acid	FQA	-20	367	[367]: 193 (100), 173 (5), 149 (2)
		**Positive ionization**				
**86**	5.02 [iii]	Kaempferol tri-*O*-Hex-*O*-dHex [L]	K37	+20	919	[919]: 757 (23), 595 (100), 433 (31)<br>[595]: 433 (100), 287 (21)
**87**	5.41 [iii]	Herbacetin tri-*O*-Hex-*O*-dHex [L]	H78	+20	935	[935]: 773 (11), 611 (100), 449 (27)<br>[611]: 449 (100), 303 (27)
**88**	5.56 [iii]	Gossypetin di-*O*-Hex-*O*-dHex [L]	G78	+20	789	[789]: 627 (32), 465 (100), 319 (17)
**89**	5.63 [iii]	Quercetin di-*O*-Hex-*O*-dHex [L]	Q37	+20	773	[773]: 611 (15), 449 (100), 303 (12)
**90**	5.97 [iii]	Kaempferol di-*O*-Hex-*O*-dHex [L]	K37	+20	757	[757]: 595 (18), 433 (100), 287 (28)
**91**	6.18 [iii]	Gossypetin di-*O*-Hex [L]	G8	+20	643	[643]: 481 (15), 319 (100)
**92**	6.21 [iii]	Herbacetin di-*O*-Hex-*O*-dHex [L]	H78	+20	773	[773]: 611 (38), 449 (100), 303 (8)
**93**	6.32 [iii]	Gossypetin di-*O*-Hex [L]	G38	+20	643	[643]: 481 (18), 319 (100)
**94**	6.43 [iii]	Gossypetin 7-*O*-(3′′-*O*-Glc)-Rha (=rhodioflavonoside) [S]	G7	+20	627	[627]: 465 (100), 319 (15)
**95**	6.63 [iii]	Herbacetin 7-*O*-(3′′-*O*-Glc)-Rha (=rhodiosin) [S]	H7	+20	611	[611]: 449 (100), 303 (21)
**96**	6.82 [iii]	Gossypetin 7-*O*-Rha-8-*O*-Glc (=rhodiolgidin) [S]	G78	+20	627	[627]: 465 (100), 319 (52)
**97**	7.02 [iii]	Kaempferol *O*-Hex-*O*-dHex [L]	K7	+20	595	[595]: 433 (100), 287 (31)
**98**	7.21 [iii]	Herbacetin-8-*O*-Xyl (=rhodalin) [S]	H8	+20	435	[435]: 303 (100)
**99**	7.35 [iii]	Herbacetin 7-*O*-Rha-8-*O*-Glc (=rhodionidin) [S]	H78	+20	611	[611]: 449 (100), 303 (41)
**100**	7.43 [iii]	Quercetin 3,7-di-*O*-Glc [S]	Q37	+20	627	[627]: 465 (57), 303 (100)
**101**	7.54 [iii]	Kaempferol 3,7-di-*O*-Glc [S]	K37	+20	611	[611]: 449 (38), 287 (100)

Table S4. *Cont.*

No	t_R, min [a]	Compound [b]	UV pattern [c]	CE (eV) [d]	ESI-MS, *m*/*z*	
					MS [e]	MS(n) (*I*, %) [f]
**102**	7.75 [iii]	Quercetin 3-*O*-Glc-7-*O*-Rha [S]	Q37	+20	611	[611]: 449 (100), 303 (31)
**103**	7.97 [iii]	Kaempferol 3-*O*-Glc-7-*O*-Rha [S]	K37	+20	595	[595]: 433 (100), 287 (22)
**104**	8.01 [iii]	Gossypetin 8-*O*-Glc (=gossypin) [S]	G8	+20	481	[481]: 319 (100)
**105**	8.11 [iii]	Kaempferol 3,7-di-*O*-Rha (=kaempferitrin) [S]	K37	+35	579	[579]: 433 (100), 287 (15)
**106**	8.41 [iii]	Gossypetin 7-*O*-Rha (=rhodiolgin) [S]	G7	+30	465	[465]: 319 (100)
**107**	8.57 [iii]	Quercetin 3-*O*-Glc (=isoquercitrin) [S]	Q3	+20	465	[465]: 303 (100)
**108**	8.98 [iii]	Quercetin 3-*O*-Rha (=quercitrin) [S]	Q3	+20	449	[449]: 303 (100)
**109**	9.22 [iii]	Herbacetin 8-*O*-Glc (=alginin) [S]	H8	+20	465	[465]: 303 (100)
**110**	9.43 [iii]	Herbacetin *O*-dHex [L]	H8	+20	449	[449]: 303 (100)
**111**	10.12 [iii]	Kaempferol 3-*O*-Rha (=afzelin) [S]	K3	+30	433	[433]: 287 (100)
**112**	10.46 [iii]	Herbacetin 7-*O*-Rha (=rhodionin) [S]	H7	+35	449	[449]: 303 (100)
**113**	11.27 [iii]	Herbacetin *O*-dHex [L]	H3	+20	449	[449]: 303 (100)
**114**	11.45 [iii]	Kaempferol 7-*O*-Rha	K7	+35	433	[433]: 287 (100)
**115**	1.97 [iv]	Hibiscetin di-*O*-Hex-*O*-HexA [L]	Hi38	+35	835	[835]: 673 (18), 511 (100), 335 (22)
**116**	2.58 [iv]	Gossypetin di-*O*-Hex-*O*-HexA [L]	G378	+35	819	[819]: 657 (90), 495 (100), 319 (29)
**117**	2.82 [iv]	Gossypetin *O*-Ac-di-*O*-Hex-*O*-HexA [L]	G378	+35	861	[861]: 819 (5), 657 (29), 495 (100), 319 (16)
**118**	3.15 [iv]	Gossypetin *O*-Ac-di-*O*-Hex-*O*-HexA [L]	G378	+35	861	[861]: 819 (3), 657 (22), 495 (100), 319 (27)
**119**	4.42 [iv]	Herbacetin di-*O*-Hex-*O*-HexA [L]	H38	+35	803	[803]: 641 (74), 479 (100), 303 (14)
**120**	4.63 [iv]	Herbacetin di-*O*-Hex-*O*-HexA [L]	H378	+35	803	[803]: 641 (84), 479 (100), 303 (10)
**121**	5.02 [iv]	Herbacetin *O*-Mal-di-*O*-Hex-*O*-HexA [L]	H378	+35	889	[889]: 803 (25), 641 (37), 479 (100), 303 (29)
**122**	5.27 [iv]	Herbacetin *O*-Ac-di-*O*-Hex-*O*-HexA [L]	H378	+35	845	[845]: 803 (27), 641 (31), 479 (100), 303 (21)
**123**	5.52 [iv]	Hibiscetin *O*-Hex-*O*-HexA [L]	Hi38	+35	673	[673]: 511 (100), 335 (18)
**124**	6.03 [iv]	Hibiscetin *O*-Mal-*O*-Hex-*O*-HexA [L]	Hi38	+35	759	[759]: 673 (21), 511 (100), 335 (18)
**125**	6.18 [iv]	Hibiscetin *O*-Ac-*O*-Hex-*O*-HexA [L]	Hi38	+35	715	[715]: 673 (25), 511 (100), 335 (15)
**126**	6.53 [iv]	Gossypetin 3-*O*-Glc-8-*O*-GlcA [S]	G38	+35	657	[657]: 495 (100), 319 (22)
**127**	6.97 [iv]	Gossypetin 3-*O*-(3′′-*O*-Ac)-Glc-8-*O*-GlcA (=rhodiquadrin B) [S]	G38	+35	699	[699]: 523 (100), 319 (46)
**129**	7.51 [iv]	Herbacetin 8-*O*-(2′′-*O*-Glc)-GlcA (=rhodiquadrin C) [S]	H8	+35	641	[641]: 479 (100), 303 (43)
**130**	7.62 [iv]	Herbacetin 3-*O*-Glc-8-*O*-GlcA [S]	H38	+35	641	[641]: 479 (100), 303 (8)
**131**	7.90 [iv]	Herbacetin *O*-Ac-*O*-Hex-*O*-HexA [L]	H38	+35	683	[683]: 507 (100), 303 (35)
**132**	8.23 [iv]	Herbacetin 3-*O*-(3′′-*O*-Ac)-Glc-8-*O*-GlcA [S]	H38	+35	683	[683]: 507 (100), 303 (32)
**133**	8.48 [iv]	Gossypetin di-*O*-Ac-*O*-Hex-*O*-HexA [L]	G38	+35	741	[741]: 565 (100), 319 (31)
**134**	8.73 [iv]	Gossypetin di-*O*-Ac-*O*-Hex-*O*-HexA [L]	G38	+35	741	[741]: 565 (100), 319 (28)
**135**	8.98 [iv]	Herbacetin *O*-Ac-*O*-Hex-*O*-HexA [L]	H38	+35	683	[683]: 507 (100), 303 (25)
**136**	9.49 [iv]	Herbacetin *O*-Ac-*O*-Hex-*O*-HexA [L]	H38	+35	683	[683]: 507 (100), 303 (29)
**137**	10.47 [iv]	Herbacetin di-*O*-Ac-*O*-Hex-*O*-HexA [L]	H38	+35	725	[725]: 549 (100), 303 (22)
**138**	11.25 [iv]	Gossypetin 8-*O*-GlcA(=hibifolin) [S]	G8	+40	495	[495]: 319 (100)
**139**	11.59 [iv]	Gossypetin *O*-Mal-*O*-HexA [L]	G8	+35	581	[581]: 495 (100), 319 (72)
**140**	11.98 [iv]	Gossypetin *O*-Mal-*O*-HexA [L]	G8	+35	581	[581]: 495 (100), 319 (65)
**141**	12.26 [iv]	Gossypetin *O*-Ac-*O*-HexA [L]	G8	+35	537	[537]: 495 (100), 319 (62)
**142**	12.61 [iv]	Gossypetin *O*-Ac-*O*-HexA [L]	G8	+35	537	[537]: 495 (100), 319 (67)
**143**	14.01 [iv]	Herbacetin 8-*O*-GlcA (=melocorin) [S]	H8	+40	479	[479]: 303 (100)
**144**	14.50 [iv]	Herbacetin *O*-Mal-*O*-HexA [L]	H8	+35	565	[565]: 479 (100), 303 (82)
**145**	15.37 [iv]	Herbacetin *O*-Ac-*O*-HexA [L]	H8	+35	521	[521]: 479 (100), 303 (73)
**146**	16.11 [iv]	Herbacetin *O*-Ac-*O*-HexA [L]	H8	+35	521	[521]: 479 (100), 303 (70)

[a] Chromatographic conditions: [i]—mode 1; [ii]—mode 2; [iii]—mode 3; [iv]—mode 4. [b] Compound identification was based on comparison of retention time, UV and MS spectral data with reference standard ([S]) or interpretation of UV and MS spectral data and comparison with literature data ([L]). [c] UV-patterns as listed in Table S5. [d] CE—collision energy. [e] Mass spectrometric data: deprotonated ion $[M–H]^−$, negative ionization / protonated ion $[M+H]^+$, positive ionization. [f] Signal intensity (percentage). Abbreviation used: Ac—acetyl; Ara*f*—arabinofuranose; Ara*p*—arabinopyranose; Glc—glucose; GlcA—glucuronic acid; Hex—hexose; HexA—hexuronic acid; Mal—malonyl; Pent—pentose; Rha—rhamnose; Xyl—xylose.

Table S5. UV-spectral patterns of compounds found in *R. rosea*.

**Name of UV-pattern**	**Group of compound**	**λ_{max}, nm**
BZ	Benzyl alcohol derivatives	205 (±2)
CAc	Cinnamic acid	272
CIN	Cinnamyl alcohol derivatives	250 (±2)
CT	Catechins	275 (±1)
FQA	Feruloylquinic acid	324
G7	Gossypetin 7-*O*-glucosides	260 (±3), 276 sh (±3), 345 (±4), 385 (±5)
G8	Gossypetin 8-*O*-glucosides	256 (±3), 270 sh (±3), 305 (±3), 377 (±5)
G38	Gossypetin 3,8-di-*O*-glucosides	258 (±3), 270 (±2), 355 (±5)
G78	Gossypetin 7,8-di-*O*-glucosides	260 sh (±3), 276 (±3), 304 sh (±4), 340 (±5), 375 sh (±5)
G378	Gossypetin 3,7,8-tri-*O*-glucosides	260 (±3), 270 sh (±3), 365 (±6)
GA	Galloyl glucoses	277 (±2), 300 sh (±4)
H3	Herbacetin 3-*O*-glucosides	282 (±3), 302 (±5), 330 (±5), 375 (±7)
H7	Herbacetin 7-*O*-glucosides	276 (±3), 330 (±5), 385 (±5)
H8	Herbacetin 8-*O*-glucosides	255 sh (±3), 272 (±3), 326 (±4), 374 (±5)
H37	Herbacetin 3,7-di-*O*-glucosides	275 (±3), 306 (±3), 330 (±3), 370 sh (±4)
H38	Herbacetin 3,8-di-*O*-glucosides	272 (±3), 330 sh (±2), 360 (±5)
H78	Herbacetin 7,8-di-*O*-glucosides	276 (±2), 306 (±3), 332 (±3), 370 sh (±6)
H378	Herbacetin 3,7,8-tri-*O*-glucosides	270 (±3), 326 sh (±3), 356 (±4)
Hi38	Hibiscetin 3,8-di-*O*-glucosides	255 sh (±4), 270 (±4), 300 (±2), 365 (±5)
HNG	Hydroxynitrile glucosides	207 (±1)
HQ	Hydroquinone derivatives	282 (±1)
K3	Kaempferol 3-*O*-glucosides	265 (±3), 290 (±2), 320 (±3), 345 (±6)
K7	Kaempferol 7-*O*-glucosides	252 (±3), 265 (±3), 322 (±3), 360 (±5)
K37	Kaempferol 3,7-di-*O*-glucosides	245 sh (±3), 265 (±2), 315 sh (±3), 350 (±6)
NA	No absorption > 200 nm	195–200
PC	Procyanidins	270 (±2)
pHCA	*p*-Hydroxycinnamyl alcohol derivatives	280 (±2)
pMCA	p-Methoxycinnamyl alcohol derivatives	306 (±2)
Q3	Quercetin 3-*O*-glucosides	256 (±3), 267 (±2), 292 (±3), 362 (±4)
Q37	Quercetin 3,7-di-*O*-glucosides	255 (±3), 267 (±3), 355 (±5)
TY	Tyrosol derivatives	225 (±2), 275 (±2)

sh—shoulder.

Table S6. Selected spectra of compounds found in *R. rosea*.

No	Compound	MS/MS spectra
**34**	Hexa-*O*-galloyl glucose<br>(galloyl *O*-glycoside)<br>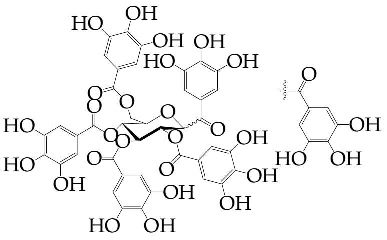<br>(tentative)	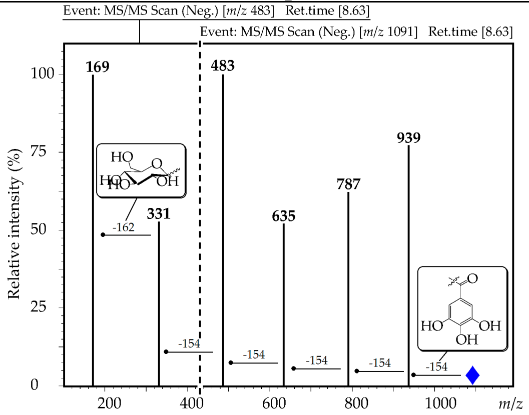
**42**	Procyanidin pentamer<br>(EGC-EGC-EGC-EGC-EGCG)<br>(procyanidin)<br>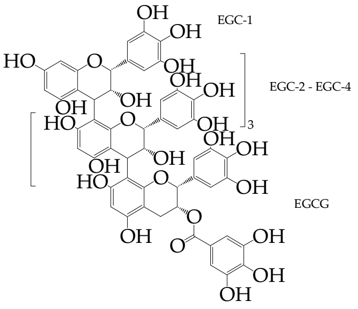<br>(tentative)	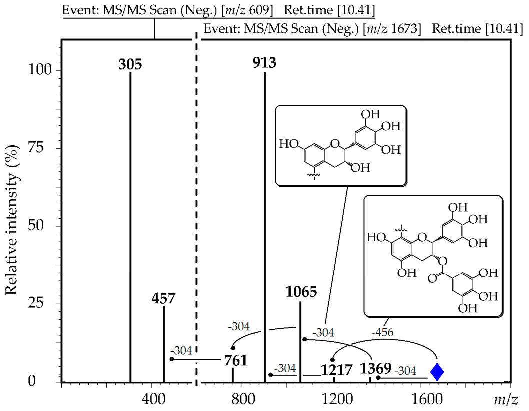
**45**	Hydroquinone *O*-hexoside-*O*-pentoside<br>(hydroquinone derivative)<br>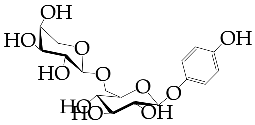<br>(tentative)	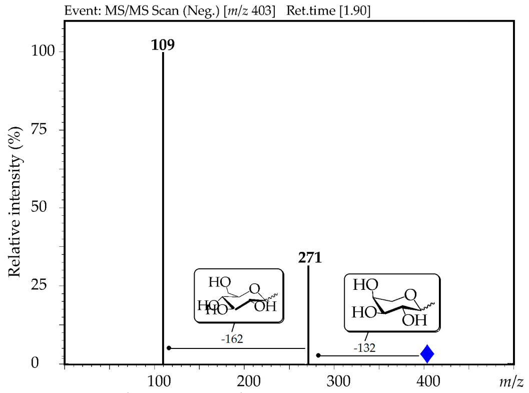
**47**	Tyrosol *O*-hexoside-*O*-pentoside<br>(tyrosol derivative)<br>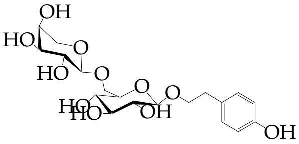<br>(tentative)	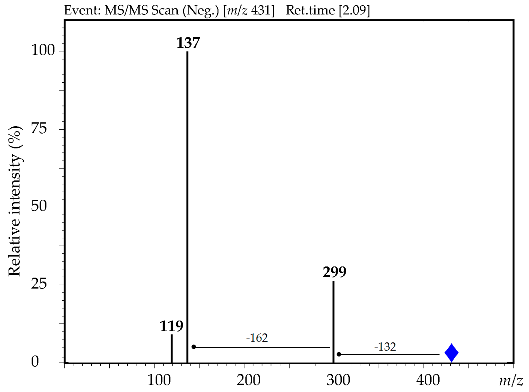

Table S6. *Cont.*

No	Compound	MS/MS spectra
**52**	Benzyl alcohol *O*-hexoside-*O*-pentoside (benzyl alcohol derivative) 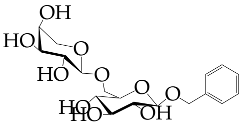 (tentative)	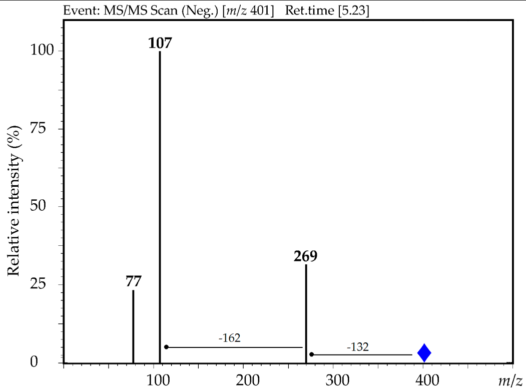
**62**	Rosiridol *O*-hexoside-di-*O*-pentoside (rosiridol derivative) 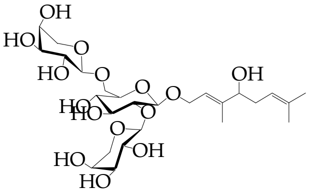 (tentative)	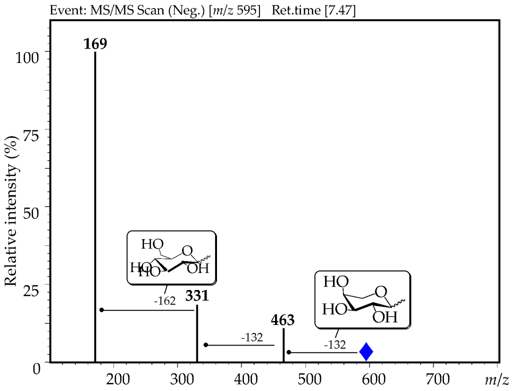
**67**	Tyrosol *O*-hexoside-di-*O*-acetate (tyrosol derivative) 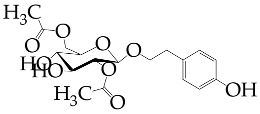 (tentative)	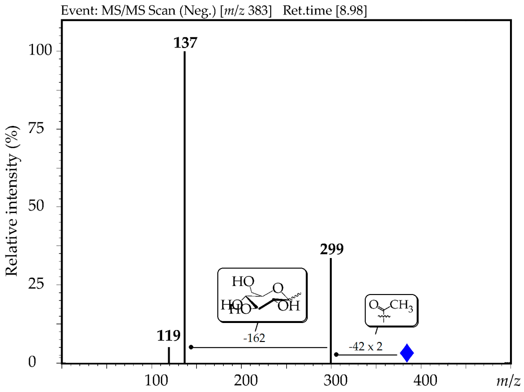
**79**	Rosiridol *O*-hexoside-di-*O*-acetate (rosiridol derivative) 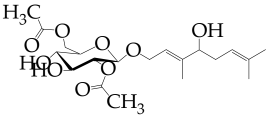 (tentative)	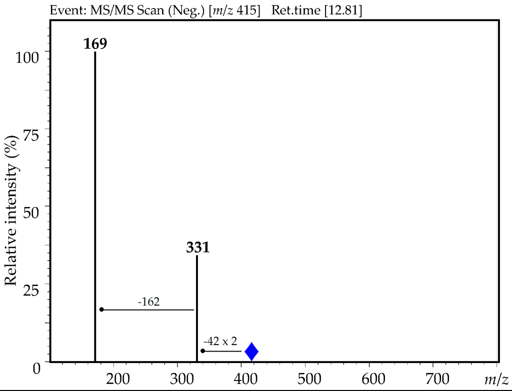

Table S6. *Cont.*

No	Compound	MS/MS, UV spectra (in windows)
**96**	Gossypetin 7-*O*-Rha-8-*O*-Glc = rhodiolgidin (neutral gossypetin 7,8-di-*O*-glycoside) 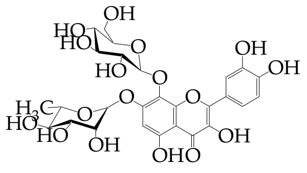	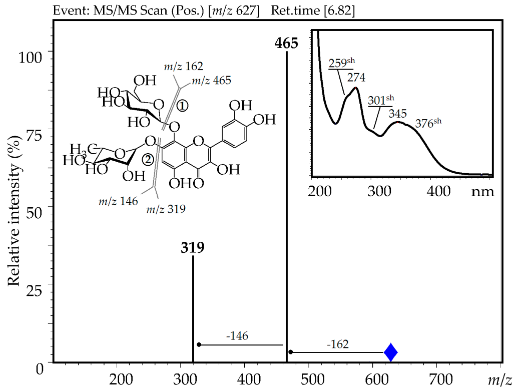
**99**	Herbacetin 7-*O*-Rha-8-*O*-Glc = rhodionidin (neutral herbacetin 7,8-di-*O*-glycoside) 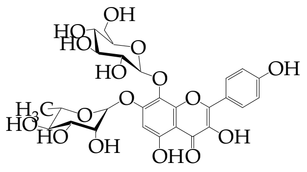	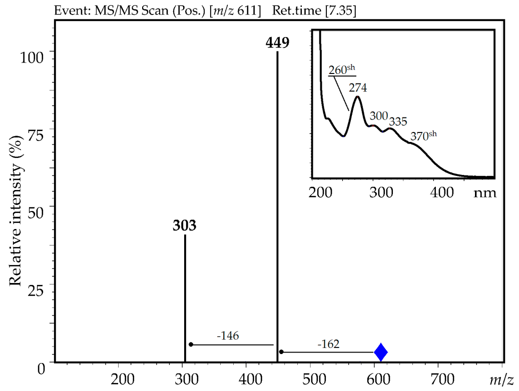
**112**	Herbacetin 7-*O*-Rha = rhodionin (neutral herbacetin 7-*O*-glycoside) 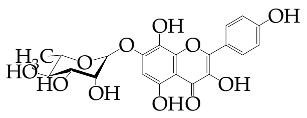	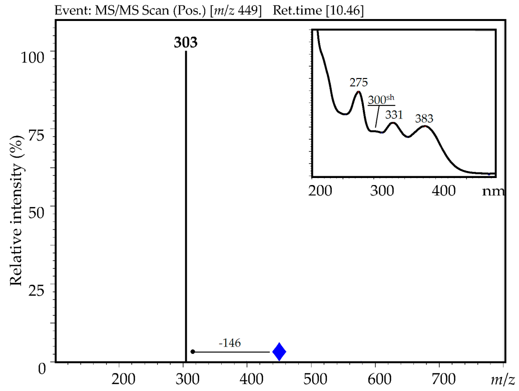
**116**	Gossypetin di-*O*-hexoside-*O*-hexuronide (acidic gossypetin 3,7,8-tri-*O*-glycoside) 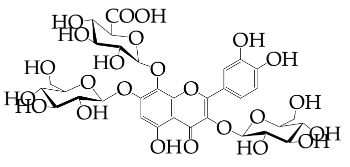 (tentative)	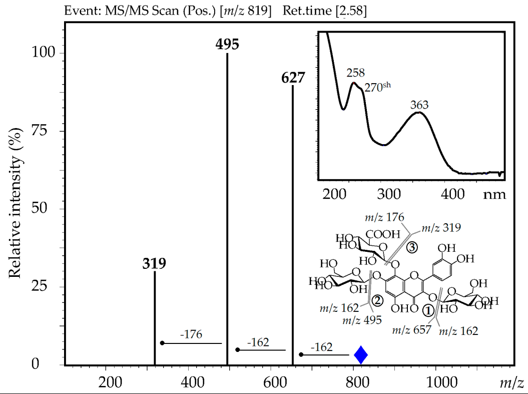

Table S6. *Cont.*

No	Compound	MS/MS, UV spectra (in windows)
**120**	Herbacetin di-*O*-hexoside-*O*-hexuronide (acidic herbacetin 3,7,8-tri-*O*-glycoside) 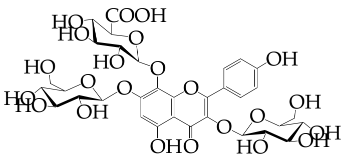 (tentative)	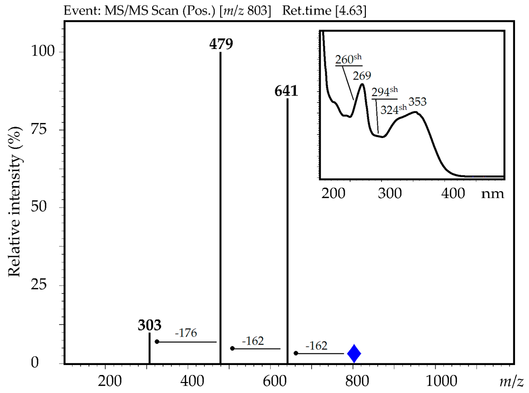
**123**	Hibiscetin *O*-hexoside-*O*-hexuronide (acidic hibiscetin 3,8-di-*O*-glycoside) 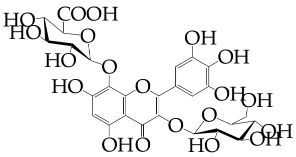 (tentative)	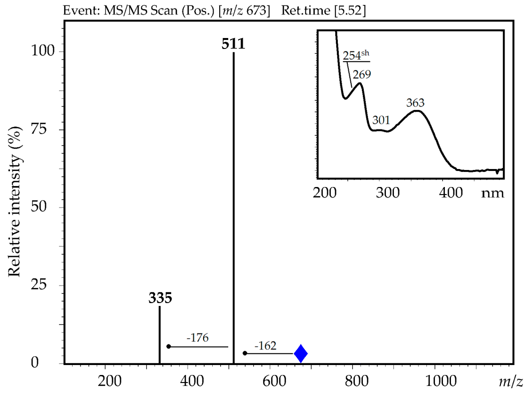
**126**	Gossypetin 3-*O*-glucoside-8-*O*-glucuronide (acidic gossypetin 3,8-di-*O*-glycoside) 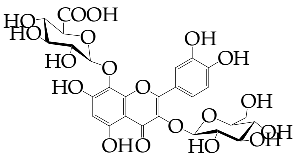	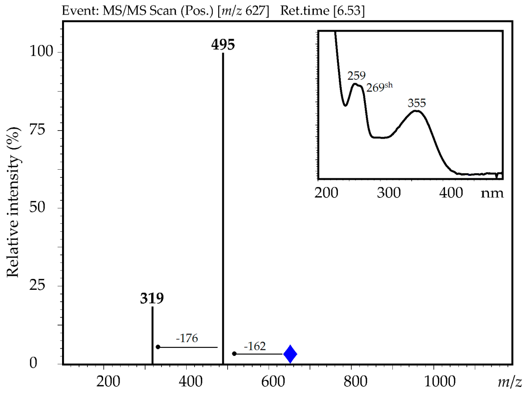
**130**	Herbacetin 3-*O*-glucoside-8-*O*-glucuronide (acidic herbacetin 3,8-di-*O*-glycoside) 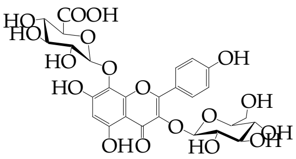	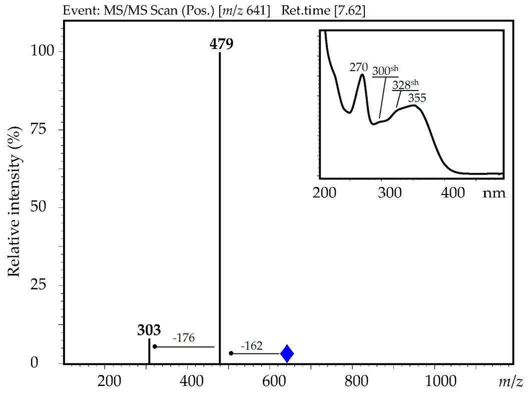

Table S6. *Cont.*

No	Compound	MS/MS, UV spectra (in windows)
**138**	Gossypetin 8-*O*-glucuronide = hibifolin (acidic gossypetin 8-*O*-glycoside) 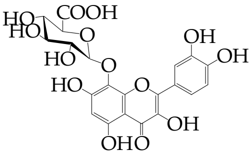	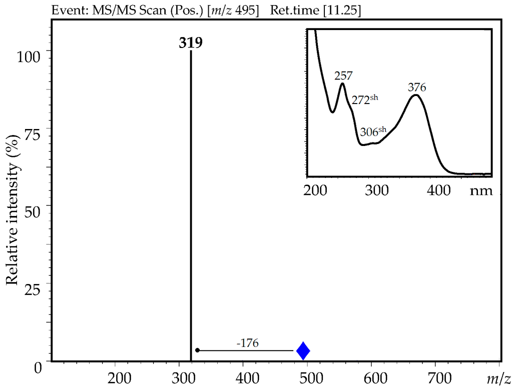
**143**	Herbacetin 8-*O*-GlcA = melocorin (acidic herbacetin 8-*O*-glycoside) 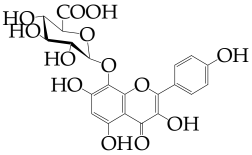	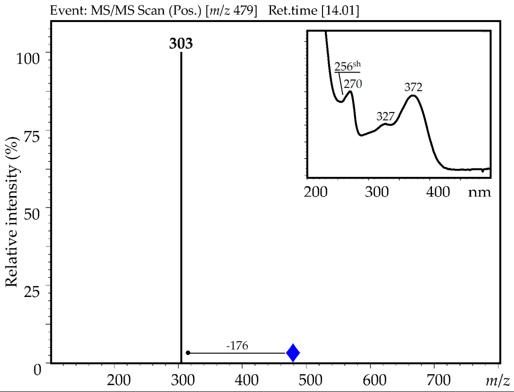

Table S7. Known flavonol glycosides of *R. rosea*.

Compound	Presence in *R. rosea* organ [ref.]		
	Roots/Rhizomes	Leaves	Flowers
Kaempferol group (9)			
Kaempferol-3-*O*-$^{\alpha L}$Rha*p* (=afzelin)	[2]		
Kaempferol-3-*O*-$^{\beta D}$Glc*p* (=astragalin)	[2]		
Kaempferol-3-*O*-(2′′-*O*-$^{\beta D}$Xyl*p*)-$^{\beta D}$Glc*p* (=leucoside)	[54]		
Kaempferol-7-*O*-$^{\alpha L}$Rha*p*	[56]		
Kaempferol-7-*O*-(2′′-*O*-$^{\alpha L}$Rha*p*)-$^{\beta D}$Glc*p*	[2]		
Kaempferol-3-*O*-$^{\alpha L}$Rha*p*-7-*O*-$^{\beta D}$Glc*p*		[15]	[15]
Kaempferol-3-*O*-$^{\beta D}$Glc*p*-7-*O*-$^{\alpha L}$Rha*p*	[54]		
Kaempferol-3-*O*-(6′′-O-$^{\alpha L}$Rha*p*)-$^{\beta D}$Gal*p*-7-*O*-$^{\alpha L}$Rha*p* (=robinin)	[2]		
Kaempferol-3,7-di-*O*-$^{\beta D}$Glc*p*		[15]	[15]
Quercetin group (2)			
Quercetin-3-*O*-$^{\alpha L}$Rha*p* (=quercitrin)	[2]		
Quercetin-3-*O*-$^{\alpha L}$Rha*p*-7-*O*-$^{\beta D}$Glc*p*		[15]	[15]
Herbacetin group (11)			
Herbacetin 8-*O*-methyl ester	[56]		
Herbacetin-7-*O*-$^{\alpha L}$Rha*p* (=rhodionin)	[55]		[13,14]
Herbacetin-7-*O*-(3′′-*O*-$^{\beta D}$Glc*p*)-$^{\alpha L}$Rha*p* (=rhodiosin)	[55]		
Herbacetin-8-*O*-$^{\beta D}$Xyl*p* (=rhodalin)		[14]	[14]
Herbacetin-8-*O*-(3′′-*O*-Ac)-$^{\beta D}$Xyl*p* (=acetylrhodalin)	[56]		
Herbacetin-8-*O*-$^{\beta D}$Glc*p* (=alginin, herbacin)	[54]		
Herbacetin-3-*O*-$^{\beta D}$Glc*p*-7-*O*-$^{\alpha L}$Ara*p*		[15]	[15]
Herbacetin-3-*O*-$^{\beta D}$Glc*p*-7-*O*-$^{\beta D}$Xyl*p*		[15]	[15]
Herbacetin-3-*O*-$^{\beta D}$Glc*p*-7-*O*-$^{\alpha L}$Rha*p* (=sinocrassoside C1)	[54]		
Herbacetin-3-*O*-$^{\beta D}$Glc*p*-8-*O*-$^{\beta D}$Xyl*p* (=rhodalidin)		[13,14]	
Herbacetin-7-*O*-$^{\alpha L}$Rha*p*-8-*O*-$^{\beta D}$Glc*p* (=rhodionidin)		[13,14]	[13,14]
Gossypetin group (5)			
Gossypetin-7-*O*-$^{\alpha L}$Rha*p* (=rhodiolgin)			[13,14]
Gossypetin-7-*O*-(3′′-*O*-$^{\beta D}$Glc*p*)-$^{\alpha L}$Rha*p* (=rhodioflavonoside)	[53]		
Gossypetin-3-*O*-$^{\beta D}$Glc*p*-7-*O*-$^{\alpha L}$Ara*p*		[15]	[15]
Gossypetin-3-*O*-$^{\beta D}$Glc*p*-7-*O*-$^{\beta D}$Xyl*p*		[15]	[15]
Gossypetin-7-*O*-$^{\alpha L}$Rha*p*-8-*O*-$^{\beta D}$Glc*p* (=rhodiolgidin)		[13,14]	[13,14]
Tricin group (2)			
Tricin-5-*O*-$^{\beta D}$Glc*p*	[70]		
Tricin-7-*O*-$^{\beta D}$Glc*p*	[70]		

Ac—acetyl; $^{\alpha L}$Ara*p*—α-L-arabinopyranose; $^{\beta D}$Gal*p*—β-D-galactopyranose; $^{\beta D}$Glc*p*—β-D-glucopyranose; $^{\alpha L}$Rha*p*—α-L-rhamnopyranose; $^{\beta D}$Xyl*p*—β-D-xylopyranose.

Table S8. Quantitative content of compounds **1–146** in *R. rosea* organs, mg/g dry plant weight ± S.D. [a]

No	Compound [b]	Roots	Rhizome	Leaves	Flowers	Stems
**1**	1-*O*-Galloyl glucose	0.32 ± 0.01	0.09 ± 0.00	0.12 ± 0.00	0.12 ± 0.00	0.10 ± 0.00
**2**	*O*-Galloyl glucose	0.29 ± 0.00	0.21 ± 0.00	0.14 ± 0.00	0.36 ± 0.01	0.09 ± 0.00
**3**	*O*-Galloyl glucose	n.d.	n.d.	0.79 ± 0.02	n.d.	0.10 ± 0.00
**4**	*O*-Galloyl glucose	n.d.	n.d.	n.d.	n.d.	0.31 ± 0.00
**5**	Gallic acid	1.72 ± 0.03	1.19 ± 0.02	1.67 ± 0.03	1.52 ± 0.03	0.27 ± 0.00
**6**	Di-*O*-galloyl glucose	traces	traces	n.d.	n.d.	n.d.
**7**	1,6-Di-*O*-galloyl glucose	2.29 ± 0.04	1.17 ± 0.02	10.35 ± 0.21	1.56 ± 0.03	3.89 ± 0.06
**8**	Procyanidin dimer (EGC-EGC)	0.02 ± 0.00	0.34 ± 0.00	n.d.	0.19 ± 0.00	0.15 ± 0.00
**9**	Gallocatechin	0.29 ± 0.00	2.08 ± 0.04	n.d.	n.d.	0.12 ± 0.00
**10**	Epigallocatechin	0.12 ± 0.00	1.36 ± 0.02	n.d.	n.d.	n.d.
**11**	Procyanidin dimer (EGC-EGCG)	3.48 ± 0.07	14.55 ± 0.27	n.d.	traces	n.d.
**12**	Catechin	1.52 ± 0.03	4.11 ± 0.07	n.d.	n.d.	n.d.
**13**	Tri-*O*-galloyl glucose	n.d.	n.d.	0.64 ± 0.01	n.d.	0.45 ± 0.01
**14**	Procyanidin dimer (EGCG-EGCG)	traces	0.59 ± 0.01	n.d.	n.d.	n.d.
**15**	Procyanidin trimer (EGC-EGC-EGC)	0.11 ± 0.00	0.67 ± 0.01	n.d.	n.d.	n.d.
**16**	Procyanidin trimer (EGC-EGC-EGCG)	3.36 ± 0.06	10.68 ± 0.22	n.d.	n.d.	n.d.
**17**	1,3,6-Tri-*O*-galloyl glucose	n.d.	n.d.	1.31 ± 0.02	n.d.	0.27 ± 0.00
**18**	Tri-*O*-galloyl glucose	0.83 ± 0.02	0.42 ± 0.01	n.d.	n.d.	0.32 ± 0.00
**19**	Procyanidin trimer (EGC-EGCG-EGCG)	traces	0.49 ± 0.01	n.d.	n.d.	n.d.
**20**	Procyanidin trimer (EGCG-EGCG-EGCG)	traces	0.45 ± 0.01	n.d.	n.d.	n.d.
**21**	Procyanidin tetramer (EGC-EGC-EGC-EGCG)	traces	0.22 ± 0.00	n.d.	n.d.	n.d.
**22**	Epicatechin	4.47 ± 0.09	19.08 ± 0.38	n.d.	n.d.	n.d.
**23**	Epigallocatechin gallate	1.48 ± 0.03	9.48 ± 0.19	n.d.	n.d.	n.d.
**24**	Gallocatechin gallate	1.89 ± 0.04	12.44 ± 0.25	n.d.	n.d.	n.d.
**25**	Procyanidin tetramer (EGC-EGC-EGCG-EGCG)	traces	traces	n.d.	n.d.	n.d.
**26**	Procyanidin tetramer (EGC-EGCG-EGCG-EGCG)	0.10 ± 0.00	0.68 ± 0.01	n.d.	n.d.	n.d.
**27**	Tetra-*O*-galloyl glucose	n.d.	n.d.	n.d.	1.94 ± 0.04	n.d.
**28**	1,2,3,6-Tetra-*O*-galloyl glucose	n.d.	n.d.	4.77 ± 0.09	4.38 ± 0.08	1.42 ± 0.03
**29**	Tetra-*O*-galloyl glucose	n.d.	n.d.	n.d.	1.67 ± 0.04	n.d.
**30**	Epicatechin gallate	0.50 ± 0.01	5.12 ± 0.10	n.d.	n.d.	n.d.
**31**	Tetra-*O*-galloyl glucose	n.d.	n.d.	n.d.	traces	n.d.
**32**	Penta-*O*-galloyl glucose	n.d.	n.d.	7.15 ± 0.14	5.97 ± 0.11	0.72 ± 0.02
**33**	1,2,3,4,6-Penta-*O*-galloyl glucose	n.d.	n.d.	18.45 ± 0.37	6.24 ± 0.12	0.22 ± 0.00
**34**	Hexa-*O*-galloyl glucose	n.d.	n.d.	37.79 ± 0.74[c]	24.02 ± 0.49[c]	2.22 ± 0.04[c]
**35**	Hexa-*O*-galloyl glucose	n.d.	n.d.			
**36**	Catechin gallate	0.31 ± 0.00	3.73 ± 0.07	n.d.	n.d.	n.d.
**37**	Hepta-*O*-galloyl glucose	n.d.	n.d.	17.45 ± 0.34[d]	19.10 ± 0.37[d]	0.51 ± 0.01[d]
**38**	Hepta-*O*-galloyl glucose	n.d.	n.d.			
**39**	Octa-*O*-galloyl glucose	n.d.	n.d.	n.d.	0.70 ± 0.02	n.d.
**40**	Octa-*O*-galloyl glucose	n.d.	n.d.	n.d.	0.61 ± 0.01	0.09 ± 0.00
**41**	Procyanidin tetramer (EGCG-EGCG-EGCG-EGCG)	0.14 ± 0.00	0.24 ± 0.00	n.d.	1.10 ± 0.02	0.08 ± 0.00
**42**	Procyanidin pentamer (EGC-EGC-EGC-EGC-EGCG)	traces	1.39 ± 0.02	n.d.	1.34 ± 0.02	n.d.
**43**	Procyanidin pentamer (EGC-EGC-EGC-EGCG-EGCG)	0.52 ± 0.01	1.79 ± 0.03	n.d.	traces	n.d.
**44**	Procyanidin pentamer (EGC-EGC-EGCG-EGCG-EGCG)	1.04 ± 0.02	2.72 ± 0.05	n.d.	0.39 ± 0.00	n.d.
**45**	Hydroquinone *O*-Hex-*O*-Pent	0.52 ± 0.01	2.92 ± 0.06	6.27 ± 0.14	11.39 ± 0.22	traces
**46**	Hydroquinone *O*-Glc (=arbutin)	4.12 ± 0.08	5.95 ± 0.11	10.35 ± 0.21	12.65 ± 0.25	2.67 ± 0.05
**47**	Tyrosol *O*-Hex-*O*-Pent	0.93 ± 0.02	2.95 ± 0.05	0.09 ± 0.00	0.59 ± 0.01	traces
**48**	Tyrosol *O*-Glc (=salidroside)	1.04 ± 0.02	15.06 ± 0.29	0.53 ± 0.01	0.94 ± 0.02	traces
**49**	Tyrosol *O*-dHex	n.d.	n.d.	traces	traces	traces
**50**	Tyrosol (=*p*-hydroxyphenethyl alcohol)	traces	1.54 ± 0.03	traces	traces	traces
**51**	Benzyl alcohol *O*-Hex-*O*-Pent	traces	traces	traces	traces	traces
**52**	Benzyl alcohol *O*-Hex-*O*-Pent	traces	traces	traces	traces	traces
**53**	Benzyl alcohol *O*-Hex	traces	traces	traces	traces	traces
**54**	*p*-Hydroxycinnamyl alcohol *O*-Hex-*O*-Pent	traces	3.44 ± 0.07	n.d.	n.d.	n.d.
**55**	*p*-Hydroxycinnamyl alcohol *O*-Glc (=triandrin)	traces	2.86 ± 0.05	0.58 ± 0.01	1.10 ± 0.02	0.39 ± 0.00
**56**	Benzyl alcohol	traces	traces	traces	traces	traces
**57**	Rhodiocyanoside A/D *O*-Pent	traces	traces	n.d.	n.d.	n.d.
**58**	Rhodiocyanoside D	traces	traces	traces	traces	traces
**59**	Rhodiocyanoside A	0.97 ± 0.02	2.64 ± 0.05	1.42 ± 0.03	2.66 ± 0.05	0.58 ± 0.01
**60**	Rosiridol di-*O*-Hex-*O*-Pent	n.d.	n.d.	traces	traces	traces
**61**	Rosiridol *O*-Hex-di-*O*-Pent	n.d.	n.d.	traces	traces	traces
**62**	Rosiridol *O*-Hex-di-*O*-Pent	n.d.	n.d.	n.d.	n.d.	n.d.
**63**	Rosiridol *O*-Hex-*O*-Pent	traces	2.07 ± 0.04	traces	1.20 ± 0.02	traces
**64**	Rosiridol *O*-Hex-*O*-Pent	traces	3.11 ± 0.06	0.53 ± 0.01	1.83 ± 0.03	traces
**65**	Tyrosol *O*-Hex-*O*-Ac	traces	0.57 ± 0.01	traces	traces	traces
**66**	Tyrosol *O*-Hex-*O*-Ac	n.d.	n.d.	traces	0.09 ± 0.00	traces
**67**	Tyrosol *O*-Hex-di-*O*-Ac	n.d.	n.d.	traces	0.25 ± 0.00	traces
**68**	Cinnamyl alcohol *O*-Hex-di-*O*-Pent	traces	0.85 ± 0.01	n.d.	n.d.	n.d.
**69**	Cinnamyl alcohol *O*-Hex-di-*O*-Pent	traces	traces	n.d.	n.d.	n.d.
**70**	Cinnamyl alcohol *O*-(6′-*O*-Ara*f*)-Glc (=rosarin)	6.03 ± 0.12	14.20 ± 0.27	n.d.	n.d.	n.d.
**71**	Cinnamyl alcohol *O*-(6′-*O*-Ara*p*)-Glc (=rosavin)	10.46 ± 0.21	22.95 ± 0.45	n.d.	n.d.	n.d.

Table S8. *Cont.*

No	Compound	Roots	Rhizome	Leaves	Flowers	Stems
**72**	Cinnamyl alcohol *O*-Hex-*O*-Pent	0.29 ± 0.00	1.54 ± 0.03	n.d.	n.d.	n.d.
**73**	Cinnamyl alcohol *O*-Glc (=rosin)	3.11 ± 0.06	7.88 ± 0.15	n.d.	n.d.	n.d.
**74**	Rosiridol 1-*O*-Glc (=rosiridin)	traces	traces	traces	0.86 ± 0.02	traces
**75**	*p*-Hydroxycinnamyl alcohol *O*-Hex-*O*-Ac	n.d.	n.d.	traces	0.11 ± 0.00	traces
**76**	*p*-Hydroxycinnamyl alcohol *O*-Hex *O*-Ac	n.d.	n.d.	traces	0.06 ± 0.00	traces
**77**	Rosiridol *O*-Hex-*O*-Ac	n.d.	n.d.	traces	1.42 ± 0.03	traces
**78**	Rosiridol *O*-Hex-di-*O*-Ac	n.d.	n.d.	traces	0.46 ± 0.01	traces
**79**	Rosiridol *O*-Hex-di-*O*-Ac	n.d.	n.d.	traces	0.68 ± 0.01	traces
**80**	*p*-Methoxycinnamyl alcohol *O*-Hex-*O*-Pent	traces	0.89 ± 0.02	n.d.	n.d.	n.d.
**81**	*p*-Methoxycinnamyl alcohol *O*-Hex-*O*-Pent	traces	traces	n.d.	n.d.	n.d.
**82**	*p*-Methoxycinnamyl alcohol *O*-Glc (=vimalin)	traces	traces	n.d.	n.d.	n.d.
**83**	Cinnamyl alcohol	traces	traces	n.d.	n.d.	n.d.
**84**	Cinnamic acid	traces	traces	n.d.	n.d.	n.d.
**85**	*p*-Methoxycinnamyl alcohol	traces	1.15 ± 0.02	n.d.	n.d.	n.d.
**86**	Kaempferol tri-*O*-Hex-*O*-dHex (S37)	n.d.	n.d.	n.d.	traces	n.d.
**87**	Herbacetin tri-*O*-Hex-*O*-dHex (S78)	n.d.	n.d.	traces	traces	n.d.
**88**	Gossypetin di-*O*-Hex-*O*-dHex (S78)	n.d.	n.d.	traces	traces	n.d.
**89**	Quercetin di-*O*-Hex-*O*-dHex (S37)	n.d.	n.d.	traces	traces	n.d.
**90**	Kaempferol di-*O*-Hex-*O*-dHex (S37)	traces	traces	traces	traces	n.d.
**91**	Gossypetin di-*O*-Hex (S8)	n.d.	n.d.	n.d.	traces	n.d.
**92**	Herbacetin di-*O*-Hex-*O*-dHex (S78)	n.d.	n.d.	traces	traces	n.d.
**93**	Gossypetin di-*O*-Hex (S38)	n.d.	n.d.	n.d.	traces	n.d.
**94**	Gossypetin 7-*O*-(3′′-*O*-Glc)-Rha (=rhodioflavonoside)	traces	traces	traces	traces	n.d.
**95**	Herbacetin 7-*O*-(3′′-*O*-Glc)-Rha (=rhodiosin)	n.d.	n.d.	traces	1.32 ± 0.02	n.d.
**96**	Gossypetin 7-*O*-Rha-8-*O*-Glc (=rhodiolgidin)	n.d.	n.d.	traces	17.62 ± 0.35	0.35 ± 0.00
**97**	Kaempferol *O*-Hex-*O*-dHex (S7)	n.d.	n.d.	traces	traces	n.d.
**98**	Herbacetin-8-*O*-Xyl (=rhodalin)	0.10 ± 0.00	0.02 ± 0.00	traces	traces	n.d.
**99**	Herbacetin 7-*O*-Rha-8-*O*-Glc (=rhodionidin)	traces	traces	traces	20.42 ± 0.41	1.39 ± 0.02
**100**	Quercetin 3,7-di-*O*-Glc	n.d.	n.d.	n.d.	traces	n.d.
**101**	Kaempferol 3,7-di-*O*-Glc	traces	traces	n.d.	traces	n.d.
**102**	Quercetin 3-*O*-Glc-7-*O*-Rha	n.d.	n.d.	traces	traces	n.d.
**103**	Kaempferol 3-*O*-Glc-7-*O*-Rha	traces	traces	traces	0.09 ± 0.00	n.d.
**104**	Gossypetin 8-*O*-Glc (=gossypin)	n.d.	n.d.	n.d.	traces	n.d.
**105**	Kaempferol 3,7-di-*O*-Rha (=kaempferitrin)	n.d.	n.d.	n.d.	traces	n.d.
**106**	Gossypetin 7-*O*-Rha (=rhodiolgin)	n.d.	n.d.	n.d.	traces	n.d.
**107**	Quercetin 3-*O*-Glc (=isoquercitrin)	traces	traces	traces	0.08 ± 0.00	n.d.
**108**	Quercetin 3-*O*-Rha (=quercitrin)	traces	traces	n.d.	traces	n.d.
**109**	Herbacetin 8-*O*-Glc (=herbacin)	traces	traces	traces	traces	n.d.
**110**	Herbacetin *O*-dHex (S8)	traces	traces	n.d.	1.46 ± 0.03	n.d.
**111**	Kaempferol 3-*O*-Rha (=afzelin)	0.36 ± 0.00	0.06 ± 0.00	n.d.	0.12 ± 0.00	n.d.
**112**	Herbacetin 7-*O*-Rha (=rhodionin)	traces	traces	n.d.	1.77 ± 0.04	n.d.
**113**	Herbacetin *O*-dHex (S3)	n.d.	n.d.	n.d.	traces	n.d.
**114**	Kaempferol 7-*O*-Rha	0.10 ± 0.00	traces	n.d.	traces	n.d.
**115**	Hibiscetin di-*O*-Hex-*O*-HexA (S38)	n.d.	n.d.	traces	traces	n.d.
**116**	Gossypetin di-*O*-Hex-*O*-HexA (S378)	n.d.	n.d.	0.55 ± 0.01	traces	n.d.
**117**	Gossypetin *O*-Ac-di-*O*-Hex-*O*-HexA (S378)	n.d.	n.d.	traces	n.d.	traces
**118**	Gossypetin *O*-Ac-di-*O*-Hex-*O*-HexA (S378)	n.d.	n.d.	traces	traces	n.d.
**119**	Herbacetin di-*O*-Hex-*O*-HexA (S38)	n.d.	n.d.	traces	traces	n.d.
**120**	Herbacetin di-*O*-Hex-*O*-HexA (S378)	traces	traces	10.35 ± 0.21	n.d.	traces
**121**	Herbacetin *O*-Mal-di-*O*-Hex-*O*-HexA (S378)	traces	traces	2.11 ± 0.04	traces	traces
**122**	Herbacetin *O*-Ac-di-*O*-Hex-*O*-HexA (S378)	n.d.	n.d.	traces	n.d.	traces
**123**	Hibiscetin *O*-Hex-*O*-HexA (S38)	traces	traces	0.89 ± 0.02	0.92 ± 0.02	0.07 ± 0.00
**124**	Hibiscetin *O*-Mal-*O*-Hex-*O*-HexA (S38)	n.d.	n.d.	traces	traces	traces
**125**	Hibiscetin *O*-Ac-*O*-Hex-*O*-HexA (S38)	n.d.	n.d.	traces	n.d.	traces
**126**	Gossypetin 3-*O*-Glc-8-*O*-GlcA	0.19 ± 0.00	0.08 ± 0.00	1.42 ± 0.03	2.33 ± 0.05	0.37 ± 0.00
**127**	Gossypetin 3-*O*-(3′′-*O*-Ac)-Glc-8-*O*-GlcA (=rhodiquadrin B)	traces	traces	traces	traces	traces
**128**	5-*O*-Feruloylquinic acid	traces	traces	traces	traces	traces
**129**	Herbacetin 8-*O*-(2′′-*O*-Glc)-GlcA (=rhodiquadrin C)	traces	traces	traces	traces	traces
**130**	Herbacetin 3-*O*-Glc-8-*O*-GlcA	traces	traces	10.52 ± 0.22	4.96 ± 0.10	1.14 ± 0.02
**131**	Herbacetin *O*-Ac-*O*-Hex-*O*-HexA (S3,8)	n.d.	n.d.	traces	n.d.	n.d.
**132**	Herbacetin 3-*O*-(3′′-*O*-Ac)-Glc-8-*O*-GlcA	0.16 ± 0.00	0.07 ± 0.00	0.38 ± 0.00	0.37 ± 0.00	traces
**133**	Gossypetin di-*O*-Ac-*O*-Hex-*O*-HexA (S3,8)	0.09 ± 0.00	0.03 ± 0.00	traces	n.d.	traces
**134**	Gossypetin di-*O*-Ac-*O*-Hex-*O*-HexA (S3,8)	traces	traces	traces	traces	traces
**135**	Herbacetin *O*-Ac-*O*-Hex-*O*-HexA (S3,8)	traces	traces	traces	0.49 ± 0.01	0.95 ± 0.02
**136**	Herbacetin *O*-Ac-*O*-Hex-*O*-HexA (S3,8)	traces	traces	traces	traces	traces
**137**	Herbacetin di-*O*-Ac-*O*-Hex-*O*-HexA (S3,8)	traces	traces	0.63 ± 0.02	0.40 ± 0.01	traces
**138**	Gossypetin 8-*O*-GlcA (=hibifolin)	n.d.	n.d.	traces	n.d.	traces
**139**	Gossypetin *O*-Mal-*O*-HexA (S8)	n.d.	n.d.	traces	0.65 ± 0.02	n.d.
**140**	Gossypetin *O*-Mal-*O*-HexA (S8)	traces	traces	traces	n.d.	n.d.
**141**	Gossypetin *O*-Ac-*O*-HexA (S8)	traces	traces	traces	traces	n.d.
**142**	Gossypetin *O*-Ac-*O*-HexA (S8)	n.d.	n.d.	traces	traces	n.d.

Table S8. *Cont.*

No	Compound	Roots	Rhizome	Leaves	Flowers	Stems
**143**	Herbacetin 8-*O*-GlcA (=melocorin)	0.55 ± 0.01	0.19 ± 0.00	traces	2.55 ± 0.05	2.03 ± 0.04
**144**	Herbacetin *O*-Mal-*O*-HexA (S8)	0.14 ± 0.00	0.05 ± 0.00	traces	traces	n.d.
**145**	Herbacetin *O*-Ac-*O*-HexA (S8)	traces	traces	traces	n.d.	n.d.
**146**	Herbacetin *O*-Ac-*O*-HexA (S8)	n.d.	n.d.	traces	n.d.	n.d.
	Total galloyl glucoses	5.45	3.08	100.63	68.19	10.98
	incl. mono-galloyl glucoses	0.61	0.30	1.05	0.48	0.60
	di-galloyl glucoses	2.29	1.17	10.35	1.56	3.89
	tri-galloyl glucoses	0.83	0.42	1.95	0.00	1.04
	tetra-galloyl glucoses	0.00	0.00	4.77	7.99	1.42
	penta-galloyl glucoses	0.00	0.00	25.60	12.21	0.94
	hexa-galloyl glucoses	0.00	0.00	37.79	24.02	2.22
	hepta-galloyl glucoses	0.00	0.00	17.45	19.10	0.51
	octa-galloyl glucoses	0.00	0.00	0.00	1.31	0.09
	Total catechins	10.58	57.40	0.00	0.00	0.12
	Total procyanidins	8.77	34.81	0.00	3.02	0.23
	incl. dimers	3.50	15.48	0.00	0.19	0.15
	trimers	3.47	12.29	0.00	0.00	0.00
	tetramers	0.24	1.14	0.00	1.10	0.00
	pentamers	1.56	5.90	0.00	1.73	0.08
	Hydroquinone glucosides	4.64	8.87	16.62	24.04	2.67
	p-Hydroxyphenethyl alcohol glucosides	1.97	20.12	0.62	1.87	traces
	Hydroxynitrile glucosides	0.97	2.64	1.42	2.66	0.58
	Rosiridol glucosides	traces	5.18	0.53	6.45	traces
	Cinnamyl alcohols glucosides	19.89	55.76	0.58	1.26	0.39
	incl. *p*-hydroxycinnamyl alcohol glucosides	traces	6.30	0.58	1.26	0.39
	cinnamyl alcohol glucosides	19.89	47.42	0.00	0.00	0.00
	p-methoxycinnamyl alcohol glucosides	traces	2.04	0.00	0.00	0.00
	Total flavonol glucosides	1.69	0.50	26.85	55.55	6.30
	incl. neutral glucoside	0.56	0.08	traces	42.88	1.74
	acidic glucoside	1.13	0.42	26.85	12.67	4.56
	incl. herbacetin glucosides	0.95	0.33	23.99	33.74	5.51
	gossypetin glucosides	0.28	0.11	1.97	20.60	0.72
	hibiscetin glucosides	0.00	0.00	0.89	0.92	0.07
	kaempferol glucosides	0.46	0.06	traces	0.21	traces
	quercetin glucosides	traces	traces	traces	0.08	traces

[a] Traces—<0.01 mg/g; n.d.—not detected. [a] Abbreviation used: Ac—acetyl; Ara*f*—arabinofuranose; Ara*p*—arabinopyranose; Glc—glucose; GlcA—glucuronic acid; EGC—epigallocatechin unit; EGCG—epigallocatechin gallate unit; Hex—hexose; HexA—hexuronic acid; Mal—malonyl; Pent—pentose; Rha—rhamnose; Xyl—xylose. Substitution type of flavonol glycoside: S3—3-*O*-substituted; S8—8-*O*-substituted; S37—3,7-di-*O*-substituted; S78—7,8-di-*O*-substituted; S38—3,8-di-*O*-substituted; S378—3,7,8-tri-*O*-substituted; S?—unknown type of *O*-substitution. [c] As a sum of compounds **34** and **35**. [d] As a sum of compounds **37** and **38**.

Table S9. Content of selected compounds in three *R. rosea* extracts, mg/g dry extract weight ± S.D.

No	Compound	Rhizome extract	Leaves extract	Flowers extract
**7**	1,6-Di-*O*-galloyl glucose	traces	48.21 ± 0.98	traces
**11**	Procyanidin dimer (EGC-EGCG)	44.68 ± 0.87	n.d.	n.d.
**16**	Procyanidin trimer (EGC-EGC-EGCG)	32.75 ± 0.64	n.d.	n.d.
**22**	Epicatechin	57.14 ± 1.16	n.d.	n.d.
**23**	Epigallocatechin gallate	27.11 ± 0.55	n.d.	n.d.
**24**	Gallocatechin gallate	37.08 ± 0.73	n.d.	n.d.
**30**	Epicatechin gallate	14.93 ± 0.27	n.d.	n.d.
**32**	Penta-*O*-galloyl glucose	n.d.	35.73 ± 0.72	22.82 ± 0.46
**33**	1,2,3,4,6-Penta-*O*-galloyl glucose	n.d.	92.26 ± 1.87	24.93 ± 0.48
**34+35**	Hexa-*O*-galloyl glucoses	n.d.	188.92 ± 3.79	95.80 ± 1.97
**37+38**	Hepta-*O*-galloyl glucoses	n.d.	85.14 ± 1.73	75.42 ± 1.42
**46**	Hydroquinone *O*-Glc (=arbutin)	18.67 ± 0.35	53.11 ± 1.03	51.82 ± 1.04
**48**	Tyrosol *O*-Glc (=salidroside)	46.94 ± 0.92	traces	traces
**70**	Cinnamyl alcohol *O*-(6′-*O*-Ara*f*)-Glc (=rosarin)	42.79 ± 0.86	n.d.	n.d.
**71**	Cinnamyl alcohol *O*-(6′-*O*-Ara*p*)-Glc (=rosavin)	69.54 ± 1.38	n.d.	n.d.
**73**	Cinnamyl alcohol *O*-Glc (=rosin)	24.27 ± 0.47	n.d.	n.d.
**96**	Gossypetin 7-*O*-Rha-8-*O*-Glc (=rhodiolgidin)	n.d.	traces	71.42 ± 1.47
**99**	Herbacetin 7-*O*-Rha-8-*O*-Glc (=rhodionidin)	traces	traces	81.73 ± 1.62
**120**	Herbacetin di-*O*-Hex-*O*-HexA (S378)	traces	50.28 ± 1.02	n.d.
**130**	Herbacetin 3-*O*-Glc-8-*O*-GlcA	traces	53.67 ± 1.09	19.74 ± 0.37
	Total galloyl glucoses	traces	450.26	218.97
	Total procyanidins	77.43	n.d.	n.d.
	Total catechins	136.26	n.d.	n.d.
	Total hydroquinone glucosides	18.67	53.11	51.82
	Total *p*-hydroxyphenethyl alcohol glucosides	46.44	traces	traces
	Total cinnamyl alcohol glucosides	136.60	n.d.	n.d.
	Total flavonol glucosides	traces	103.95	172.89

n.d.—not detected.

Table S10. Content of selected compounds of *R. rosea* rhizome extract (whole probe) before and after *in vitro* treatment by the simulated gastric and intestinal media, mg/probe ± S.D.

No	Compound	Non-treated	After gastric phase	After intestinal phase
**7**	1,6-Di-*O*-galloyl glucose	traces	n.d.	n.d.
**11**	Procyanidin dimer (EGC-EGCG)	22.15 ± 0.42	18.63 ± 0.38	1.18 ± 0.02
**16**	Procyanidin trimer (EGC-EGC-EGCG)	16.30 ± 0.31	8.63 ± 0.15	0.94 ± 0.02
**22**	Epicatechin	27.94 ± 0.54	25.63 ± 0.52	1.22 ± 0.02
**23**	Epigallocatechin gallate	13.42 ± 0.25	5.42 ± 0.11	0.18 ± 0.00
**24**	Gallocatechin gallate	18.97 ± 0.38	4.73 ± 0.08	0.23 ± 0.00
**30**	Epicatechin gallate	7.45 ± 0.14	5.14 ± 0.12	0.58 ± 0.01
**32**	Penta-*O*-galloyl glucose	n.d.	n.d.	n.d.
**33**	1,2,3,4,6-Penta-*O*-galloyl glucose	n.d.	n.d.	n.d.
**34+35**	Hexa-*O*-galloyl glucoses	n.d.	n.d.	n.d.
**37+38**	Hepta-*O*-galloyl glucoses	n.d.	n.d.	n.d.
**46**	Hydroquinone *O*-Glc (=arbutin)	9.32 ± 0.17	8.64 ± 0.17	7.11 ± 0.15
**48**	Tyrosol *O*-Glc (=salidroside)	23.48 ± 0.45	22.76 ± 0.47	20.54 ± 0.41
**70**	Cinnamyl alcohol *O*-(6′-*O*-Ara*f*)-Glc (=rosarin)	21.18 ± 0.40	20.98 ± 0.42	25.35 ± 0.51
**71**	Cinnamyl alcohol *O*-(6′-*O*-Ara*p*)-Glc (=rosavin)	34.75 ± 0.67	33.86 ± 0.69	26.18 ± 0.52
**73**	Cinnamyl alcohol *O*-Glc (=rosin)	12.09 ± 0.22	12.93 ± 0.25	15.71 ± 0.31
**96**	Gossypetin 7-*O*-Rha-8-*O*-Glc (=rhodiolgidin)	n.d.	n.d.	n.d.
**99**	Herbacetin 7-*O*-Rha-8-*O*-Glc (=rhodionidin)	traces	n.d.	n.d.
**120**	Herbacetin di-*O*-Hex-*O*-HexA (S378)	traces	n.d.	n.d.
**130**	Herbacetin 3-*O*-Glc-8-*O*-GlcA	traces	n.d.	n.d.
	Total galloyl glucoses	traces	n.d.	n.d.
	Total procyanidins	38.45	27.26	2.12
	Total catechins	67.78	40.92	2.21
	Total hydroquinone glucosides	9.32	8.64	7.11
	Total *p*-hydroxyphenethyl alcohol glucosides	23.48	22.76	20.54
	Total cinnamyl alcohol glucosides	68.02	67.77	67.24
	Total flavonol glucosides	traces	n.d.	n.d.

n.d.—not detected.

Table S11. Content of selected compounds of *R. rosea* leaves extract (whole probe) before and after *in vitro* treatment by the simulated gastric and intestinal media, mg/probe ± S.D.

No	Compound	Non-treated	After gastric phase	After intestinal phase
**7**	1,6-Di-*O*-galloyl glucose	24.02 ± 0.49	22.83 ± 0.42	15.79 ± 0.31
**11**	Procyanidin dimer (EGC-EGCG)	n.d.	n.d.	n.d.
**16**	Procyanidin trimer (EGC-EGC-EGCG)	n.d.	n.d.	n.d.
**22**	Epicatechin	n.d.	n.d.	n.d.
**23**	Epigallocatechin gallate	n.d.	n.d.	n.d.
**24**	Gallocatechin gallate	n.d.	n.d.	n.d.
**30**	Epicatechin gallate	n.d.	n.d.	n.d.
**32**	Penta-*O*-galloyl glucose	17.53 ± 0.36	5.37 ± 0.11	0.92 ± 0.02
**33**	1,2,3,4,6-Penta-*O*-galloyl glucose	46.18 ± 0.90	12.78 ± 0.23	1.40 ± 0.03
**34+35**	Hexa-*O*-galloyl glucoses	94.27 ± 1.91	25.92 ± 0.51	3.18 ± 0.06
**37+38**	Hepta-*O*-galloyl glucoses	42.49 ± 0.86	11.37 ± 0.19	0.72 ± 0.02
**46**	Hydroquinone *O*-Glc (=arbutin)	26.59 ± 0.54	24.19 ± 0.47	19.14 ± 0.37
**48**	Tyrosol *O*-Glc (=salidroside)	traces	n.d.	n.d.
**70**	Cinnamyl alcohol *O*-(6′-*O*-Ara*f*)-Glc (=rosarin)	n.d.	n.d.	n.d.
**71**	Cinnamyl alcohol *O*-(6′-*O*-Ara*p*)-Glc (=rosavin)	n.d.	n.d.	n.d.
**73**	Cinnamyl alcohol *O*-Glc (=rosin)	n.d.	n.d.	n.d.
**96**	Gossypetin 7-*O*-Rha-8-*O*-Glc (=rhodiolgidin)	traces	n.d.	n.d.
**99**	Herbacetin 7-*O*-Rha-8-*O*-Glc (=rhodionidin)	traces	n.d.	n.d.
**120**	Herbacetin di-*O*-Hex-*O*-HexA (S378)	25.09 ± 0.48	24.98 ± 0.48	24.82 ± 0.49
**130**	Herbacetin 3-*O*-Glc-8-*O*-GlcA	26.82 ± 0.53	26.70 ± 0.51	26.61 ± 0.52
	Total galloyl glucoses	224.49	78.27	22.01
	Total procyanidins	n.d.	n.d.	n.d.
	Total catechins	n.d.	n.d.	n.d.
	Total hydroquinone glucosides	26.59	24.19	19.14
	Total *p*-hydroxyphenethyl alcohol glucosides	traces	n.d.	n.d.
	Total cinnamyl alcohol glucosides	n.d.	n.d.	n.d.
	Total flavonol glucosides	51.91	51.68	51.43

n.d.—not detected.

Table S12. Content of selected compounds of *R. rosea* flowers extract (whole probe) before and after *in vitro* treatment by the simulated gastric and intestinal media, mg/probe ± S.D.

No	Compound	Non-treated	After gastric phase	After intestinal phase
**7**	1,6-Di-*O*-galloyl glucose	traces	n.d.	n.d.
**11**	Procyanidin dimer (EGC-EGCG)	n.d.	n.d.	n.d.
**16**	Procyanidin trimer (EGC-EGC-EGCG)	n.d.	n.d.	n.d.
**22**	Epicatechin	n.d.	n.d.	n.d.
**23**	Epigallocatechin gallate	n.d.	n.d.	n.d.
**24**	Gallocatechin gallate	n.d.	n.d.	n.d.
**30**	Epicatechin gallate	n.d.	n.d.	n.d.
**32**	Penta-*O*-galloyl glucose	11.40 ± 0.22	3.06 ± 0.06	0.83 ± 0.02
**33**	1,2,3,4,6-Penta-*O*-galloyl glucose	12.37 ± 0.25	3.29 ± 0.06	0.86 ± 0.02
**34+35**	Hexa-*O*-galloyl glucoses	47.63 ± 0.94	12.53 ± 0.25	2.63 ± 0.05
**37+38**	Hepta-*O*-galloyl glucoses	37.75 ± 0.76	9.43 ± 0.19	2.04 ± 0.04
**46**	Hydroquinone *O*-Glc (=arbutin)	25.73 ± 0.49	24.18 ± 0.50	19.55 ± 0.39
**48**	Tyrosol *O*-Glc (=salidroside)	traces	n.d.	n.d.
**70**	Cinnamyl alcohol *O*-(6′-*O*-Ara*f*)-Glc (=rosarin)	n.d.	n.d.	n.d.
**71**	Cinnamyl alcohol *O*-(6′-*O*-Ara*p*)-Glc (=rosavin)	n.d.	n.d.	n.d.
**73**	Cinnamyl alcohol *O*-Glc (=rosin)	n.d.	n.d.	n.d.
**96**	Gossypetin 7-*O*-Rha-8-*O*-Glc (=rhodiolgidin)	35.67 ± 0.67	35.04 ± 0.72	34.81 ± 0.69
**99**	Herbacetin 7-*O*-Rha-8-*O*-Glc (=rhodionidin)	40.59 ± 0.82	40.11 ± 0.81	39.93 ± 0.77
**120**	Herbacetin di-*O*-Hex-*O*-HexA (S378)	n.d.	n.d.	n.d.
**130**	Herbacetin 3-*O*-Glc-8-*O*-GlcA	9.82 ± 0.19	9.73 ± 0.19	9.58 ± 0.19
	Total galloyl glucoses	109.15	28.31	6.36
	Total procyanidins	n.d.	n.d.	n.d.
	Total catechins	n.d.	n.d.	n.d.
	Total hydroquinone glucosides	25.73	24.18	19.55
	Total *p*-hydroxyphenethyl alcohol glucosides	traces	n.d.	n.d.
	Total cinnamyl alcohol glucosides	n.d.	n.d.	n.d.
	Total flavonol glucosides	86.08	84.88	84.32

n.d.—not detected.
